# Vertical pathway inhibition of receptor tyrosine kinases and BAD with synergistic efficacy in triple negative breast cancer

**DOI:** 10.1038/s41698-023-00489-3

**Published:** 2024-01-10

**Authors:** Yan Qin Tan, Yi-Shiou Chiou, Hui Guo, Shuwei Zhang, Xiaoming Huang, Dukanya Dukanya, Arun M. Kumar, Shreeja Basappa, Suling Liu, Tao Zhu, Basappa Basappa, Vijay Pandey, Peter E. Lobie

**Affiliations:** 1 Institute of Biopharmaceutical and Health Engineering, Tsinghua Shenzhen International Graduate School, Tsinghua University, Shenzhen, 518055 People’s Republic of China; 2 Tsinghua Berkeley Shenzhen Institute, Tsinghua Shenzhen International Graduate School, Tsinghua University, Shenzhen, 518055 Guangdong People’s Republic of China; 3 Master Degree Program in Toxicology, College of Pharmacy, Kaohsiung Medical University, Kaohsiung, 807 Taiwan; 4 Shenzhen Bay Laboratory, Shenzhen, 518055 Guangdong People’s Republic of China; 5 Department of Medical Research, Kaohsiung Medical University Hospital, Kaohsiung, 807 Taiwan; 6 Laboratory of Chemical Biology, Department of Studies in Organic Chemistry, University of Mysore, Manasagangotri, 570006 Mysore India; 7 Fudan University Shanghai Cancer Center & Institutes of Biomedical Sciences, Shanghai Medical College, Key Laboratory of Breast Cancer in Shanghai, Innovation Center for Cell Signaling Network, Cancer Institute, Fudan University, Shanghai, People’s Republic of China; 8 Department of Oncology, The First Affiliated Hospital of USTC, Center for Advanced Interdisciplinary Science and Biomedicine of IHM, Division of Life Sciences and Medicine, University of Science and Technology of China, Hefei, Anhui People’s Republic of China; 9 Hefei National Laboratory for Physical Sciences, University of Science and Technology of China, Hefei, Anhui People’s Republic of China

**Keywords:** Breast cancer, Drug development, Targeted therapies, High-throughput screening, Target identification

## Abstract

Aberrant activation of the PI3K/AKT signaling axis along with the sustained phosphorylation of downstream BAD is associated with a poor outcome of TNBC. Herein, the phosphorylated to non-phosphorylated ratio of BAD, an effector of PI3K/AKT promoting cell survival, was observed to be correlated with worse clinicopathologic indicators of outcome, including higher grade, higher proliferative index and lymph node metastasis. The structural optimization of a previously reported inhibitor of BAD-Ser99 phosphorylation was therefore achieved to generate a small molecule inhibiting the phosphorylation of BAD at Ser99 with enhanced potency and improved oral bioavailability. The molecule 2-((4-(2,3-dichlorophenyl)piperazin-1-yl)(pyridin-3-yl)methyl) phenol (NCK) displayed no toxicity at supra-therapeutic doses and was therefore assessed for utility in TNBC. NCK promoted apoptosis and G0/G1 cell cycle arrest of TNBC cell lines in vitro, concordant with gene expression analyses, and reduced in vivo xenograft growth and metastatic burden, demonstrating efficacy as a single agent. Additionally, combinatorial oncology compound library screening demonstrated that NCK synergized with tyrosine kinase inhibitors (TKIs), specifically OSI-930 or Crizotinib in reducing cell viability and promoting apoptosis of TNBC cells. The synergistic effects of NCK and TKIs were also observed in vivo with complete regression of a percentage of TNBC cell line derived xenografts and prevention of metastatic spread. In patient-derived TNBC xenograft models, NCK prolonged survival times of host animals, and in combination with TKIs generated superior survival outcomes to single agent treatment. Hence, this study provides proof of concept to further develop rational and mechanistic based therapeutic strategies to ameliorate the outcome of TNBC.

## Introduction

With the lack of expression of estrogen receptor (ER), progesterone receptor (PR) and lack of amplification of human epidermal growth factor receptor 2 (HER2), chemotherapy is currently the main systemic therapeutic option for TNBC. Even though patients with TNBC generally showed a better response to chemotherapy than other BC subtypes, TNBC patients often exhibit a significantly different response towards conventional therapy due to heterogeneity and distinct differences in pathway activation^1,2^. The phosphoinositide 3-kinase (PI3K)/AKT pathway is among the most important intracellular signaling cascades in cancer and plays a pivotal role in linking receptor tyrosine kinases (RTKs), a transmembrane protein family with intrinsic tyrosine kinase activity, to cancer development and progression^3^. With the frequent activation of PI3K/AKT signaling^4–6^, TNBC has been reported to be sensitive to the PI3K/mTOR inhibitor NVP-BEZ235 irrespective of PIK3CA mutation or PTEN deficiency, raising the possibility of targeting this axis for treatment^7^. However, even though pre-clinical data indicate the potency of targeting the PI3K/AKT pathway in TNBC, intra-pathway feedback loops caused by single kinase inhibition along the PI3K/AKT axis and toxicity associated with PI3K/AKT/mTOR dual-blockade agents potentially limit their effectiveness in the clinic. Therefore, it is essential to identify novel therapeutic approaches to improve the prognosis of TNBC.

BCL2-associated death promoter (BAD) is a BH3-only member of the BCL-2 family governing apoptosis and BAD phosphorylation is increased in various cancers^8^. By phosphorylation at human Ser75, Ser99 and Ser118, BAD switches from pro-apoptotic functions to promotion of cell survival, by heterodimerizing with 14-3-3 protein instead of BCL-XL, BCL-2 or BCL-w^8,9^. In addition to its apoptotic function, a role of BAD in inhibiting G1 to S phase transition and CYCLIN D1 expression were previously reported^10^. Being a core downstream molecule of the PI3K/AKT and MAPK pathways, BAD phosphorylation at the Serine 99 residue, and subsequently at Serine 118, is governed by the activation of PI3K/AKT whereas BAD phosphorylation at Serine 75 residue is predominantly achieved by the MAPK pathway^11^. Not surprising given the aberrant activation of the PI3K/AKT pathway in TNBC, high pBADSer99 in TNBC has been reported to be associated with poor prognosis^9,12^. Therefore, targeting BAD phosphorylation at Ser99 independent of kinase activities^13^, offers an alternate therapeutic approach for TNBC.

Although targeted therapy has achieved advances in the understanding of cancer progression and cancer treatment, the intrinsic and acquired resistance of cancer cells has greatly limited the efficacy of a single or ‘one-target’ drug, often through the activation of compensatory signaling pathway^14^. Conversely, combination therapy, by targeting multiple pathways, yields synergistic or additive therapeutic results which exhibit significant advantages in reducing dose-limiting toxicity and minimizing drug resistance, thus attracting considerable research and clinical interest^15^. However, to date, a limited number of combination therapies are reported to be effective in the clinical setting for TNBC^16^. As gene expression profiling reported increased expression of multiple RTKs in TNBC^17–19^, RTK inhibitors (TKIs) have been of interest in TNBC treatment. However, despite initial success in TKI treatment, acquired resistance due to acquisition of new mutations and bypass pathway activation limited therapeutic efficacy in TNBC, and other cancers^20–23^. Hence, effective synergistic combination approaches to improve the therapeutic efficacy of TKIs in TNBC is warranted. In this study, the chemical synthesis, and development of 2-((4-(2,3-dichlorophenyl)piperazin-1-yl)(pyridin-3-yl)methyl) phenol (NCK) as a more potent and orally bioavailable inhibitor of pBADSer99 when compared to NPB^13^ is reported. Furthermore, synergistic targets for rational drug combinations with pBADSer99 inhibitors in the treatment of TNBC were explored by combinatorial screening approaches. TKIs targeting VEGFR and c-MET, among other kinases, were identified as highly synergistic in combination with pBADSer99 inhibition. Their synergistic actions in the treatment of TNBC in vitro and ex vivo as well as in vivo, using orthotopic and intravenous TNBC and syngeneic models, and patient-derived xenograft models of TNBC was demonstrated.

Collectively, these findings have provided proof of concept for therapeutic strategies for patients with TNBC and indicated the combined targeting of RTKs upstream and pBADSer99 downstream may be a promising avenue for TNBC therapy.

## Results

### pBADSer99 is a therapeutic vulnerability in TNBC

**Fig. 1:**
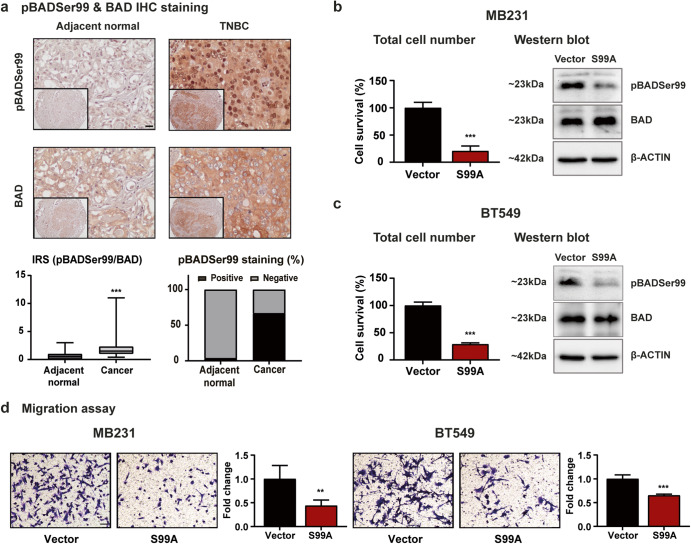
pBADSer99 is a potential therapeutic target in TNBC. **a** pBADSer99 levels and BAD expression were determined using immunohistochemistry (IHC) in adjacent normal (AD) and TNBC tissue specimens. Representative IHC images of pBADSer99 in TNBC and AD tissues (up). Scale bar, 20 μm. Analysis of the pBADSer99/BAD ratio and pBADSer99 staining (%) in AD and TNBC tissue specimens (down). For pBADSer99, the immunoreactive score (IRS) 0 to 4 was categorized as negative and IRS 5 to 12 as positive. For the pBADSer99/BAD ratio, the IRS ratio higher than 0.75 was regarded as positive^78^. Cell survival of MDA-MB-231 (**b**) and BT549 (**c**) cells after transfection with pBADS99A knock in plasmid or vector control. Data represent means ± SD (*n* = 3). *P < 0.05, **P < 0.01, and ***P < 0.001. Corresponding immunoblots displaying levels of pBADSer99 and BAD. The sizes of detected bands in kDa are shown on the left. **d** Transwell analysis was performed to determine the effect of pBADS99A knock in on cell migration of MDA-MB-231 and BT549 cells. The TNBC cells were transfected with pBADS99A plasmid or vector control. Scale bar, 50 μm. Data represent means ± SD (*n* = 5). *P < 0.05, **P < 0.01, and ***P < 0.001.

To determine whether pBADSer99 is a potential therapeutic vulnerability in TNBC, the level of pBADSer99 and expression of BAD in TNBC and adjacent normal (AD) tissue specimens were analyzed using immunohistochemistry (IHC) (Fig. 1a). TNBC specimens exhibited significantly increased expression of BAD and also increased pBADSer99 levels when compared to normal breast tissues specimens, as demonstrated by immunoreactive score (IRS) analysis (Fig. 1a, Supplementary Fig. 1A, B). Despite an increase in BAD expression, a higher pBADSer99/BAD ratio was observed in TNBC compared to normal breast tissues (Fig. 1a). A high level of pBADSer99 was observed in 67.4% (31/46) of cancer tissues, whereas in normal tissue, high expression was observed in 4.3% (2/46) of samples (Fig. 1a). In terms of subcellular localization, nuclear localization of pBADSer99 but cytoplasmic localization of BAD were observed in TNBC tissue specimens (Fig. 1a). This is consistent with a previous study which demonstrated that unlike BAD which is largely localized to cytoplasm, phosphorylated BAD, especially at Serine 99 (murine Serine 136) exhibits largely nuclear localization in cancer tissues of ER+PR+HER2+ , ER+PR+HER2- and ER-PR-HER2- BC patients^24^. The study suggested that nuclear sequestration of phosphorylated BAD might be an alternative mechanism in addition to cytoplasmic sequestration by 14-3-3 to prevent BAD’s pro-apoptotic function at the mitochondria in BC^9,24,25^.

Furthermore, the correlation between pBADSer99 level and the pBADSer99/BAD ratio in TNBC specimens and their clinicopathologic characteristics were assessed. It was observed that the pBADSer99 level was not related to age nor lymph node metastasis, but was positively correlated with lower tumor grade and higher MKI67 labeling (Supplementary Fig. 1C). However, the pBADSer99/BAD ratio in TNBC specimens was positively correlated with higher tumor grade, a higher degree of lymph node metastasis and higher MKI67 labeling (Table 1), which are independent predictors of a poor outcome in TNBC^26–28^. Therefore, the high level of pBADSer99 and pBADSer99/BAD ratio in TNBC specimens and, the correlation of pBADSer99/BAD ratio with higher tumor grade and lymph node metastasis indicates an actionable vulnerability enabling targeting of BADSer99 phosphorylation in TNBC. To functionally correlate the association of pBADSer99 with clinicopathologic features of TNBC, homology-directed repair (HDR) was utilized to replace Ser99 with alanine generating hBADS99A (Supplementary Fig. 2) in MDA-MB-231 and BT549 cells, two TNBC cell lines. When compared to control cells, western blot analysis indicated that protein expression of total BAD was not altered but the pBADSer99 level and pBADSer99/BAD ratio were decreased (Fig. 1b, c, Supplementary Fig. 3A). Homology directed repair of hBAD to hBADS99A significantly reduced cell viability in MDA-MB-231 (Fig. 1b) and BT549 (Fig. 1c) cells when compared to vector control. In addition, consistent with the positive association of pBADSer99/BAD level with lymph node metastasis in TNBC specimens (Table 1), decreased migrative capacity was observed in hBADS99A-transfected MDA-MB-231 and BT549 cells when compared to the vector-transfected cells by transwell assay (Fig. 1d) and real-time migration assay (Supplementary Fig. 3B, C). Collectively, these results indicate that phosphorylation of BADSer99 is essential for TNBC cell survival and is a potential therapeutic target for this subtype of TNBC.

**Table 1:** Clinicopathological analysis.

Cohort	Total (N)	Positive (%)	Negative (%)	*P*-value
**Age**				**0.647**
<=55	18	33	67	
>55	28	29	71	
**Grade**				<0.0001***
I	19	32	68	
II	24	25	75	
III	3	67	33	
**Lymph Node Metastasis**				<0.0001***
0	28	36	64	
1	6	0	100	
2	4	75	25	
3	3	0	100	
**MKI67**				<0.008**
Low	10	20	80	
Moderate	21	29	71	
Strong	15	40	60	

*P < 0.05, **P < 0.01, and ***P < 0.001.

Correlation analysis between pBADSer99/BAD ratio and clinicopathological features of TNBC patient.

### Generation of a small molecule pBADSer99 inhibitor with improved potency

**Fig. 2:**
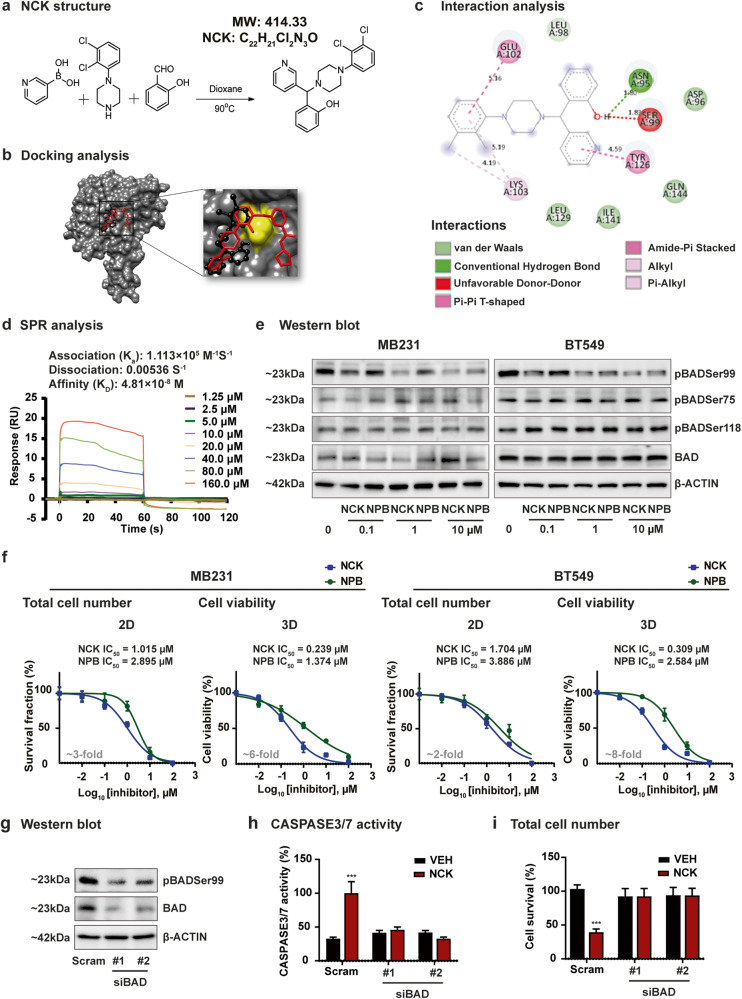
pBADSer99 inhibitor NCK demonstrates improved potency over NPB. **a** Petasis reaction, a three component boronic Mannich-type reaction which utilizes boronic acids as a potential nucleophilic species, salicylaldehyde, and substituted piperazines to form the new C–C bond of the formula I compound, was utilized to synthesize NCK (C_22_H_21_Cl_2_N_3_O). **b** 3D surface and enlarged view of the docked compounds NPB (red) & NCK (black) with the BAD protein (dim grey). The yellow color indicates the site of the Serine 99 residue. **c** 2D structure representation of NCK interacting with BAD protein residues. **d** Sensorgrams obtained by SPR analysis of NCK with the BAD protein. BAD protein was immobilized on the surface of a CM5 sensor chip. A solution of NCK at variable concentrations (1.25–160 μM) was injected to generate the binding responses (RU) recorded as a function of time (s). The results were analyzed using BIA evaluation 4.1. **e** Western Blot analysis was used to assess the level of BAD phosphorylation at Ser99, Ser75 and Ser118 in TNBC cells after treatment with NCK and NPB. β-ACTIN was used as input control for cell lysate. The sizes of detected bands in kDa are shown on the left. **f** Dose-dependent effect of NCK and NPB in 2D and 3D culture on MDA-MB-231 and BT549 TNBC cells measured by using total cell number and AlamarBlue assay respectively (*n* = 3). **g** Western Blot analysis was used to assess the level of BAD phosphorylation at Ser99 in TNBC cells after transfection with siRNA-BAD. β-ACTIN was used as input control for cell lysate. The sizes of detected bands in kDa are shown on the left. **h** CASPASE3/7 activities of MDA-MB-231 cells after transfecting with siRNA targeting BAD transcript or scrambled control and treated with 5 μM NCK were evaluated using the Biovision Caspase 3/7 DEVD Assay Kit. Data represent means ± SD (*n* = 3). *P < 0.05, **P < 0.01, and ***P < 0.001. **i** Cell survival of MDA-MB-231 cells after transfecting with siRNA targeting BAD transcript or scrambled control and treated with 5 μM NCK. Data represent means ± SD (*n* = 3). *P < 0.05, **P < 0.01, and ***P < 0.001.

The efficacy of a pBADSer99 small molecule inhibitor (NPB) in inducing apoptotic cell death in vitro in various human cancer cell lines and in vivo, independent of AKT signaling, was previously demonstrated^13^. The efficacy of NPB in combination with cisplatin^29^ and PARP inhibitors in ovarian carcinoma (OC)^30^ and in PTEN-deficient endometrial carcinoma (EC)^31^ were also recently reported. Herein, the synthesis and characterization of a NPB analog, NCK, was carried out based on the Petasis borono-Mannich multicomponent reaction using 1-(2,3-dichlorophenyl)piperazine, salicylaldehyde, and 3-pyridine-boronic acid to generate a more potent and orally bioavailable pBADSer99 inhibitor (Fig. 2a, Supplementary Fig. 4, Supplementary Fig. 5A). Using NPB as reference, bioinformatic analysis of NCK was performed with the reported crystal structure of 14-3-3 complexed with BAD (PDB ID: 7Q16) retrieved from Protein Data Bank. When compared to NPB (binding affinity of -6.51 kcal/mol), the NCK molecule exhibited a higher binding affinity of -5.68 kcal/mol to BAD (Fig. 2b, c, Supplementary Fig. 5B, C). Additionally, the frontier molecular orbital’s (FMO) energy gap (∆E_LUMO-HOMO_) of NCK was 2.42 eV and the electrophilicity index (ψ), which demonstrates the binding ability of the compound to biomolecules, was 14.86 eV (Supplementary Fig. 5D, E).

To compare the binding affinity of NCK and NPB towards BAD, we performed surface plasmon resonance (SPR) measurement by immobilizing BAD protein on a sensor chip with NCK or NPB as analyte. Representative reference-subtracted overlaid sensorgrams and the kinetic parameters are specified in Fig. 2d and Supplementary Fig. 6A. Notably, as demonstrated by the yielded dissociation equilibrium constant (K_D_), the affinity of NCK/BAD (K_D_ = 4.81×10^–8^ M) was observed to be higher than NPB/BAD (K_D_ = 3.09×10^–5^ M) (Fig. 2d, Supplementary Fig. 6A). Additionally, the pharmacological inhibition of BAD phosphorylation by NCK or NPB in TNBC cells was evaluated. By western blot analysis, starting at 0.1 μM, NCK inhibited BAD phosphorylation at Ser99, as demonstrated by a decreased pBADSer99/BAD ratio in MDA-MB-231 and BT549 cells. In contrast, compared to DMSO, NPB significantly inhibited pBADSer99/BAD protein levels at 1 μM and 10 μM in both TNBC cell lines (Fig. 2e, Supplementary Fig. 6B). Similar to NPB, NCK did not alter the levels of pBADSer75/BAD and pBADSer118/BAD (Fig. 2e, Supplementary Fig. 6B). Furthermore, the comparative potencies of NPB and NCK were evaluated in eight TNBC cell lines using total cell number assay in 2D culture and cell viability in 3D culture (Supplementary Fig. 6C, D). NCK was more potent than NPB in reducing 2D and 3D cell viability of all TNBC cell lines (Fig. 2f, Supplementary Fig. 6D). Specifically, NCK (IC_50_ = 1.015 μM in MDA-MB-231 and 1.704 μM in BT549) demonstrated a more potent effect than NPB (IC_50_ = 2.895 μM in MDA-MB-231 and 3.886 μM in BT549) in reducing the viability of TNBC cells ( ~ 3 fold in MDA-MB-231 and ~2 fold in BT549) in 2D culture. In 3D Matrigel, NCK demonstrated ~6 fold IC_50_ difference (NCK IC_50_ = 0.239 μM and NPB IC_50_ = 1.374 μM) in MDA-MB-231 cells and ~8 fold IC_50_ difference in viability (NCK IC_50_ = 0.309 μM and NPB IC_50_ = 2.584 μM) in BT549 cells (Fig. 2f). These results showed that NCK exhibits a more potent effect than NPB in reducing pBADSer99 and cell viability in TNBC cell lines in vitro and ex vivo.

### siRNA-mediated depletion of BAD expression hinders the effect of NCK

To confirm the functional specificity of NCK to BAD, the effect of NCK treatment after siRNA-mediated depletion of BAD expression was examined in MDA-MB-231 cells. Western blot analysis demonstrated that the transient transfection of MDA-MB-231 cells with siRNA-BAD decreased levels of pBADSer99 and BAD expression compared to cells transfected with scrambled oligo (Fig. 2g). Consistent with previous findings^32–34^, no significant changes in cell viability nor CASPASE 3/7 activity were observed upon transfection of siRNA directed to the BAD transcript (Fig. 2g–i). NCK increased CASPASE 3/7 activity and decreased cell viability of MDA-MB-231 cells compared to the vehicle-treated cells. However, siRNA-mediated depletion of BAD expression abolished the effect of NCK on cell viability and CASPASE 3/7 activity (Fig. 2g–i), similar to that observed previously with NPB^13^.

### Pharmacokinetics of NCK

The pharmacokinetics of NCK were determined via intravenous (IV) and oral administration in Sprague-Dawley (SD) rats (Supplementary Fig. 7). Following a single 1 mg/kg IV dose, NCK showed a multiexponential disposition with high clearance of 58.3 mL/min·kg and a high volume of distribution at steady state (Vss) of 5.93 L/kg with a t_1/2_ = 3.35 h. Following a single oral dose of 10 mg/kg, NCK showed rapid absorption followed by a multiexponential disposition with t_max_ = 0.33 h, C_max_ = 346 ng/mL, AUC_inf_ = 1072 h·ng/ml, t_1/2_ = 2.68 h and a moderate bioavailability of 37.2%, which is higher than the previously reported NPB (12.4%)^13^.

### NCK enhances apoptosis and impedes cell-cycle progression in TNBC cells

**Fig. 3:**
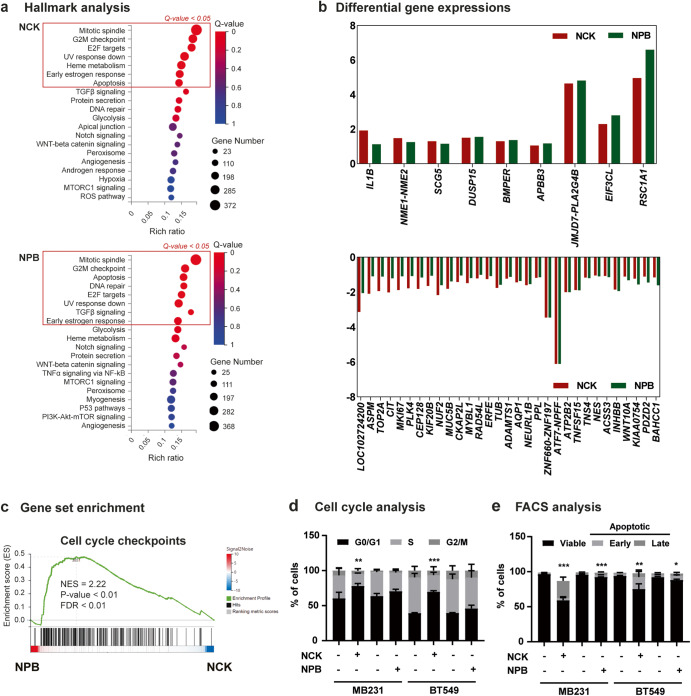
NCK enhances apoptosis and impedes cell-cycle progression in TNBC cells. **a** Hallmark analysis of the differential expressed genes (DEGs). Advanced bubble chart shows enrichment of hallmarks by DEGs commonly affected by NCK or NPB treatment. **b** Bar charts depicting DEGs commonly upregulated and downregulated by NCK or NPB treatment. **c** GSEA analyses of gene sets for cell cycle checkpoints. NES, normalized enrichment score. FDR, false discovery rate. Positive and negative NES indicate lower and higher expression in NCK when compared to NPB respectively. **d** Flow cytometry analysis of PI staining of cell cycle state of MDA-MB-231 and BT549 cells measured after treatment with 5 μM NCK or NPB using flow cytometry analysis as described in materials and methods. Data represent mean ± SD (*n* = 3). *P < 0.05, **P < 0.01, and ***P < 0.001. **e** Flow cytometry analysis of Annexin-V and propidium iodide (PI) staining of apoptotic cell death of MDA-MB-231 and BT549 cells measured after treatment with 5 μM NCK or NPB using flow cytometry analysis as described in materials and methods. Data represent means ± SD (*n* = 3). *P < 0.05, **P < 0.01, and ***P < 0.001.

To delineate the biological processes commonly and differentially affected by NCK and NPB, RNA sequencing was performed whereby MDA-MB-231 cells treated with the two pharmacological inhibitors of pBADSer99, NCK or NPB, were analyzed. Hallmark analysis of the differentially expressed genes (DEGs) demonstrated that mitotic spindle, G2M checkpoint, apoptosis, UV response and early estrogen response were commonly affected after either NCK or NPB treatment (Fig. 3a) with 9 DEGs that were commonly upregulated and 31 that were downregulated after the treatment (Fig. 3b). In 6 of 9 DEGs upregulated, the magnitude of gene change was higher in the NPB-treated cells compared to NCK-treated cells; whereas in 19 of 31 DEGs, the magnitude of gene changes with NCK treatment was higher than that of NPB. Furthermore, gene set enrichment analysis (GSEA) demonstrated that MDA-MB-231 cells treated with NCK and NPB showed significant difference in enrichment of gene sets associated with “cell cycle checkpoints” but not with “apoptosis” (Fig. 3c, Supplementary Fig. 8A).This is consistent with the gene ontology (GO) annotations in the biological process that the cells treated with NCK, but not NPB, were annotated to categories of cell cycle, cell division and chromosome segregation (Supplementary Fig. 8B, C). In a panel of cell cycle related genes significantly affected by NCK treatment, the most downregulated gene was CDC20 (cell division cycle 20 homologue), a gene responsible for activating anaphase promoting complex (APC) for anaphase entry (Supplementary Fig. 8D). Given that the transcription of cell cycle related genes was affected by the treatment with NCK, cell cycle analysis by flow cytometry was performed following inhibition of pBADSer99 by NCK or NPB (Fig. 3d, Supplementary Fig. 9A). A 15.68% decrease in S-phase and a 17.75% increase in G1-phase was observed in NCK-treated MDA-MB-231 cells, which is consistent with a G0/G1 arrest. Similarly, treatment with NCK also resulted in a 27.12% decrease in S-phase and a 30.43% increase in G1-phase in NCK-treated BT549 cells. However, NPB treatment did not significantly result in cell cycle arrest in MDA-MB-231 and BT549 cells (Fig. 3d, Supplementary Fig. 9A). Subsequently, the effect of NCK or NPB in promoting apoptotic cell death in both MDA-MB-231 and BT549 cells was assessed using the Annexin V-propidium iodide (PI) assay. Consistently observed in both TNBC cell lines, NCK demonstrated a more potent effect than NPB in inducing apoptosis (early: PI − , FITC−Annexin V + ; late: PI + , FITC−Annexin V + ) (Fig. 3e, Supplementary Fig. 9B). Thus, NCK enhances apoptosis and impedes cell-cycle progression, more potently than NPB, in TNBC cells.

### Toxicity and in vivo efficacy of NCK

**Fig. 4:**
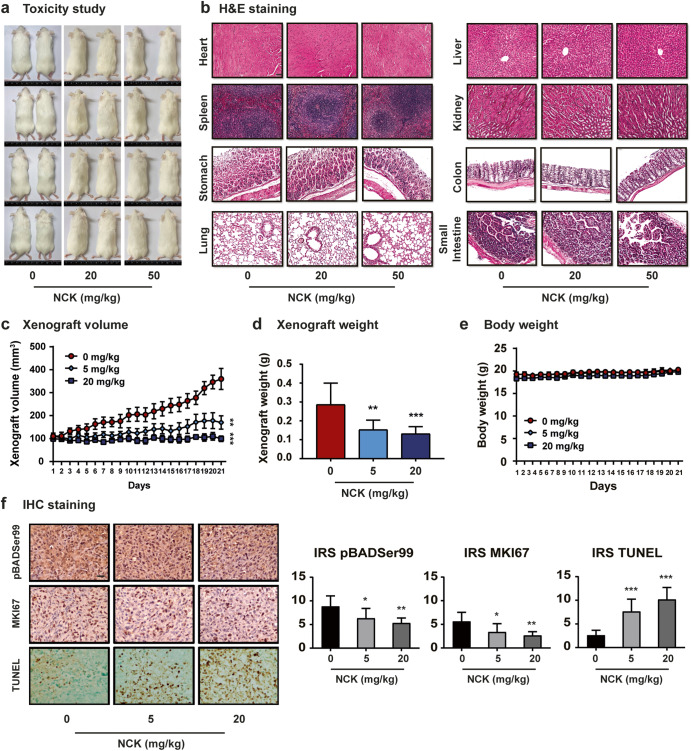
NCK suppresses xenograft growth with no toxicity up to 50 mg/kg. **a** The morphology of mice receiving 0, 20 or 50 mg/kg NCK for 14 days. **b** Morphology of the internal organs was examined using H&E staining. Scale bar, 50 µm. **c** Xenograft volume (mm^3^) was measured every day and calculated by using the formula: 0.52 × length × [width]^2^. **d** Xenograft weight of each treatment group in all animals that were sacrificed after 21 days of treatment. **e** Animal weights of each treatment group are indicated. Animal weight was monitored every day. **f** Histological analyses and IRS scoring of pBAD at Ser99, BAD, MKI67, and TUNEL staining. The IRS scoring method is described in materials & methods. Scale bar, 20 µm. All data represent means ± SD (*n* = 8). *P < 0.05, **P < 0.01, and ***P < 0.001.

To evaluate the tolerability of NCK for in vivo use, a toxicity study was carried out on mice at doses of 20 and 50 mg/kg NCK by intraperitoneal (i.p.) injection. 8 week old female Institute of Cancer Research (ICR) mice were injected i.p. with vehicle or a NCK dose of 20 or 50 mg/kg body weight continuously for 14 days. After the treatment period, NCK-treated mice did not show any significant differences in appearance or behaviour (Fig. 4a), body weight (Supplementary Fig. 10A), daily food consumption (Supplementary Fig. 10B) or water intake (Supplementary Fig. 10C) compared to the vehicle-treated mice. In addition, the relative weight of the liver, heart, spleen, stomach, lung, kidney, colon and small intestine were not significantly altered in mice treated with either 20 mg/kg or 50 mg/kg NCK as compared to the vehicle-treated group (Supplementary Fig. 10D). Histological analysis of the same organs did not demonstrate obvious pathology in mice receiving NCK at 20 mg/kg or 50 mg/kg i.p. (Fig. 4b, Supplementary Fig. 10E). There were also no significant effects of NCK treatment at 20 mg/kg or 50 mg/kg on levels of various standard haematological and serum parameters as compared to the vehicle group (Supplementary Fig. 10F).

Next, the in vivo efficacy of NCK was examined in mice injected orthotopically with MDA-MB-231 cells to form xenografts. When the xenograft volume reached 100 mm^3^, mice were randomly grouped and injected i.p. with vehicle, NCK (5 mg/kg *q.d*.) or NCK (20 mg/kg *q.d*.) for 21 days. NCK treatment at both 5 mg/kg and 20 mg/kg significantly reduced xenograft volume (Fig. 4c) and weight (Fig. 4d) with no significant change in body weight (Fig. 4e) when compared to the vehicle treated group. Histological analyses of xenografts resected from mice treated with NCK showed significantly reduced pBADSer99 compared to vehicle-treated mice, accompanied by decreased MKI67 positivity and increased TUNEL scores (Fig. 4f).

### Tyrosine kinase inhibitors (TKIs) identified as the most synergistic compounds in combination with NCK to reduce MDA-MB-231 cell survival

**Fig. 5:**
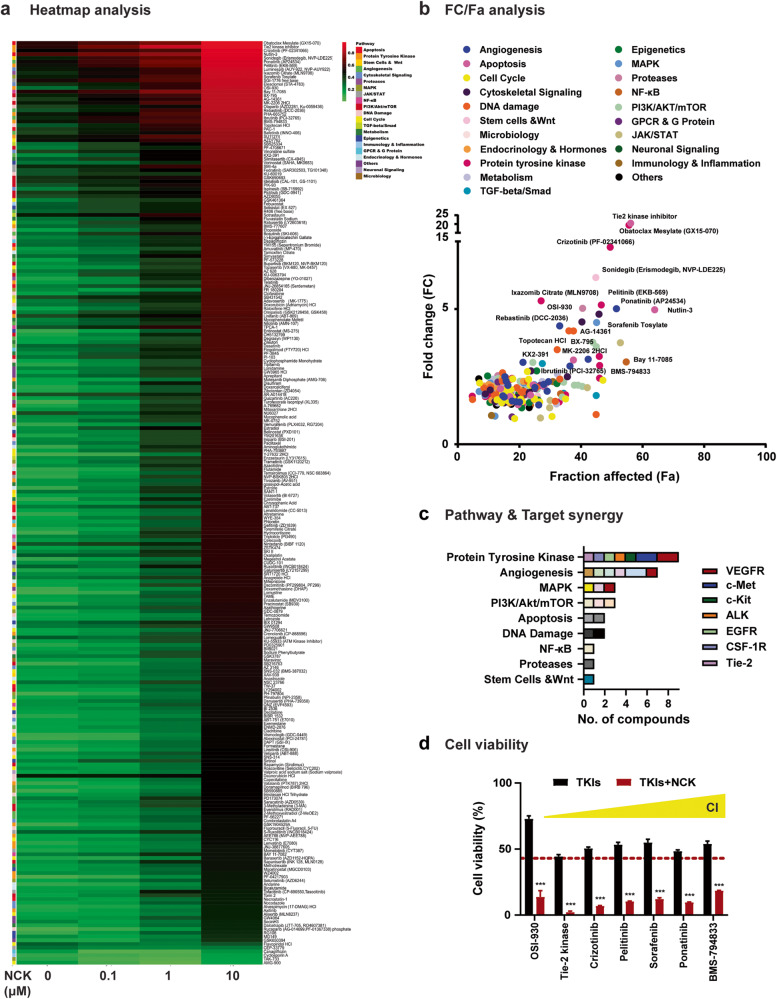
Tyrosine kinase inhibitors (TKIs) identified as the most synergistic compounds in combination with NCK to reduce MDA-MB-231 cell survival. **a** Heatmap plot depicts cell viability of MDA-MB-231 cells post-treatment as % Fraction affected (Fa) (Scale: Green to Red). Fa was calculated as 100 - cell viability (%). **b** Fold change (FC) of cell viability after treatments (compound X versus compound X + 10 μM NCK) and Fa of compound X alone were plotted. Compounds were marked in different colors, each representing the pathway targeted by the compound. Compounds with average CI < 1 when co-treated with NCK are shown. CI was calculated using the bliss independence method (CI= (E_A_ + E_B_-E_A_E_B_)/E_AB_), where CI < 1 denotes synergy. **c** Top pathways and targets synergizing with NCK are plotted. **d** Cell viability of MDA-MB-231 cells after treatment with the highly ranked synergistic TKIs (at their respective IC_25_) with/without 10 μM NCK (from drug screening assay). Data represent means ± SD. *P < 0.05, **P < 0.01, and ***P < 0.001.

The Cambridge anti-cancer compound library was screened in combination with NCK in a TNBC cell line (MDA-MB-231) (Supplementary Fig. 11). Screening of 247 anti-cancer compounds, targeting a wide range of pathways including angiogenesis, apoptosis, PI3K/AKT/mTOR, MAPK, protein tyrosine kinases and metabolism, was performed on MDA-MB-231 cells to identify NCK-based synergistic combinations in TNBC cells (Fig. 5a). Among the 21 generalized drug groupings, “protein tyrosine kinase” was the grouping with the most compounds synergizing with NCK, followed by “angiogenesis” and the “MAPK pathway”. Among the protein tyrosine kinases, VEGFR and c-MET exhibited the highest target synergy with NCK. It is also noteworthy that some of the compounds targeting VEGFR were categorized under “angiogenesis” and “MAPK pathway” due to multi-target inhibition (polypharmacology) (Fig. 5b, c). Among the synergistic combinations of NCK with tyrosine kinases inhibitors (TKIs) identified, OSI-930 (dual VEGFR2 and c-KIT inhibitor) and Crizotinib (dual c-MET and ALK inhibitor) were selected for further in-depth investigation in combination with NCK in TNBC cell lines (Fig. 5d). These two compounds were chosen because of their low CI (high synergy) and targets on VEGFR and c-MET respectively. Notably, the inhibitors of RTKs usually inhibit multiple other kinases (Supplementary Fig. 12A). Therefore, the synergistic effect of TKIs with NCK may potentially be exerted through multi-target inhibition. Detailed FDA/ clinical trial related information of OSI-930 and Crizotinib are listed in Supplementary Fig. 12B.

### NCK synergizes with TKIs to reduce cell viability

**Fig. 6:**
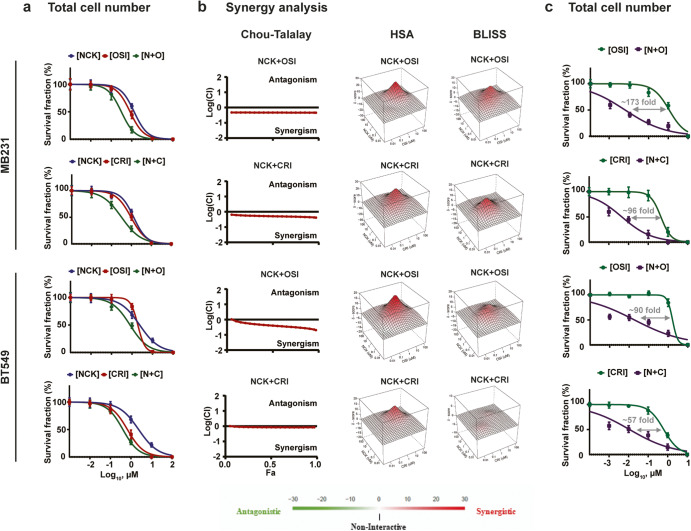
NCK synergizes with OSI-930 or Crizotinib to reduce TNBC cell survival. **a** The survival fraction of NCK, OSI-930 (OSI) and Crizotinib (CRI) or combination treatments were evaluated with total cell number assay (*n* = 3). **b** CI was measured with Chou-Talalay, where CI < 1 denotes synergy, CI = 1 denotes additivity, CI > 1 denotes antagonism. Synergy score was measured with HSA and bliss synergy analysis (www.synergyfinder.com), where CI > 0 denotes synergy, CI < 0 denotes antagonism. **c** Dose-response analysis of the shift in IC_50_ of OSI-930 (OSI) and Crizotinib (CRI) in TNBC cells after co-treatment with NCK (2 μM) was evaluated with total cell number assay. Fold difference was calculated. Data represent means ± SD (*n* = 3).

In order to further verify the synergistic effect of the pBADSer99 inhibitor NCK and TKIs obtained from the anti-cancer compound library screen, the effect of drug combinations (NCK-OSI-930 or NCK-Crizotinib) at 5 concentrations (0.01-100 μM) were evaluated by using cell viability assays in MDA-MB-231 and BT549 cells (Fig. 6a). In both cell lines, the combinatorial treatments exhibited synergistic effects, as demonstrated by the Chou-Talalay method, highest single agent (HSA) and bliss synergy analysis (Fig. 6b). Subsequently, the effect of NCK on the IC_50_ of the two TKIs were determined. The potency and synergy of the NCK-OSI-930 and NCK-Crizotinib combinations were reflected by the marked reduction in IC_50_ values of both TKIs in both TNBC cell lines (Fig. 6c). NCK significantly decreased the IC_50_ of OSI-930 and Crizotinib by ~173 fold and ~96 fold in MDA-MB-231 cells respectively, and similarly decreased the TKI IC_50_ by ~90 fold and ~57 fold in BT549 cells, respectively.

### NCK synergizes with TKIs to stimulate intrinsic apoptosis

**Fig. 7:**
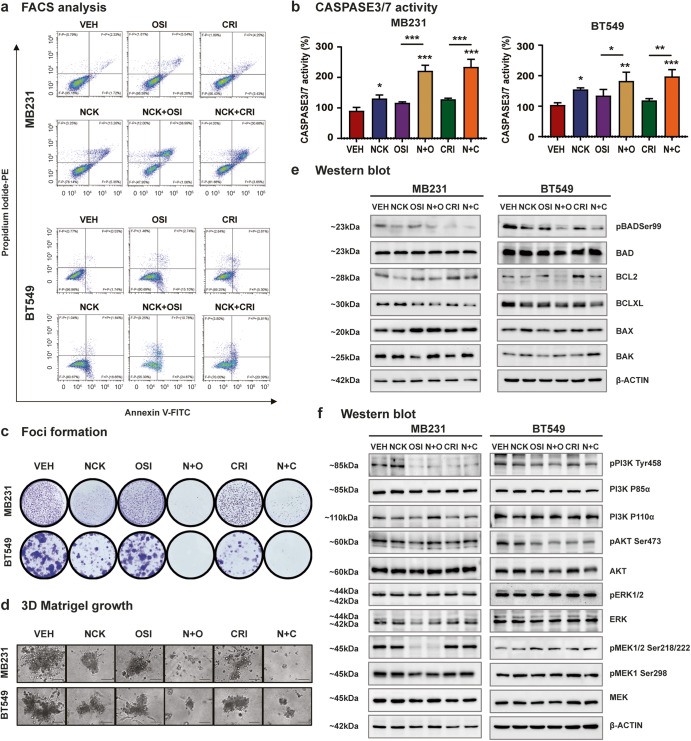
NCK synergizes with OSI-930 or Crizotinib to stimulate caspase-mediated apoptotic cell death and reduce cell survival in vitro and ex vivo. **a** Representative flow cytometry plots using Annexin V FITC/PI staining for apoptotic cell death of MDA-MB-231 and BT549 cells measured after treatment with 5 μM NCK, 5 μM OSI-930 (OSI), 5 μM Crizotinib (CRI) or combinations using flow cytometry analysis at 72 hours as described in materials and methods (*n* = 3). **b** CASPASE 3/7 activities were evaluated in MDA-MB-231 and BT549 cells using the Biovision Caspase 3/7 DEVD Assay Kit after treatment with 5 μM NCK, 5 μM OSI-930 (OSI), 5 μM Crizotinib (CRI) or combinations. Data represent means ± SD (*n* = 3). *P < 0.05, **P < 0.01, and ***P < 0.001. **c** Crystal violet staining of foci in colonies generated by MDA-MB-231 cells and BT549 cells after exposure to 5 μM NCK, 5 μM OSI-930 (OSI), 5 μM Crizotinib (CRI) or combinations. **d** Representative images of MDA-MB-231 cells and BT549 cells cultured in 3D Matrigel after exposure to 5 μM NCK, 5 μM OSI-930 (OSI), 5 μM Crizotinib (CRI) or combinations. Scale bar, 100μm. **e** Western blot analysis was used to assess the level of various apoptotic proteins in TNBC cells after treatment with 1 μM NCK, 1 μM OSI-930 (OSI), 1 μM Crizotinib (CRI) or combinations. β-ACTIN was used as input control for cell lysate. The sizes of detected bands in kDa are shown on the left. **f** Western blot analysis was used to assess the expression/phosphorylation of various proteins of the PI3K/AKT and MAPK pathways after treatment with 1 μM NCK, 1 μM OSI-930 (OSI), 1 μM Crizotinib (CRI) or combinations. β-ACTIN was used as input control for cell lysate. The sizes of detected bands in kDa are shown on the left.

Flow cytometry results demonstrated that combinatorial treatment of NCK and OSI-930 or Crizotinib significantly promoted apoptotic cell death in both TNBC cell lines compared to NCK, OSI-930 or Crizotinib single treatment (Fig. 7a, Supplementary Fig. 13A). The combinatorial treatment of NCK and OSI-930 promoted apoptosis in a synergistic manner in both TNBC cell lines. The combined NCK-Crizotinib treatment synergistically and additively induced apoptosis in MDA-MB-231 and BT549 cells, respectively. Consistently, co-treatment of NCK with OSI-930 or Crizotinib in MDA-MB-231 cells and NCK with Crizotinib in BT549 cells synergistically increased CASPASE 3/7 activity; whereas co-treatment of NCK-OSI-930 increased CASPASE 3/7 activity in BT549 cells in an additive manner (Fig. 7b). Additionally, the effect of treatments on 2D foci formation and 3D colony growth in MDA-MB-231 and BT549 cells were evaluated. The treatment with NCK, OSI-930 or Crizotinib alone significantly attenuated the capacity for foci formation. Combined treatment of NCK with OSI-930 or Crizotinib elicited higher inhibition than single treatments on foci formation capacity in both cell lines (Fig. 7c, Supplementary Fig. 13B). For 3D ex vivo assays, similarly, NCK and single TKI treatment reduced cell growth in 3D Matrigel. Combined treatments of NCK and OSI-930 or NCK and Crizotinib markedly reduced ex vivo growth in 3D culture of both TNBC cell lines (Fig. 7d, Supplementary Fig. 13C). Western blot results demonstrated that NCK, OSI-930 or Crizotinib significantly decreased the level of pBADSer99/BAD in both MDA-MB-231 and BT549 cells. In MDA-MB-231 cells, combinatorial treatment of NCK and OSI-930 further significantly reduced pBADSer99/BAD levels compared to OSI-930, whereas co-treatment of NCK and OSI-930 or NCK and Crizotinib significantly reduced pBADSer99/BAD levels in BT549 cells when compared to OSI-930 or Crizotinib alone (Fig. 7e, Supplementary Fig. 14A). For the anti- and pro-apoptotic markers, NCK treatment significantly decreased the expression of BCL-2 in MDA-MB-231 and BT549, and increased the expression levels of BAX and BAK in BT549 cells (Fig. 7e, Supplementary Fig. 14A). When examining the ratio of anti-apoptotic and pro-apoptotic markers, NCK reduced the ratio of BCL-2/BAX and BCL-2/BAK in MDA-MB-231 cells, and reduced BCL-2/BAX, BCL-2/BAK, BCL-XL/BAX and BCL-XL/BAK in BT549 cells. The combinatorial treatment of NCK and OSI-930 significantly further reduced the BCL-2/BAK and BCL-XL/BAK ratios compared to OSI-930 treatment in MDA-MB-231 cells, whereas in BT549 cells, the combined treatment of NCK and OSI-930 reduced BCL-2/BAX, BCL-2/BAK, BCL-XL/BAX and BCL-XL/BAK ratios as compared to OSI-930 alone. For the combined NCK-Crizotinib treatment, the ratio of BCL-2/BAK, BCL-XL/BAX and BCL-XL /BAK were significantly reduced as compared to Crizotinib-treated cells in MDA-MB-231 cells. In BT549 cells, when compared to Crizotinib single treatment, BCL-2/BAX, BCL-2/BAK, BCL-XL/BAX and BCL-XL/BAK ratios were significantly decreased with combined NCK-Crizotinib treatment (Fig. 7e, Supplementary Fig. 14A).

Next, western blot analyses were performed to determine potential alteration in expression or activity of the target proteins along the MAPK and PI3K/AKT pathways. Similar to NPB reported earlier in estrogen receptor (ER) + BC cell lines^13^, western blot results showed that single treatment with NCK alone did not affect the levels of phosphorylated nor total protein of components of MAPK or PI3K/AKT pathways in TNBC cells (Fig. 7f, Supplementary Fig. 14B). In contrast, it was observed that OSI-930 or Crizotinib significantly inhibited the PI3K/AKT pathway in MDA-MB-231 cells by reducing the levels of p-PI3K (Tyr458)/PI3K and p-AKT (Ser473)/AKT. Additionally, OSI-930 treatment reduced the level of p-MEK1/2 (Ser218/222)/MEK and increased the level of p-ERK/ERK by reducing the expression of ERK in MDA-MB-231 cells. In BT549 cells, OSI-930 significantly reduced the level of p-AKT (Ser473)/AKT but increased the levels of p-MEK1/2 (Ser218/222)/MEK, whereas Crizotinib significantly reduced the level of p-AKT (Ser473)/AKT (Fig. 7f, Supplementary Fig. 14B).

### NCK synergizes with TKIs to suppress human MDA-MB-231 xenograft and mouse 4T1 homograft growth

**Fig. 8:**
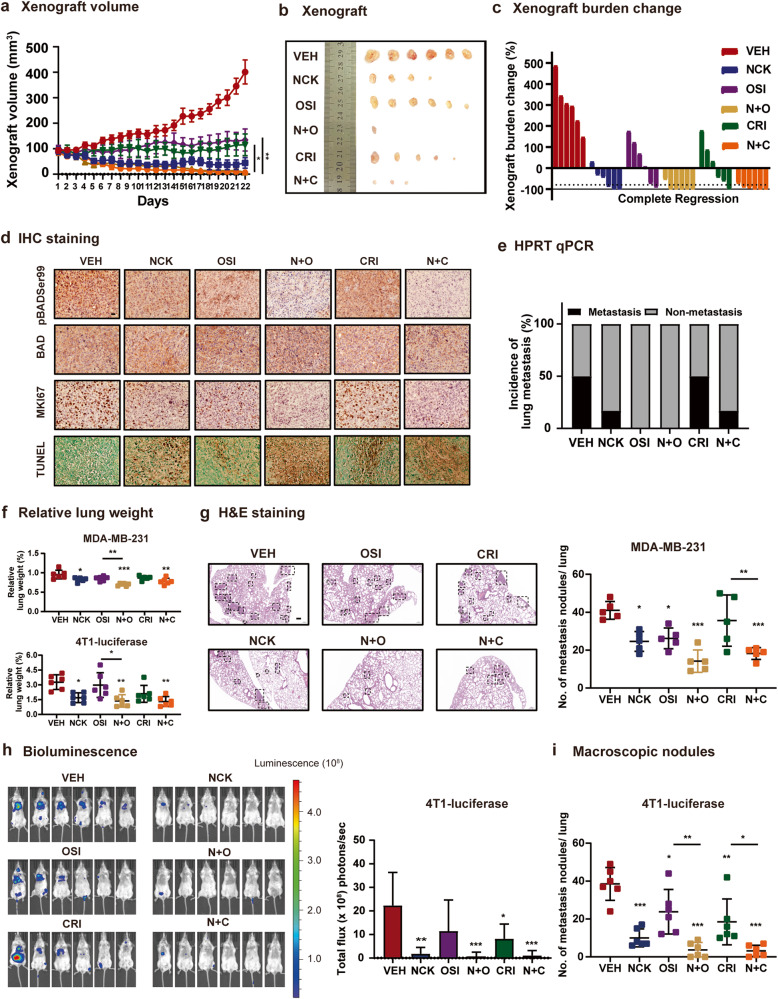
NCK synergizes with TKIs to suppress TNBC xenograft growth and lung metastasis. **a** MDA-MB-231 xenograft volume (mm^3^) was measured every day and calculated by using the formula: 0.52 × length × [width]^2^. **b** Resected MDA-MB-231 xenografts of each treatment group after sacrifice at the end of 21^st^ day. **c** Xenograft burden change of MDA-MB-231 xenografts for each treatment group after sacrifice at the end of 21^st^ day. **d** IHC images of pBAD at Ser99, BAD, MKI67 and TUNEL staining in xenografts. Scale bar, 20 µm. **e** Metastases were detected using human *hypoxanthine-guanine phosphoribosyltransferase* (*hHPRT*) mRNA per lung of BALB/c-nude mice orthotopically implanted with MDA-MB-231 cells by using qPCR. Mouse *glyceraldehyde 3-phosphate dehydrogenase* (*mgapdh*) was used as an internal control. Lung sample with CT value < 35 was regarded as metastatic and ≥ 35 was regarded as non-metastatic. **f** Relative lung weight (to body weight) of mice intravenously injected with MDA-MB-231 or 4T1-luciferase cells after treatment with vehicle, NCK, OSI-930 (OSI), Crizotinib (CRI) or combinations. **g** H&E staining and quantitative measurement of micro-metastatic nodules in lungs of MDA-MB-231 cell generated metastasis model. Scale bar: 200 μm. **h** Bioluminescence images and quantification of total flux in the lungs of 4T1-luciferase cell generated metastasis model for each treatment group at the end of the experiment. **i** Quantitative measurement of macro-metastatic nodules in the 4T1-luciferase metastasized lung of mice in each treatment group at the end of the experiment. All data represent means ± SD (*n* = 6). *P < 0.05, **P < 0.01, and ***P < 0.001.

Given the synergism observed in vitro, it was reasoned that combined inhibition of pBADSer99 and OSI-930 or Crizotinib may also lead to synergism in vivo. The effect of NCK in combination with OSI-930 or Crizotinib against MDA-MB-231 cell generated xenograft and 4T1-luciferase cell generated homograft (syngeneic) growth in vivo were examined. When the xenografts/homografts became palpable (approximately 100 mm^3^ in size), the mice were randomized and injected with vehicle, NCK (20 mg/kg *q.d*.), OSI-930 (20 mg/kg *q.d*.), Crizotinib (50 mg/kg *b.i.w*.), or the combination of NCK with OSI-930 or Crizotinib for 21/15 days (depending on humane endpoint). In both MDA-MB-231 xenograft and 4T1-luciferase homograft models, mice from all treated groups exhibited significant decreases in xenograft/homograft volume (Fig. 8a, Supplementary Fig. 16A) and weight (Supplementary Fig. 15A, Supplementary Fig. 16B) compared to the vehicle treated group. Additionally, mice receiving combined treatment of NCK and OSI-930 or NCK and Crizotinib demonstrated significant reductions in xenograft/homograft volume and weight compared to OSI-930 or Crizotinib single treatment groups respectively (Fig. 8a, Supplementary Fig. 15A, Supplementary Fig. 16A, B). In MDA-MB-231 xenografts, treatment with NCK alone resulted in the complete regression of 2/6 (33.33%) of the xenografts and treatment with Crizotinib alone resulted in complete regression of 1/6 (16.67%) xenografts; whereas combination treatments employing NCK with OSI-930 or NCK with Crizotinib resulted in complete regression of 5/6 (83.33%) and 3/6 (50.00%) xenografts respectively (Fig. 8b, c). All mice in the vehicle group exhibited progressive disease (xenograft volume >100 mm^3^) at the end of day 21 (Table 2). In 4T1-luciferase homografts, the results obtained from bioluminescence signals (Supplementary Fig. 16C) and homograft burden change (Supplementary Fig. 16D, E) were consistent with changes in homograft volume and weight.

**Table 2:** Xenograft regression.

% Regression	VEH	NCK	OSI	N + O	CRI	N + C
Complete Response (100%)	0	33	0	83	17	50
Partial Response (>50%)	0	17	33	17	17	50
Disease Progression	100	50	67	0	66	0

Statistics of regression (%) of xenograft in mice receiving NCK (NCK), Crizotinib (CRI) and OSI-930 (OSI) or combinations group.

Consistent with the previous xenograft (Fig. 4f), NCK treatment reduced the level of pBADSer99 in the resected MDA-MB-231 xenograft compared to vehicle-treated mice, whereas BAD protein was not significantly different. Mice treated with NCK exhibited significantly reduced MKI67 positivity and increased TUNEL scores in the MDA-MB-231 xenograft compared to mice treated with vehicle. When compared to MDA-MB-231 xenografts resected from mice receiving OSI-930 or Crizotinib, reduction in the level of pBADSer99 by combination treatments were accompanied by decreased MKI67 positivity. Additionally, mice receiving combined treatment of NCK and OSI-930 exhibited significantly increased TUNEL staining when compared to MDA-MB-231 xenografts of OSI-930 treated mice (Fig. 8d, Supplementary Fig. 15B). Similar effects on the pBADS99/BAD ratio, MKI67 positivity and TUNEL scores were observed in 4T1-luciferase homografts after the respective treatments (Supplementary Fig. 16F). In terms of metastasis, no macroscopic colony was visible in lung samples of MDA-MD-231 cell engrafted mice from all treatment groups, yet IHC staining of human HPRT demonstrated that cells of human origin were detectable in lung sections of MDA-MB-231-engrafted mice receiving vehicle or Crizotinib treatment but not in the lung sections of mice treated with NCK, OSI-930, NCK-OSI-930 or NCK-Crizotinib (Supplementary Fig. 15C). The results were further verified by the determination of human *HPRT* (*hHPRT*) gene expression in lung relative to mouse *gapdh* (*mgapdh*) mRNA using real-time qPCR to identify the burden of lung metastases in each treatment group (Fig. 8e)^35,36^. When compared to vehicle-treated group, in which 3 out of 6 mice were positive for *hHPRT* mRNA, the treatment with NCK reduced lung metastasis incidence to 1/6 mice as determined by detectable lung *hHPRT* mRNA. Additionally, lung metastasis incidence was significantly reduced by treatment with OSI-930 in which none of the mice had lung metastasis as detected by qPCR in both OSI-930 and NCK-OSI-930 groups. Even though the incidence of lung metastasis in mice treated with Crizotinib was the same as vehicle (3/6 mice with detectable *hHPRT*), only 1 out of 6 mice receiving the combinatorial treatment of NCK and Crizotinib had detectable metastatic human cells in lung as determined by *hHPRT* mRNA expression (Fig. 8e).

All mice tolerated the treatment regimens with no adverse impact on weight (Supplementary Fig. 15D, Supplementary Fig. 16A) or other noticeable toxic effects as determined by serum biochemical parameters (Supplementary Fig. 15E), suggesting that the drug combinations were well tolerated in vivo. Organ weights were not significantly different between the treatment groups except for spleen, which was significantly higher in the vehicle-treated mice (0.20 ± 0.06 g) when compared to mice receiving single (NCK, OSI-930 or Crizotinib) or double (NCK-OSI-930 or NCK-Crizotinib) treatments (0.09 ± 0.03 to 0.13 ± 0.05 g) in MDA-MB-231 xenografts (Supplementary Fig. 15F); and was significantly higher in the vehicle-treated mice (0.62 ± 0.11 g) when compared to mice receiving NCK or combination treatments (0.24 ± 0.05 to 0.42 ± 0.09 g) in 4T1-luciferase homografts (Supplementary Fig. 16G). This phenomenon of increased spleen weight has been previously observed in TNBC xenografts, which is associated with myeloid cell recruitment to the spleen after MDA-MB-231^37^ or 4T1^38^ cell inoculation.

### NCK in combination with TKIs suppresses TNBC lung metastasis

Since metastasis is the primary contributor to mortality and poor prognosis for TNBC patients, the therapeutic potential of NCK, TKIs or combined NCK-TKI treatment on TNBC metastasis was further evaluated with an experimental lung metastasis model^39^. The lung metastases were established by intravenous injection of MDA-MB-231 or 4T1-luciferase cells into the tail vein of BALB/c-nude or BALB/c mice respectively and were similarly given vehicle, NCK (20 mg/kg *q.d*.), OSI-930 (20 mg/kg *q.d*.), Crizotinib (50 mg/kg *b.i.w*.) or the combination treatments. In the MDA-MB-231 cell generated metastasis model, although no macro-metastases were observed, reduced relative lung weight was observed in the mice receiving NCK (0.17 ± 0.01 g), NCK-OSI-930 (0.14 ± 0.01 g) or NCK-CRI (0.15 ± 0.01 g) compared to vehicle-treated mice (0.19 ± 0.02 g) (Fig. 8f). NCK-OSI-930 combination treatment (0.14 ± 0.01 g) further significantly reduced the relative lung weight as compared to OSI-930 treatment (0.16 ± 0.01 g) (Fig. 8f). Subsequent histological analysis (H&E) for micro-metastatic nodules in the lung supported reduced lung TNBC cell colonization in mice receiving treatment compared to vehicle-treated mice (Fig. 8g). The quantitative measurement of micro-metastatic nodules by H&E staining demonstrated that treatment with NCK, OSI-930, NCK-OSI-930 or NCK-Crizotinib significantly reduced lung colonization compared to vehicle treatment. Furthermore, mice receiving NCK-Crizotinib treatment exhibited a significantly lower number of micro-metastatic nodules in lung compared to Crizotinib-treated mice (Fig. 8g). In the 4T1-luciferase cell generated metastasis model, mice receiving NCK or combination treatments (0.22 ± 0.08 to 0.29 ± 0.07 g) exhibited significantly lower relative lung weight as compared to mice receiving vehicle treatment (0.51 ± 0.10 g) (Fig. 8f). NCK-OSI-930 treatment (0.23 ± 0.08 g) further reduced the relative lung weight as compared to OSI-930 treatment (0.46 ± 0.18 g) significantly (Fig. 8f). The metastatic burden of mice intravenously injected with 4T1-luciferase cells was further determined by bioluminescent imaging (Fig. 8h) and counting of macroscopic nodules (Fig. 8i, Supplementary Fig. 17A) in lung. Whereas mice in the control group exhibited abundant lung metastasis as demonstrated by bioluminescent signals, NCK, CRI or combination treatments (NCK-OSI-930 and NCK-CRI) significantly abrogated lung metastases derived from tail vein injected 4T1-luciferase cells (Fig. 8h). Consistent results were obtained by counting of macro-metastatic nodules in the lungs of the same treatment groups (Fig. 8i, Supplementary Fig. 17A). Additionally, NCK-OSI-930 and NCK-CRI combinations significantly reduced lung metastatic colonization of 4T1-luciferase cells compared to single treatment with OSI-930 and CRI, respectively, as demonstrated similarly by counting of macro-metastatic nodules (Fig. 8i). Similar effects of NCK, TKIs or combination treatments on the level of pBADSer99 and on BAD expression were observed by IHC in both MDA-MB-231 and 4T1-luciferase cell generated metastasis models (Supplementary Fig. 17B, Supplementary Fig. 18A). No significant difference was observed in host animal body weight (Supplementary Fig. 17C, Supplementary Fig. 18B) and the weight of other vital organs (Supplementary Fig. 17D, Supplementary Fig. 18C) between the treatment groups, indicative of the tolerability of the treatments.

### NCK in combination with TKIs suppresses patient-derived xenograft (PDX) growth and extends the survival of the PDX-engrafted mice

**Fig. 9:**
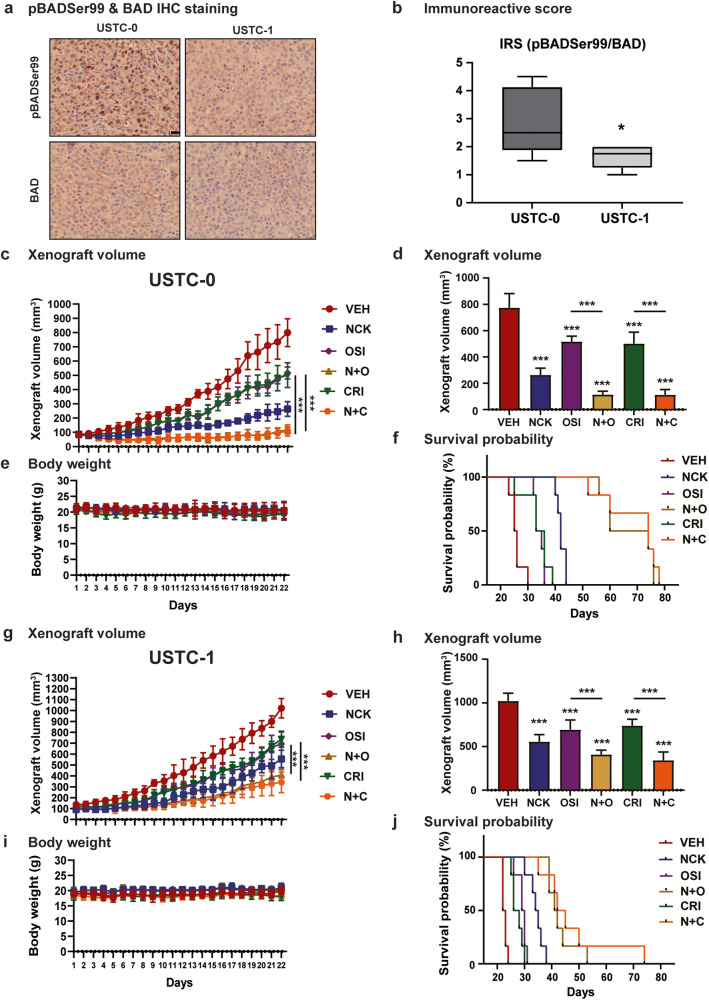
NCK in combination with TKIs suppresses patient-derived xenograft (PDX) growth and extends the survival of the PDX-engrafted mice. **a** Representative IHC images of pBADSer99 level and BAD expression in USTC-0 and USTC-1. Scale bar, 20 µm. **b** IRS score of pBADSer99/BAD in USTC-0 and USTC-1. The immunoreactive score (IRS) 0 to 4 was categorized as negative and IRS 5 to 12 as positive. **c** Xenograft volume (mm^3^) of USTC-0 was measured every day and calculated by using the formula: 0.52 × length × [width]^2^. **d** Mean USTC-0 xenograft volume of each treatment group at the end of 21^st^ day. **e** Animal weight (mean ± SD) of USTC-0 of each treatment group (*n* = 6). **f** Kaplan–Meier survival curves of USTC-0 treated with NCK (NCK), Crizotinib (CRI), OSI-930 (OSI) or combinations. **g** Xenograft volume (mm^3^) of USTC-1 was measured every day and calculated by using the formula: 0.52 × length × [width]^2^. **h** Mean USTC-1 xenograft volume of each treatment group at the end of 21^st^ day. **i** Animal weight (mean ± SD) of USTC-1 of each treatment group (*n* = 6). **j** Kaplan–Meier survival curves of USTC-1 treated with NCK (NCK), Crizotinib (CRI), OSI-930 (OSI) or combinations. All data represent means ± SD (*n* = 6). *P < 0.05, **P < 0.01, and ***P < 0.001.

The effect of drug treatments on two patient-derived xenograft (PDX) models of TNBC, USTC-0 and USTC-1 were next examined. The use of the USTC-1 PDX was previously reported^40^. USTC-0 and USTC-1 exhibit differential phosphorylation of BADSer99 (expressed as pBADSer99/BAD), as assessed by IHC (Fig. 9a, b). In detail, USTC-0 exhibited a higher pBADSer99 level (Supplementary Fig. 19A, B) and pBADSer99/BAD ratio (Fig. 9b) than USTC-1. Similar to the MDA-MB-231 xenograft, USTC-0- or USTC-1-bearing mice were treated with NCK (20 mg/kg *q.d*.), OSI-930 (20 mg/kg *q.d*.), Crizotinib (50 mg/kg *b.i.w*.), or the combination of NCK with OSI-930 or Crizotinib for 21 days. In both USTC-0 and USTC-1-bearing mice, when compared to the vehicle treated groups, NCK, OSI-930 and Crizotinib treatment significantly reduced the xenograft volume (Fig. 9c, d, g, h). Additionally, the combinatorial treatments of NCK-OSI-930 and NCK-Crizotinib were significantly more effective in reducing the PDX volumes than treatment with either OSI-930 or Crizotinib alone. However, consistent with the lower pBADSer99 level and pBADSer99/BAD ratio in USTC-1, similar but less pronounced effects were observed when compared to mice bearing USTC-0 (*P* < 0.01) (Supplementary Fig. 19C). Treatment of USTC-1-bearing mice with NCK resulted in a 45.6% reduction in xenograft burden compared to those treated with vehicle, as compared to 66.0% reduction in mice bearing USTC-0 (*P* < 0.01). Consistently, the combination treatment of NCK and OSI-930 (*P* < 0.001) or Crizotinib (*P* < 0.05) reduced USTC-0 xenograft burden by 60.04% and 66.58% as compared to 85.33% and 85.44% in the USTC-1 xenograft. The drug combination regimens were well tolerated in mice, according to body weight (Fig. 9e, i).

Consistent with the suppression of PDX growth, NCK, OSI-930 and Crizotinib markedly extended the survival of USTC-0-bearing mice when compared to the vehicle-treated group. The combinatorial treatments of NCK-OSI-930 and NCK-Crizotinib demonstrated significant prolongation in survival (*P* < 0.001) when compared to OSI-930 or Crizotinib alone (Table 3). Specifically, the median survival days of each treatment group were vehicle (25.5), NCK (42.0), OSI-930 (34.0), Crizotinib (34.5), NCK-OSI-930 (67.0) and NCK- Crizotinib (74.0) (Fig. 9f). In mice bearing USTC-1, the median survival days of each treatment group were vehicle (22.5), NCK (34.5), OSI-930 (29.5), Crizotinib (27.0), NCK-OSI-930 (41.5) and NCK-Crizotinib (43.5) (Fig. 9j). No PDX in any group reached the humane termination endpoint during the 21-day treatment period.

**Table 3:** PDX host animal median survival.

	PDX	VEH	NCK	OSI	N + O	CRI	N + C
Median survival (Days)	USTC-0	25.5	42.0	34.0	67	34.5	74
	USTC-1	22.5	34.5	29.5	41.5	27.0	43.5

Median survival time of mice receiving NCK (NCK), Crizotinib (CRI), OSI-930 (OSI) or combinations in TNBC PDX USTC-0 or USTC-1.

## Discussion

Despite tremendous efforts, there remains no effective targeted therapy for TNBC to date. TNBC frequently harbors mutations with enhanced activation of the PI3K/AKT signaling pathway and is associated with poor prognosis^4,41,42^. Therefore, targeting kinases along the PI3K/AKT pathway may represent a rational therapeutic opportunity to treat this aggressive subtype of BC^41^. Given that BAD phosphorylation at Ser99 is also predominantly mediated by kinases along the PI3K/AKT pathway, which as stated is frequently activated in TNBC^8,12^, targeting BADSer99 phosphorylation represents a viable mechanistic based therapeutic strategy. Consistent with previous studies^12,43^, it was confirmed herein that TNBC tissues exhibit a higher pBADSer99/BAD ratio when compared to normal tissues and which is correlated with higher grade, higher MKI67 labeling and increased lymph node metastasis. Additionally, the inhibition of cell survival and migrative capacity by homology directed repair of BAD to BADS99A demonstrated that phosphorylation of BAD Serine 99 is an actionable target in TNBC. Previously, proof of concept studies using the pBADSer99 inhibitor, NPB, were reported in various cancer models including ER+BC, cisplatin-sensitive or resistant OC and PTEN-deficient EC^13,30,31^. Herein, a more potent inhibitor of pBADSer99 with enhanced oral bioavailability that significantly increased apoptotic cell death in TNBC was generated. Given that molecular docking analyses have identified that NPB may interact with other proteins in addition to BAD^13^, as is common with other “targeted” small molecule compounds^44,45^, there exists the possibility of NCK polypharmacology especially at higher concentrations. Determination of genes/gene functions specifically responsive to BADSer99 phosphorylation and determining if NCK also affects expression of these genes, or a subset of these genes, or exerts effects separately, may be of utility to determine any potential polypharmacology of NCK. This approach will, however, be complicated by the observation that both non-phosphorylated and phosphorylated BAD exert independent cellular functions^8^ and heterodimerization of BAD has been observed with multiple cellular proteins in addition to BCL-2 family members, such as hexokinase^46,47^, c-Jun^10^, p53^48^ and androgen receptor^49^. The RNA-sequencing and functional analyses contained herein do however suggest that NCK impacts both cancer cell survival and cell cycle, as might be expected^10,50–52^. Regardless of any potential polypharmacology, NCK exhibited potent effects on in vivo models of TNBC, was well tolerated and synergized with TKIs. Different to NPB, pharmacological inhibition of BADSer99 phosphorylation by NCK also markedly impacted cell cycle related gene expression and prevented cell cycle progression. A role for BAD in regulating cell cycle has been previously suggested to be exerted through inhibiting AP1-mediated CYCLIN D1 expression and S phase entry in ER+BC, leading to G0/G1 growth arrest^10^. This activity was also reported to be dependent on BAD phosphorylation at Ser75 and Ser99. However, anchorage independent growth of mouse derived cells has been reported to be dependent on Ser136 (human Ser99) and correlated to Bad binding to 14-3-3^53^. Another study has demonstrated that BAD maintains cell cycle progression in low serum conditions (as herein) although this effect was reported to be independent of BAD phosphorylation requiring heterodimerization with BCL-XL^52^. Herein, it was observed that a higher pBADSer99/BAD ratio was significantly associated with MKI67 labeling in TNBC samples indicative of a proliferative function for BADSer99 phosphorylation. Hence, the precise mechanism by which BAD and NCK perturbation of the pBADSer99/BAD ratio impacts on TNBC cell cycle progression needs further delineation. It was further observed that NCK significantly suppressed TNBC xenograft growth at 5 mg/kg and 20 mg/kg despite no significant toxicity being observed at 50 mg/kg and hence possesses a good therapeutic window. Furthermore, even though NCK is effective in combination therapies, it exhibits relatively potent single agent activity and hence could be of utility in patients with specific molecular or mutational indications, such as cancers with a high BAD phosphorylation ratio or mutations of the PI3K/AKT pathway.

In the present study, pharmacological inhibition of RTKs, specifically VEGFR and c-MET exhibited the most synergistic effects in combination with NCK in reducing cell viability of MDA-MB-231 cells. VEGFR and c-MET are two RTKs with pivotal roles in regulating cell proliferation, angiogenesis and metastasis^7,54^. Previous work has demonstrated the role of VEGFR in increasing the TNBC cancer stem cell (CSC) population and metastasis^55^ and the association of c-MET expression with poor prognosis in TNBC^56^. Among the VEGFR and c-MET inhibitors, OSI-930 and Crizotinib showed the highest synergy in combination with NCK. OSI-930 is an orally selective TKI targeting VEGFR2 (9 nM) and Kit (80 nM), which is currently being evaluated in phase 1 clinical trial^57^. Crizotinib, an ATP-competitive, small molecule TKI targeting c-MET (11 nM) and ALK (24 nM), was approved by the FDA in 2011 to treat Anaplastic Lymphoma Kinase-rearranged (ALK + ) non-small cell lung cancer (NSCLC). Therefore, through multi-kinase inhibition (polypharmacology), OSI-930 and Crizotinib, the two TKIs can potentially target increased cell survival and metastasis of TNBC^58,59^. However, acquired drug resistance is frequently linked to activation of downstream PI3K/AKT signaling and remains a major obstacle limiting the clinical efficacy of TKIs^20,60^. Hence, combinatorial targeting of an aberrantly activated PI3K/AKT pathway in TNBC by a pBADSer99 inhibitor and TKIs could potentially abrogate the development of drug resistance and ameliorate the outcomes in TNBC. Herein, it was observed that the combination of NCK and OSI-930 or Crizotinib promoted apoptosis in a synergistic or additive manner through inhibition of BADSer99 phosphorylation. By inhibiting a core downstream effector of the PI3K/AKT and other pathways involved in acquired resistance, NCK synergizes with TKIs in reducing xenograft progression and TNBC lung metastasis in vivo. Additionally, both single agent treatments and combinatorial treatments extend the survival of the two TNBC PDX models examined herein. The effect of NCK and combinatorial treatments of NCK-OSI-930 or NCK-Crizotinib were observed to be more significant in the USTC-0 PDX which exhibited a higher pBADSer99/BAD ratio than USTC-1, and hence a BAD phosphorylation ratio may be a useful marker of potential drug efficacy. Given the robust heterogeneity of TNBC^61^, it is important to acknowledge that two PDX models might not fully reflect the complexity observed clinically. Whereas the findings herein provide valuable proof-of-concept, a greater number of PDX models, with detailed information of genetic and molecular characteristics, should be incorporated in future studies to observe potential benefit of the treatment regimens for TNBC patients with different molecular subtypes.

In summary, NCK was identified as a potent inhibitor of BADSer99 phosphorylation with high efficacy in reducing TNBC cell survival, xenograft growth and metastasis. Combination of NCK, with the dual VEGFR2 and c-Kit inhibitor, OSI-930, or dual c-Met and ALK inhibitor, Crizotinib, promoted apoptotic cell death by inhibiting BAD phosphorylation. Additionally, the combination of NCK with OSI-930 or Crizotinib demonstrated synergistic activity, yielding TNBC xenograft regression, abrogating lung metastasis and extending median survival in mice carrying TNBC PDXs. Hence, the present work identifies a highly synergistic and viable mechanistic based combination that targets BAD as a core effector of TNBC cell survival.

## Methods

### Cell culture and reagent

Human mammary carcinoma cell lines of the triple negative subtype MDA-MB-231, BT549, HCC1937 and Hs578T were purchased from the Procell Life Science Technology (Wuhan, China). MDA-MB-436, MDA-MB-468, HCC1937 and SUM159PT were purchased from BNBio Tech Co. Ltd (Beijing, China). SUM149PT and 4T1-luciferase cells were gifts from Tao Zhu’s laboratory (University of Science and Technology of China, China). All cell lines were maintained as per the manufacturer’s propagation instructions at 37 °C in a humidified incubator of 5% CO_2_. All in vitro cell based assays were performed in media containing a final concentration of 2% FBS. TNBC PDXs USTC-0 and USTC-1 were generated in the laboratory of Suling Liu (Fudan University, China) and have been previously described^40^. OSI-930 and Crizotinib were purchased from Selleckchem (Houston, TX, USA). Lipofectamine 3000 used for plasmid transfection was purchased from Thermo Fischer Scientific (Waltham, MA, USA). siRNA plasmid targeting BAD was purchased from GENEWIZ, Azenta Life Sciences (South Plainfield, NJ, USA) (Supplementary Fig. 20). Cas9-gRNA vector and pSpCas9(BB)-2A-Puro (PX459) were a gift from Feng Zhang (Addgene plasmid # 48139). For homology directed repair (HDR) assay (Supplementary Fig. 2), the hBADS99A sequence was designed and carried out following the protocol of the laboratory of Feng Zhang^62^. Antibodies used are listed in Supplementary Fig. 21A.

### Synthesis of piperazine based phenolic compounds

The synthesis of piperazine based phenolic compounds were performed as previously described by using Petasis borono-Mannich multicomponent reaction^13,63^. The desired phenolic compound product was obtained by separation using column chromatography. The structure of NCK was characterized by LCMS, ^1^H NMR, and ^13^C NMR spectroscopic techniques (Supplementary Fig. 4).

### In silico DFT calculations and bioinformatic analyses

The molecular structure of NCK was drawn using GaussView software^64^. The molecular geometry optimization of NCK was carried out by employing the density functional theory at B3LYP level and 6-31 G + (d,p) basis set by using Gaussian 09 software package^65^. The optimized structure of NCK has been used to calculate the molecular electrostatic potential (MEP), highest occupied molecular orbital (HOMO) and lowest unoccupied molecular orbital energy (LUMO) (Supplementary Fig. 5D, E). For bioinformatic analyses, the Scripps Research Institute’s AutoDock 4.2 Tools (v1.5.6) (ADT) was used to generate grid and docking parameter files. The reported crystal structure of 14-3-3 complexed BAD protein was retrieved from Protein Data Bank (PDB ID: 7Q16). The protein and ligand preparations were done by using BIOVIA discovery studio Visualizer. Visualization of docking analysis was examined by using BIOVIA Discovery Studio Visualizer (v21.1.0.202298), Pymol.

### Cancer compound library screening

The Cambridge Cancer Compound Library (L2300) was purchased from Selleckchem (Houston, TX, USA) and the detailed information of compounds are listed in Supplementary Fig. 11A. Cancer compound library screening was performed using IC_25_ values of anti-cancer compounds (predicted with Genomics of Drug Sensitivity in Cancer (https://www.cancerrxgene.org/) and NCK at 3-point dilutions (0.1, 1, 10 μM) in medium containing 2% FBS to identify potential synergistic combinations. In detail, MDA-MB-231 cells (5 ×10^3^) were seeded in 96-well plates in 80 μl of culture medium (2% FBS) and allowed to settle overnight. 24-hour post-seeding, 10 μl of compounds dissolved in culture medium were prepared and added to the plates to reach a final concentration of IC_25_ of the respective compound. 10 μl of NCK was also added into cell plates to yield a final concentration of 0.1, 1 or 10 μM. After 72-hours of treatment, cell viability was determined with AlamarBlue reagent and fluorescence was measured using a Tecan microplate reader, as previously described^29^. A schematic of the high-throughput screening assay is included in Supplementary Fig. 11B.

### TNBC tissue microarrays and immunohistochemistry (IHC)

The TNBC tissue microarray (ZL-Brc3N961) was obtained from Zhuoli Biotech Co., Ltd. (Shanghai, China). Consent for the use of the tissue samples and clinical data were obtained by Zhuoli Biotech Co., Ltd. (Shanghai, China). IHC staining and scoring were performed as previously described^66^. The corresponding antibodies used here are listed in Supplementary Fig. 21A. The staining results were assessed and confirmed by two independent researchers blinded to the clinical data.

### RNA sequencing

RNA was isolated from MDA-MB-231 cells following treatment of 0 and 5 μM of NCK or NPB using TRIzol reagent (Sigma-Aldrich, MO, USA), as previously described^67^. RNA-seq was subsequently performed by BGI (Shenzhen, China). The cDNA library was prepared with the commercial Illumina library preparation kits (TruSeq Stranded RNA LT Ribo-Zero H/M/R Kit) according to the manufacturer’s protocols. Quality control was performed on the raw dNA-Seq reads (FastQC), and the adapters were cut (Trim Galore) and mapped with hg38 genome (HISAT2). Differentially expressed transcripts were defined with Cuffdiff tools and visualized using R software.

### Oncogenic and SPR analyses

AlamarBlue® viability, total cell number, foci formation, growth in 3D Matrigel culture, apoptosis and cell cycle flow cytometry assays were performed as previously described^68^. All in vitro based assays were performed in medium containing 2% FBS. Transwell migration assay was performed as previously described^69^. Briefly, 1 × 10^5^ cells suspended in serum-free medium containing 0.2% BSA were plated in the top chamber of 8 μm pore size (Corning, MA, USA). Medium with 10% serum was added to the lower chamber and cells were allowed to migrate for 24-48 hours (depending on cell line) before fixing with formalin, permeabilizing with methanol and staining with crystal violet. Real time migration assay was performed as previously reported by using xCELLigence system^70,71^. Live/Dead cells staining was performed following manufacturer’s instruction using LIVE/DEAD™ Cell Imaging Kit (Thermo Fisher Scientific, MA, USA). CASPASE 3/7 assay (Biovision, CA, USA) was performed following the manufacturer’s protocol. Western blot analyses were performed as previously described^72^. All blots were derived from the same experiment and were processed in parallel. The corresponding antibodies used are listed in Supplementary Fig. 21A and original blots are provided in Supplementary Fig. 22. Molecular interactions were analyzed by SPR using a BIAcore-2000 system (BIAcore AB, Uppsala, Sweden). Recombinant human BAD (Novoprotein, Suzhou, China) was immobilized on a sensor chip and analyzed as per the manufacturer’s protocol and as previously described^73^. Combination index (CI) analysis was performed using the Chou-Talalay^74^ and SynergyFinder^75^ CI analysis method.

### In vivo studies

All animal experiments were approved by the Institutional Animal Care and Use Committee of the Laboratory Animal Centre of Peking University Shenzhen Graduate School (permit YW; the permit from Tsinghua Shenzhen International Graduate School is “Ethical Development no. 37 (Year 2019)”. The schematic representation of in vivo studies is shown in Supplementary Fig. 23. Mice were housed in a controlled atmosphere (25 ± 1 °C at 50% relative humidity) under a 12-h light/12-h dark cycle. Animals had free access to food and water at all times. To establish the orthotopic xenograft model for single drug and combination studies, MDA-MB-231 (1 × 10^7^ cells in 100 μL PBS containing growth factor reduced 25% Matrigel) or 4T1-luciferase cells (7 × 10^4^ cells in 100 μL PBS containing growth factor reduced 25% Matrigel) were injected orthotopically into the right fourth mammary fat pad of 8-week-old female BALB/c-nude or BALB/c mice (Experimental Animal Center, Guangzhou, China). For the intravenous metastasis model, MDA-MB-231 (1 × 10^6^ cells in 100 μL PBS) or 4T1-luciferase cells (1 × 10^5^ cells in 100 μL PBS) were injected intravenously into the tail vein of 8-week-old female BALB/c-nude (MDA-MB-231) or BALB/c (4T1-luciferase) mice (Experimental Animal Center, Guangzhou, China). In vivo bioluminescent imaging was performed to determine the incidence and metastatic burden of luciferase-labeled 4T1 cells. Mice were injected i.p. with 10 mg/ml of D-luciferin (Gold Biotechnology, MO, USA) and imaged using a PerkinElmer IVIS Spectrum system. PDX were generated as previously described by transplanting fresh fragments orthotopically into 8-week-old female nonobese diabetic (NOD)/severe combined immunodeficient (SCID) mice (Beijing Vital River Laboratory Animal Technology Co., Beijing, China) (P1, passage 1)^40^. The P1 xenografts were then fragmented, digested into single-cell suspension and implanted into mammary fat pad (100 μL PBS containing growth factor reduced 50% Matrigel) of NOD/SCID mice. Once orthotopic xenografts reached an approximate size of 100 mm^3^, the mice were randomly divided into three groups (*n* = 8) for single drug studies and six groups (*n* = 6) for combination studies and Kaplan-Meier (KM) analyses. For the single drug study, vehicle (4.6% DMSO, 14.3% PEG400, 9.7% water pH 5.0 and 71.4% N-saline) and NCK at 5 and 20 mg/kg were intraperitoneally injected daily for 3 weeks. In the combination and KM studies, vehicle, NCK (20 mg/kg) or OSI-930 (50 mg/kg) were intraperitoneally injected daily (*q.d.*) for 3 weeks, whereas Crizotinib (50 mg/kg) was injected intraperitoneally biweekly (*b.i.w.*) for 3 weeks. Animal body weight and xenograft size were measured daily using an electronic balance and a digital caliper, respectively. Xenograft volume (mm^3^) was calculated by using the formula: 0.52 × length × [width]^2^. Complete response (CR) was achieved if the average xenograft volume for the treatment group was unable to be determined for two or more consecutive measurements. Partial response (PR) was achieved if the average xenograft volume reduced to <50 mm^3^ for two or more consecutive measurements. After 3-week drug treatment, all mice were sacrificed by CO_2_ inhalation. Tumor and vital organs were weighed and isolated for further analysis. IHC analysis of xenograft histology sections and quantitative polymerase chain reaction (qPCR) were performed as previously described^76^. The corresponding antibodies and the sequences of the oligonucleotide primers used are listed in Supplementary Fig. 21A and Supplementary Fig. 21B respectively. For PDX, xenograft growth was monitored daily by measurement of the xenograft volume until mice humane endpoint ( ~ 1000 mm^3^).

### Toxicity

The toxicity study design was evaluated according to the previously published procedure^77^ with slight modification. In this model, ICR mice (Experimental Animal Center, Guangzhou, China) were injected i.p. with vehicle or NCK of 20 and 50 mg/kg continuously for 14 days (*n* = 8). Animals were given ad libitum access to food and water. The changes in body weight, food and water consumptions were recorded every day. Signs of toxicity, mortality and behavioral patterns were monitored daily after administration. At the end of the study, all mice were sacrificed by CO_2_ inhalation, and the blood samples were collected to evaluate the hematological and biochemical parameters (Servicebio, Wuhan, China). All vital organs (heart, liver, spleen, lung, kidney, stomach, small intestinal and colon) were weighed and isolated at necropsy and histopathological examination performed.

### Statistical Analysis

Two-tailed unpaired Student’s t test and one-way ANOVA analysis followed by Bonferroni’s posttest correction were used to calculate the statistical significance of two or multiple treatment groups, respectively. The level of significance was set as *P < 0.05, **P < 0.01, and ***P < 0.001. All group data were presented as the means ± standard deviation (SD). All analyses were done using GraphPad Prism software (version 5.0).

### Reporting summary

Further information on research design is available in the Nature Research Reporting Summary linked to this article.

### Supplementary information

Supplementary Information

Reporting Summary

## Data Availability

The data generated and analyzed during this study are available from 10.5281/zenodo.10129841. All other data supporting the findings of this study are available within the paper and its Supplementary Figure or from the corresponding author upon reasonable request.
