# Supplementary material for: Vertical pathway inhibition of receptor tyrosine kinases and BAD with synergistic efficacy in triple negative breast cancer
Source: NPJ Precis Oncol. 2024 Jan 10;8:8. doi: 10.1038/s41698-023-00489-3 (PMC10781691; doi:10.1038/s41698-023-00489-3)
Supplement: Supplementary file 1 — Supplementary Information [file 41698_2023_489_MOESM1_ESM.pdf]

Vertical pathway inhibition of receptor tyrosine kinases and BAD with synergistic efficacy in triple negative breast cancer

Yan Qin Tan, Yi-Shiou Chiou, Hui Guo, Shuwei Zhang, Xiaoming Huang, Dukanya Dukanya, Arun M. Kumar, Shreeja Basappa, Suling Liu, Tao Zhu, Basappa Basappa, Vijay Pandey, and Peter E. Lobie

Supplementary Figures

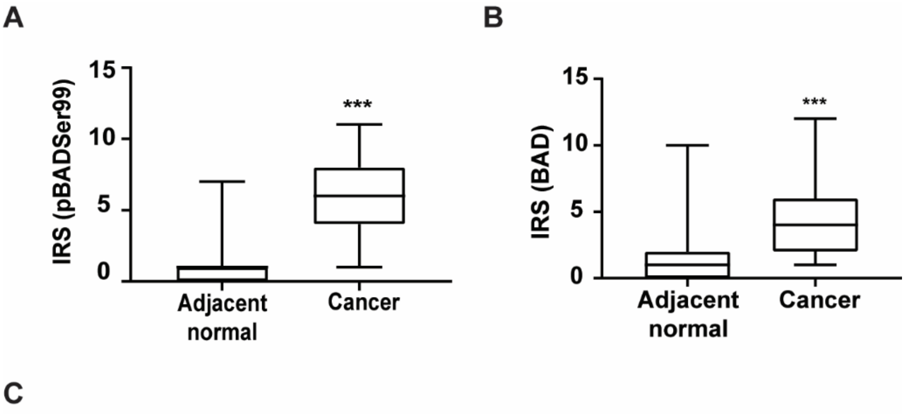

C

Cohort	Total (N)	Positive (%)	Negative (%)	*P*-value
Age				0.880
<=55	18	67	33	
>55	28	68	32	
Grade				<0.0001***
I	18	78	22	
II	25	64	36	
III	3	33	67	
Lymph Node Metastasis				0.552
0	28	68	32	
1	6	67	33	
2	4	75	25	
3	3	67	33	
MKI67				<0.0001***
Low	10	50	50	
Moderate	20	80	20	
Strong	16	63	38	

*$P < 0.05$, **$P < 0.01$, and ***$P < 0.001$.

Supplementary Figure 1: (A) pBADSer99 was detected using immunohistochemistry (IHC) in adjacent normal (AD) and TNBC tissue specimens. Analysis of pBADSer99 levels and BAD expression in AD and TNBC tissue specimens by immunoreactive score (IRS) was performed. Data represent means ± SD. *$P < 0.05$, **$P < 0.01$, and ***$P < 0.001$. (B) BAD expression

was detected using immunohistochemistry (IHC) in adjacent normal (AD) and TNBC tissue specimens. Analysis of pBADSer99 levels and BAD expression in AD and TNBC tissue specimens by immunoreactive score (IRS) was performed. Data represent means ± SD. $*P < 0.05$, $**P < 0.01$, and $***P < 0.001$. (C) Correlation analysis between pBADSer99 level and clinicopathological features of TNBC.

hBAD sequence:

ATGTTCCAGATCCCAGAGTTTGAGCCGAGTGAGCAGGAAGACTCCAGCTCTGCA
GAGAGGGGCCTGGGCCCCAGCCCCGCAGGGGACGGGCCCTCAGGCTCCGGCAAG
CATCATCGCCAGGCCCCAGGCCTCCTGTGGGACGCCAGTCACCAGCAGGAGCAG
CCAACCAGCAGCAGCCATCATGGAGGCGCTGGGGCTGTGGAGATCCGGAGTCGC
CACAGCTCCTACCCCGCGGGGACGGAGGACGACGAAGGGATGGGGGAGGAGCC
CAGCCCCTTTCGGGGCCGCTCGCGCTCGGCGCCCCCCAACCTCTGGGCAGCACAG
CGCTATGGCCGCGAGCTCCGGAGGATGAGTGACGAGTTTGTGGACTCCTTTAAG
AAGGGACTTCCTCGCCCGAAGAGCGCGGGCACAGCAACGCAGATGCGGCAAAG
CTCCAGCTGGACGCGAGTCTTCCAGTCCTGGTGGGATCGGAACTTGGGCAGGGG
AAGCTCCGCCCCCTCCCAGTGA

hBAD S99A (TCG>GCG):

ATGTTCCAGATCCCAGAGTTTGAGCCGAGTGAGCAGGAAGACTCCAGCTCTGCA
GAGAGGGGCCTGGGCCCCAGCCCCGCAGGGGACGGGCCCTCAGGCTCCGGCAAG
CATCATCGCCAGGCCCCAGGCCTCCTGTGGGACGCCAGTCACCAGCAGGAGCAG
CCAACCAGCAGCAGCCATCATGGAGGCGCTGGGGCTGTGGAGATCCGGAGTCGC
CACAGCTCCTACCCCGCGGGGACGGAGGACGACGAAGGGATGGGGGAGGAGCC
CAGC**G**C**G**TTTCGGGGCCGCTCGCGC**G**CGGCGCCCCCCAACCTCTGGGCAGCACA
GCGCTATGGCCGCGAGCTCCGGAGGATGAGTGACGAGTTTGTGGACTCCTTTAA
GAAGGGACTTCCTCGCCCGAAGAGCGCGGGCACAGCAACGCAGATGCGGCAAA
GCTCCAGCTGGACGCGAGTCTTCCAGTCCTGGTGGGATCGGAACTTGGGCAGGG
GAAGCTCCGCCCCCTCCCAGTGA

Supplementary Figure 2: The CDSs of hBAD and hBAD-S99A

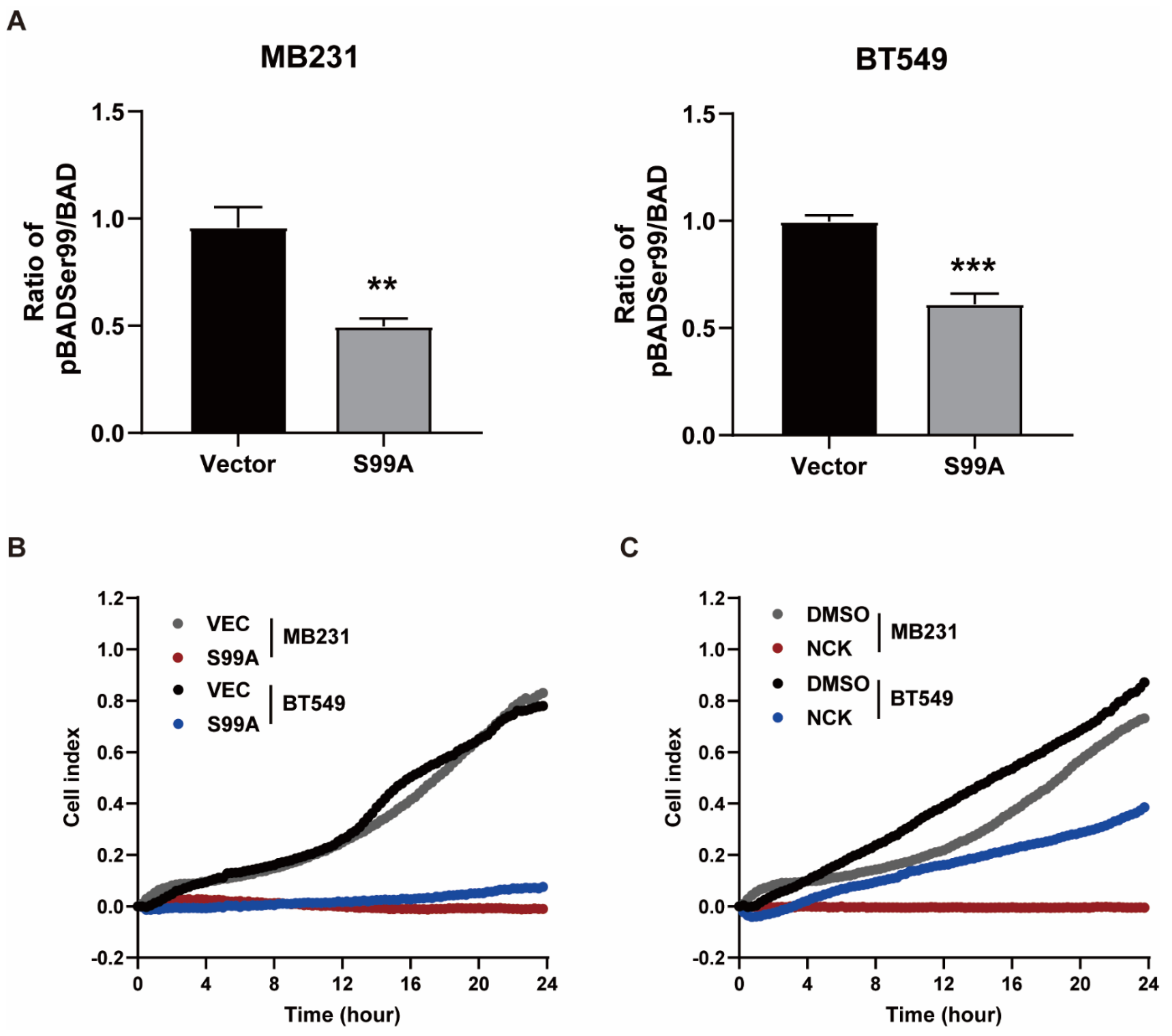

Supplementary Figure 3: (A) Densitometric analysis of western blots in Figure 1B-C. MDA-MB-231 and BT549 cells were transfected with pBADS99A knock in plasmid or vector control (n=3). Densitometric analysis of protein blots was determined using ImageJ software (https://imagej.nih.gov/ij/). **P* < 0.05, ***P* < 0.01, and ****P* < 0.001. (B-C) Dynamic real time monitoring of TNBC cell migration after (B) transfection with hBADS99A or (C) treatment with 5 μM NCK using the xCELLigence system. 40,000 cells were seeded into each well 6-hour post-transfection. Cell migration kinetics were recorded by Roche xCELLigence Real-Time Cell Analyzer (RTCA) DP instrument for 24 hours.

A

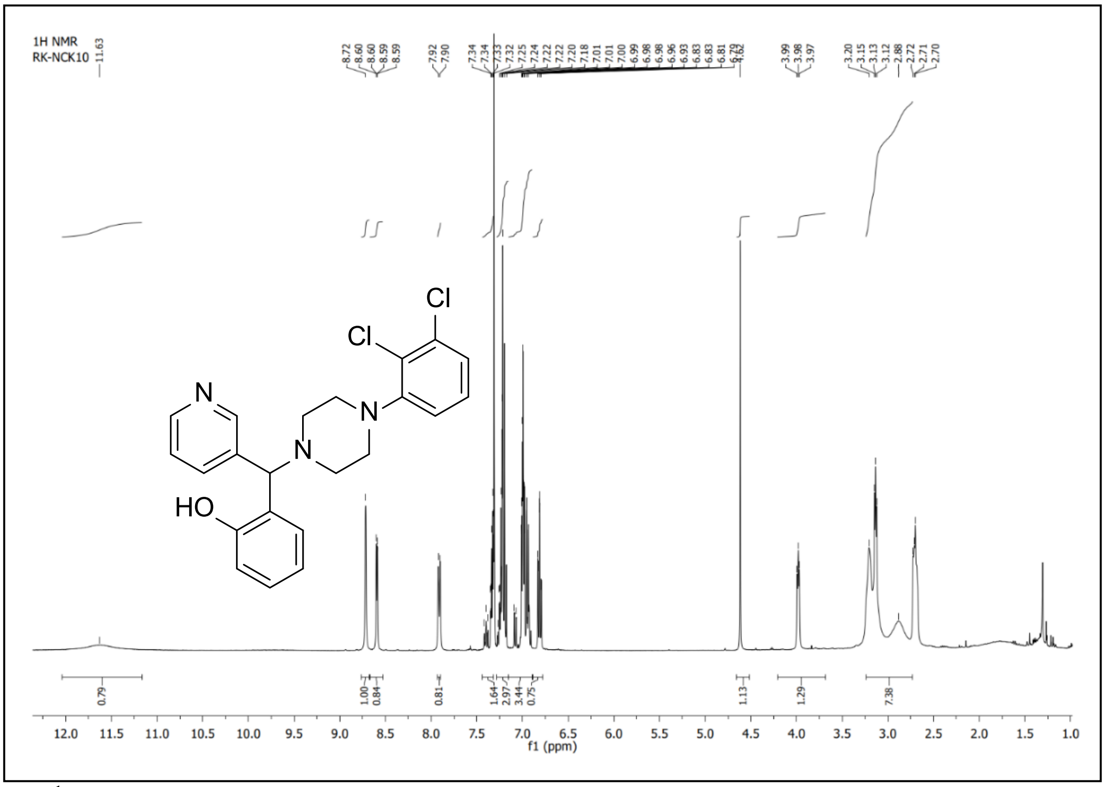

^{1}H NMR spectrum of 2-((4-(2,3-dichlorophenyl)piperazin-1-yl)(pyridin-3-yl)methyl) phenol

B

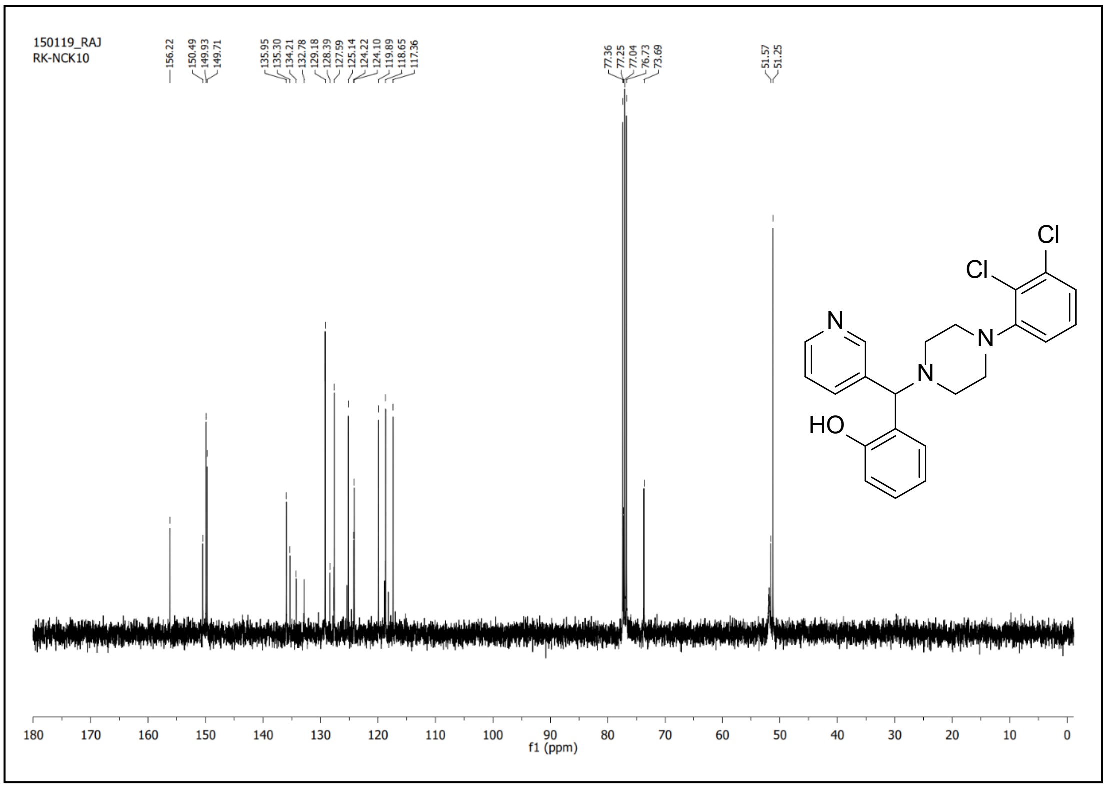

^{13}C NMR spectrum of 2-((4-(2,3-dichlorophenyl)piperazin-1-yl)(pyridin-3-yl)methyl) phenol

C

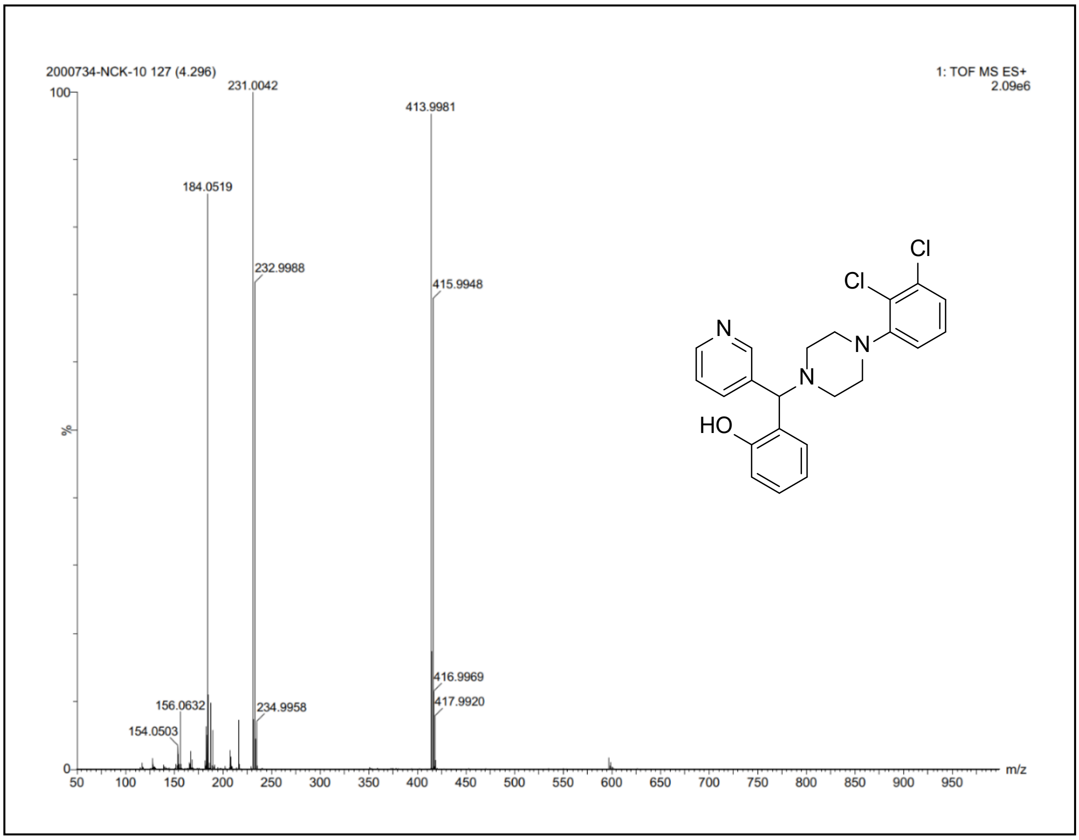

Mass spectrum of 2-((4-(2,3-dichlorophenyl)piperazin-1-yl)(pyridin-3-yl)methyl) phenol

D

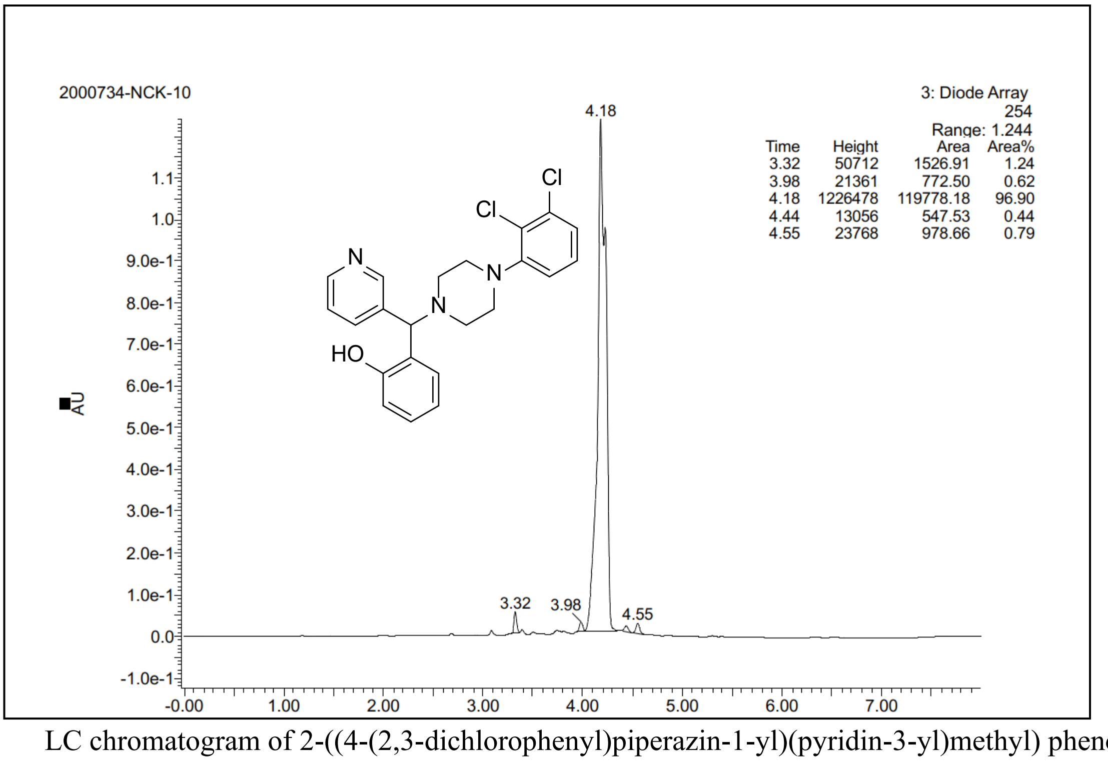

LC chromatogram of 2-((4-(2,3-dichlorophenyl)piperazin-1-yl)(pyridin-3-yl)methyl) phenol

E

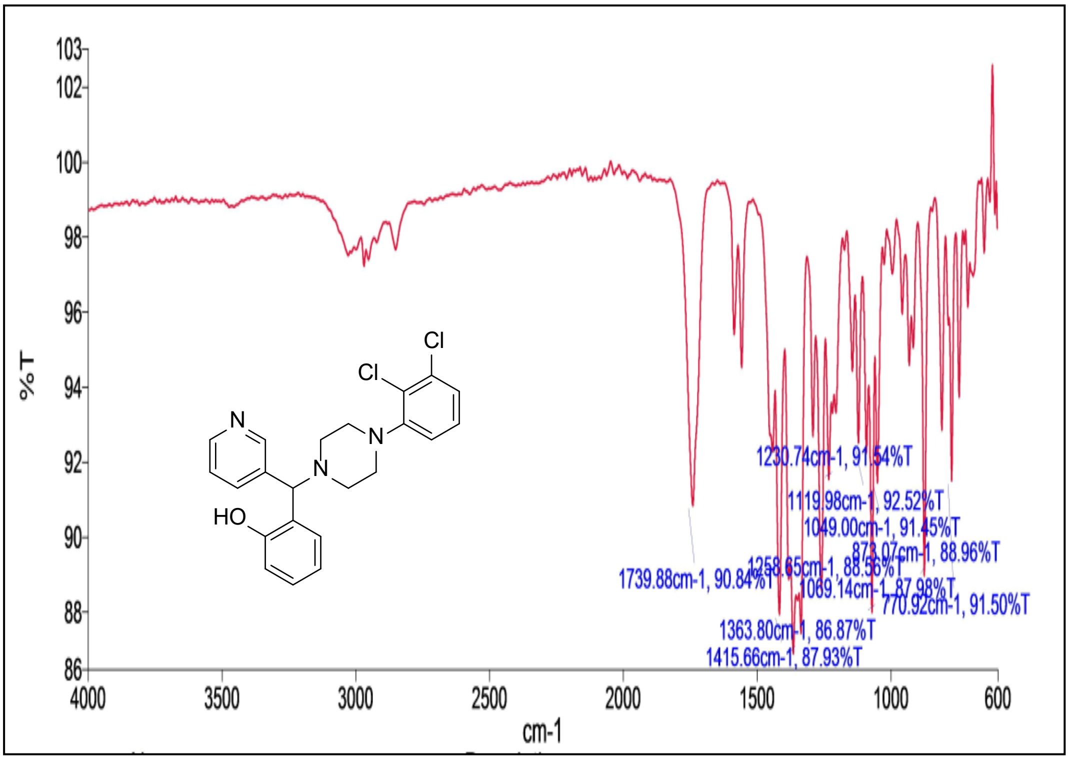

IR spectrum of 2-((4-(2,3-dichlorophenyl)piperazin-1-yl)(pyridin-3-yl)methyl) phenol

Supplementary Figure 4: (A) ^{1}H NMR spectrum of NCK. (B) ^{13}C NMR spectrum of NCK. (C) Mass spectrum of NCK. (D) LC chromatogram of NCK. (E) IR spectrum of NCK.

A

	IC_{50} ± SD, µM
**MDA-MB-231**	4.94±2.80
**BT549**	12.13±1.36

B

C

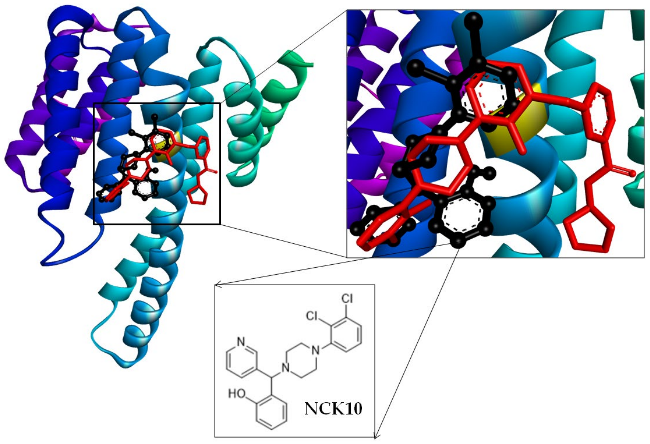

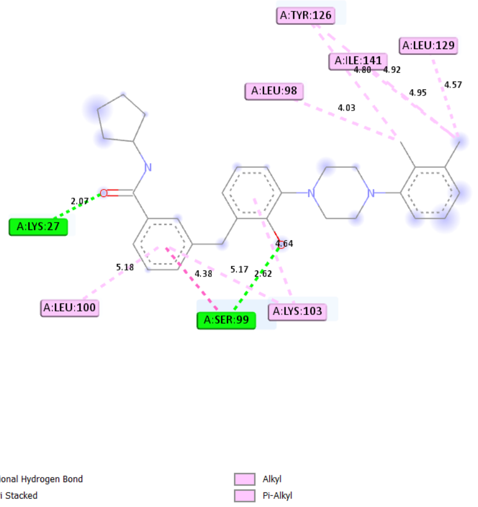

D

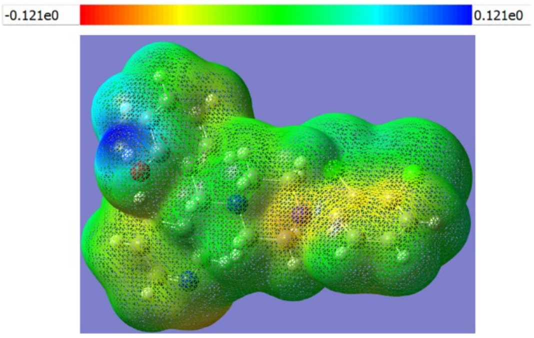

E

Orbital energy	DFT/B3LYP
E_{HOMO}	-7.21 eV
E_{LUMO}	-4.79 eV
$\Delta E_{LUMO-HOMO}$	2.42 eV
**Ionization potential (*I*)**	7.21 eV
**Electron affinity (A)**	4.79 eV
**Hardness (η)**	1.21 eV
**Softness (S)**	0.41 eV^{-1}
**Chemical potential (µ)**	6.00 eV
**Electronegativity (χ)**	6.00 eV
**Electrophilicity (ψ)**	14.86eV

Supplementary Figure 5: (A) IC_{50} of NCK in MDA-MB-231 and BT549 cells measured by using AlamarBlue assay. Data represent means ± SD. (B) Cartoon representation of the compounds NPB (red) and NCK (black) (depicted in ball & stick model) docked to the BAD protein, occupying the same groove with the BAD Serine 99 residue (yellow) nearby. (C) 2D structure representation of compound NPB interacting with BAD protein residues. (D) Cartoon representation of docked compound and molecular electrostatic potential surfaces of NCK. The color code of the compound lies in the range of -0.121e0 to +0.121e0. Red and blue color in the MEP structure point to more electron rich and electron poor regions respectively. (E) The

calculated FMOs and Global chemical reactivity descriptors. Computed E_{HOMO}, E_{LUMO}, frontier molecular orbital's (FMO) energy gap ($\Delta E_{LUMO-HOMO}$), ionization potential (I), electron affinity (A), chemical hardness (η), chemical softness (S), Chemical potential (μ), electronegativity (χ) and electrophilicity index (ψ) in eV of NCK.

A

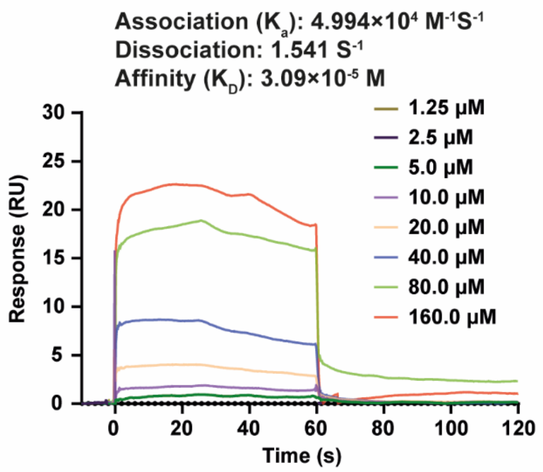
Association (Ka): 4.994×10^4 M^-1S^-1
Dissociation: 1.541 S^-1
Affinity (KD): 3.09×10^-5 M
1.25 μM
2.5 μM
5.0 μM
10.0 μM
20.0 μM
40.0 μM
80.0 μM
160.0 μM
Response (RU)
Time (s)

B

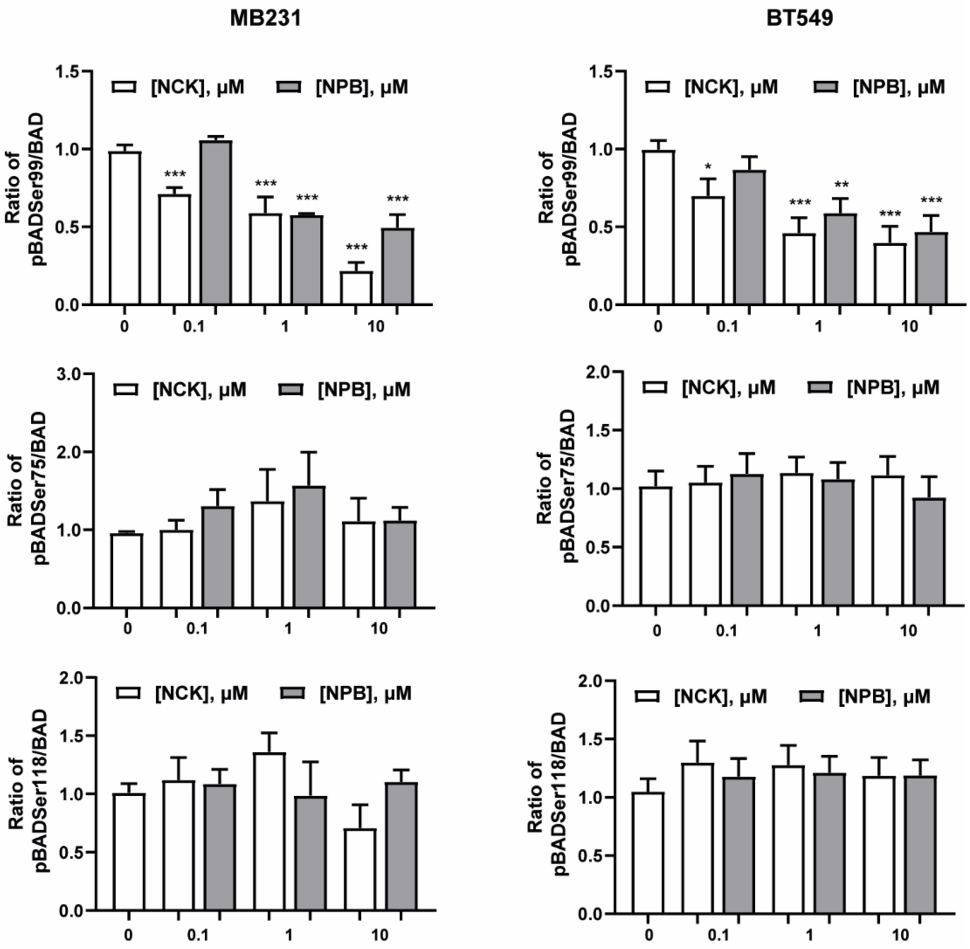
MB231
BT549
[NCK], μM
[NPB], μM
Ratio of pBADSer99/BAD
Ratio of pBADSer75/BAD
Ratio of pBADSer118/BAD
0
0.1
1
10

C

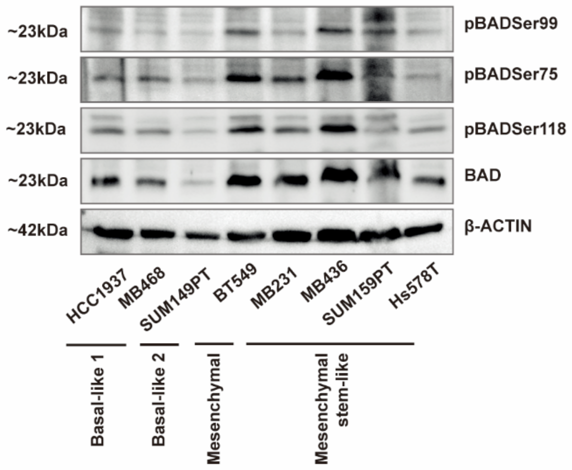

D

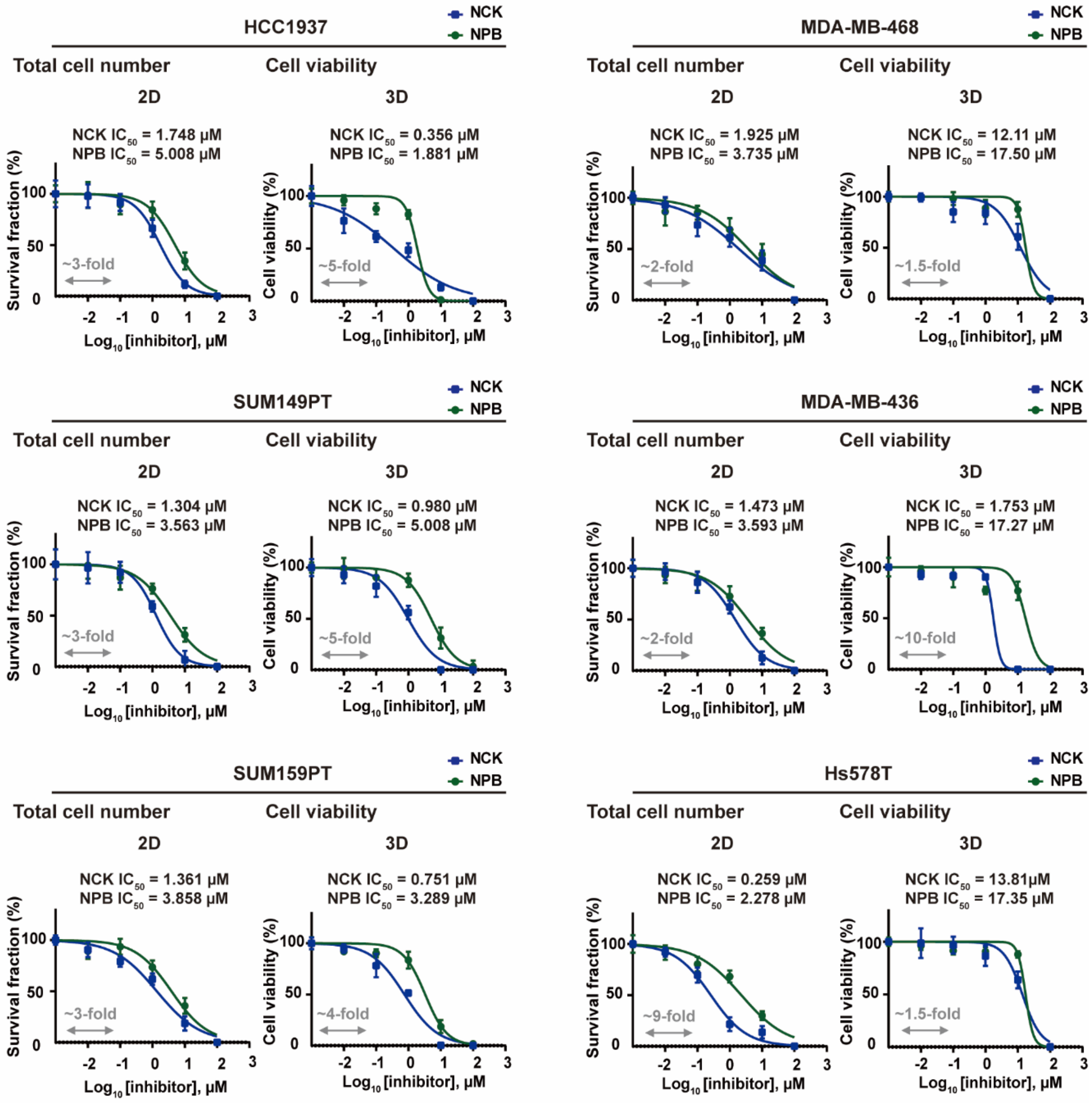

Supplementary Figure 6: (A) Sensorgrams obtained by SPR analysis of NPB with the BAD protein. The BAD protein was immobilized on the surface of a CM5 sensor chip. A solution of

NPB at variable concentrations (1.25–160 μM) was injected to generate the binding responses (RU) recorded as a function of time (s). The results were analyzed using BIA evaluation 4.1. (B) Densitometric analysis of western blots in Figure 2E. MDA-MB-231 and BT549 cells were treated with 0-10 μM NCK or NPB (n=3). Densitometric analysis of protein blots was determined using ImageJ software (https://imagej.nih.gov/ij/). Statistical changes were assessed by using ANOVA. *$P < 0.05$, **$P < 0.01$, and ***$P < 0.001$. (C) Western blot analysis was used to assess the levels of pBADSer99, pBADSer75, pBADSer118 and BAD expression in TNBC cell lines. β-ACTIN was used as input control for cell lysate. The sizes of detected protein bands in kDa are shown on the left. (D) Dose-dependent effect of NPB and NCK in 2D and 3D culture on TNBC cell viability (in HCC1937, MDA-MB-468, SUM149PT, MDA-MB-436, SUM159PT and Hs578T cell lines) measured by using total cell number assay and AlamarBlue assay respectively (n=3).

A

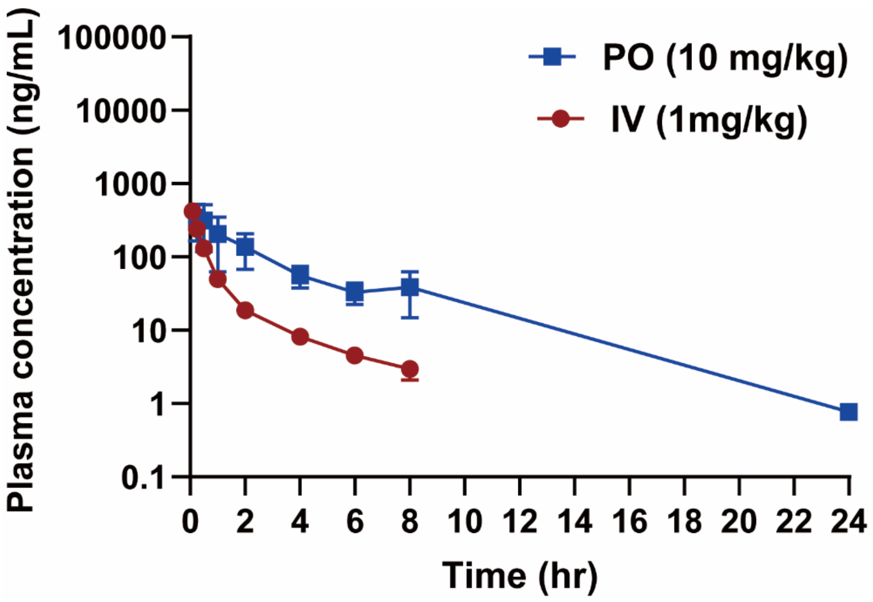

B

PK parameters	Unit	IV	PO
Cl_obs	mL/min/kg	58.3	-
$T_{1/2}$	h	3.35	2.68
T_{max}	h	-	0.33
C_0	ng/mL	554	-
C_{max}	ng/mL	-	346
AUC_{last}	h*ng/mL	280	1054
AUC_{Inf}	h*ng/mL	288	1072
AUC_%Extrap_obs	%	2.51	2.14
MRT_{Inf}_obs	h	1.72	4.00
AUC_{last}/D	h*mg/mL	280	105
V_{ss}_obs	L/kg	5.93	-
F	%	-	37.2

Supplementary Figure 7: (A) Plasma concentration of NCK after intravenous (IV, 1 mg/kg) (red) or oral (PO, 10 mg/kg) (blue) administration in SD rats. Results represent the mean ± SD of three animals. (B) Pharmacokinetic parameters of NCK in plasma after IV injection (1 mg/kg) or oral administration (10 mg/kg). AUC_{inf}: Area Under the Plasma Concentration-Time Curve from t=0 to infinity; AUC_{last}: Area Under the Plasma Concentration-Time Curve from t=0 to last measurable positive concentration; AUC_%Extrap_obs: Percent Extrapolated AUC from t_{last} to infinity; C_0: Initial concentration in plasma; C_{max}: Peak concentration in plasma; Cl_obs: Plasma systemic clearance; F: Absolute oral bioavailability; MRT_{Inf}_obs: Mean residence time from t=0 to infinity ;$T_{1/2}$: Elimination half-life; T_{max}: Time of peak concentration in plasma; V_{ss}_obs: Volume of Distribution at Steady-state

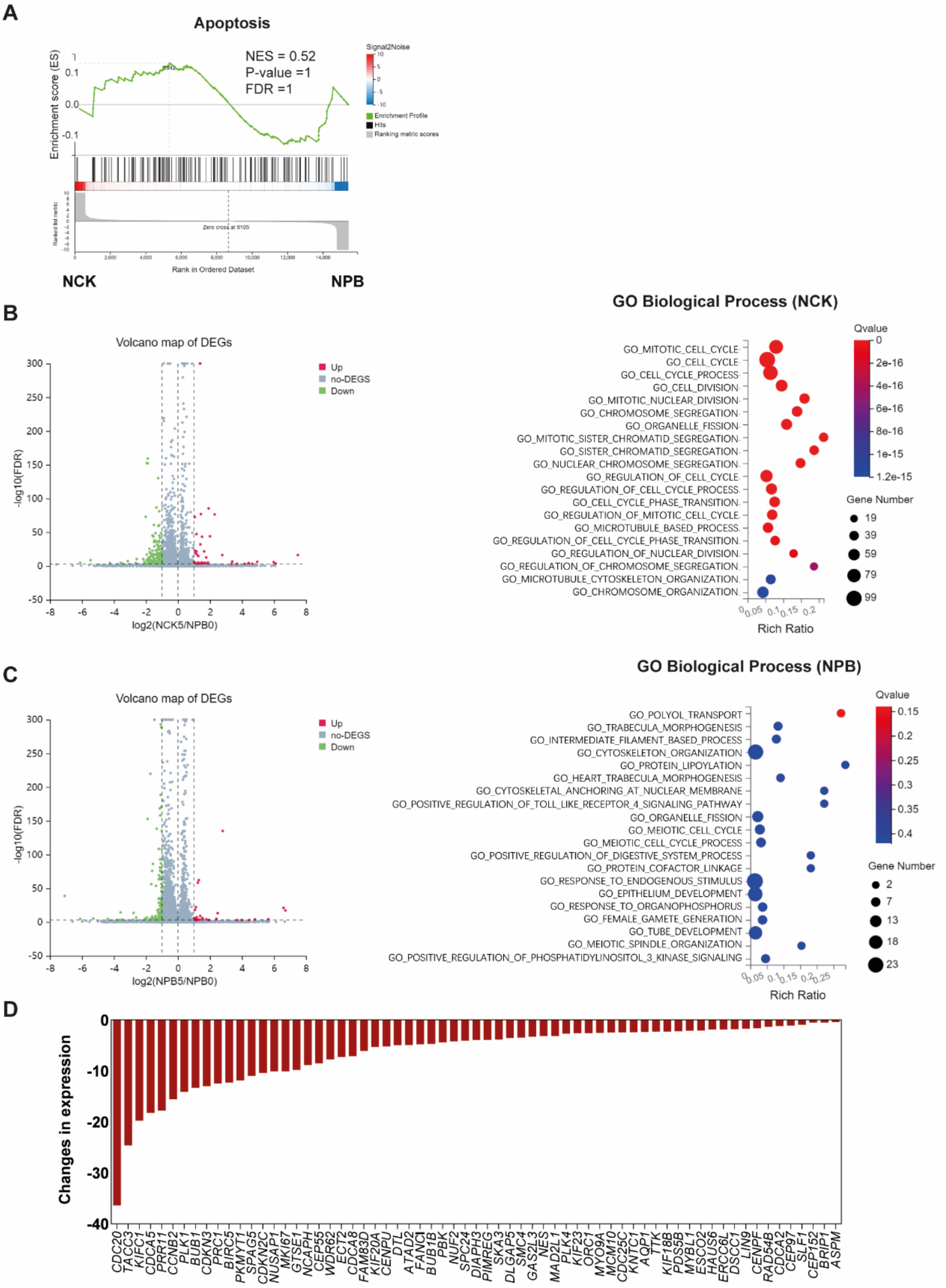

Supplementary Figure 8: (A) GSEA analyses of gene sets for apoptosis. NES, normalized enrichment score. FDR, false discovery rate. (B) GO biological process analysis of the DEGs after 5 μM NCK treatment. Volcano map of differential expressed genes (DEGs) after NCK treatment. Advanced bubble chart shows enrichment of DEGs in GO biological processes after treatment with 5 μM NCK. (C) GO biological process analysis of the DEGs after 5 μM NPB

treatment. Volcano map of differential expressed genes (DEGs) after NPB treatment. Advanced bubble chart shows enrichment of DEGs in GO biological processes after treatment with 5 μM NPB. (D) Bar charts depicting cell cycle related DEGs upregulated or downregulated by 5 μM NCK treatment.

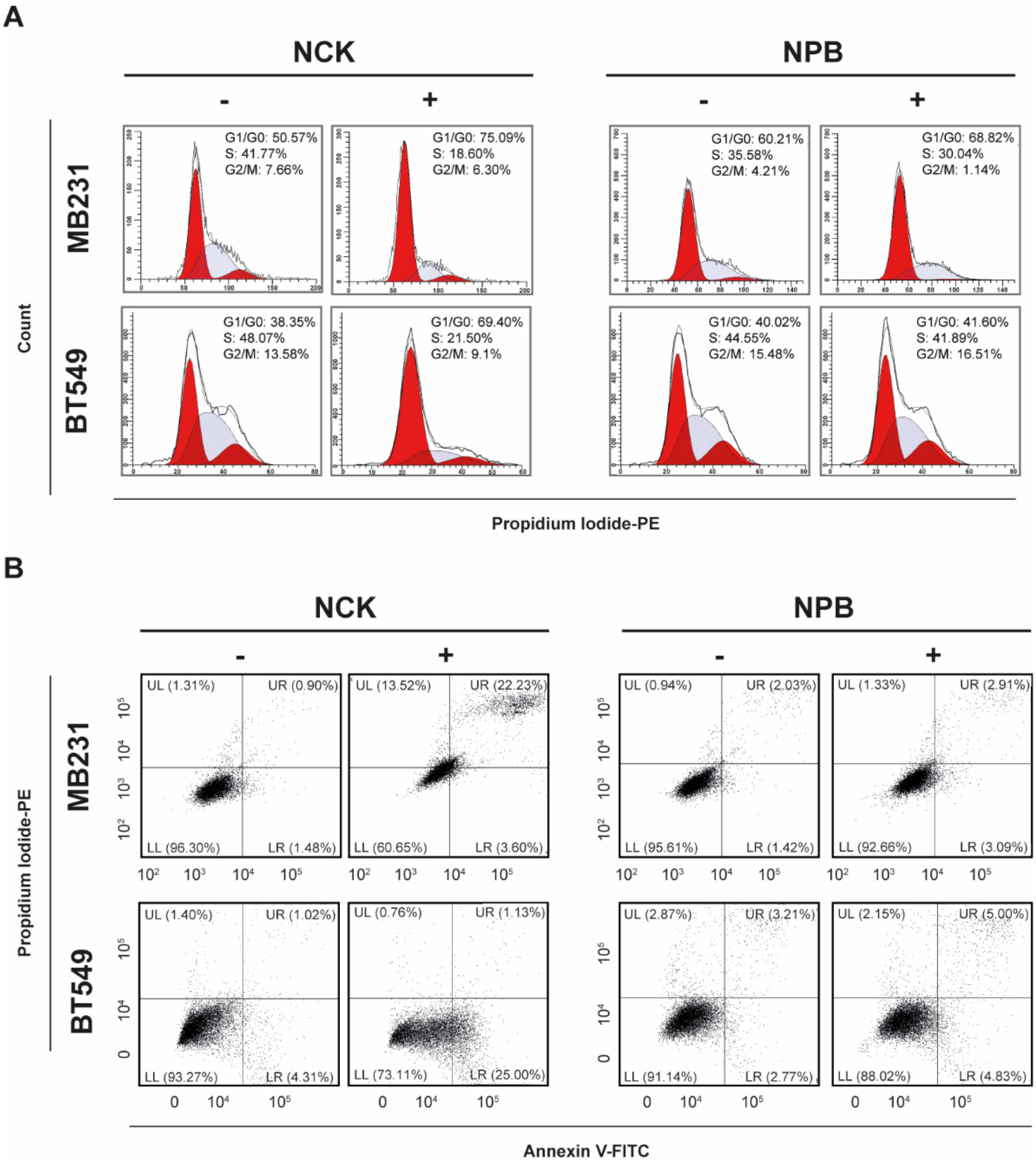

Supplementary Figure 9: (A) Representative flow cytometry plots using PI staining for DNA of MDA-MB-231 cells and BT549 cells measured after treatment with 5 µM NCK or NPB using flow cytometry analysis at 72 hours as described in materials and methods. (B) Representative flow cytometry plots of Annexin-V and propidium iodide (PI) stained apoptotic cell death of MDA-MB-231 cells and BT549 cells measured after treatment with 5 µM NCK or NPB using flow cytometry analysis. Annexin V-FITC staining is indicated on the x axis, and PI staining is indicated on the y axis. The lower left quadrants represent live cells, the lower right quadrants represent early apoptotic cells, the upper left quadrants represent necrotic cells, and the upper right quadrants display late apoptotic cells. Acquisition of Annexin V and PI data are presented as a percentage (%) in each quadrant. Early apoptotic cells are referred to as Annexin-V positive and late apoptotic cells are referred to as Annexin-V and PI double positive.

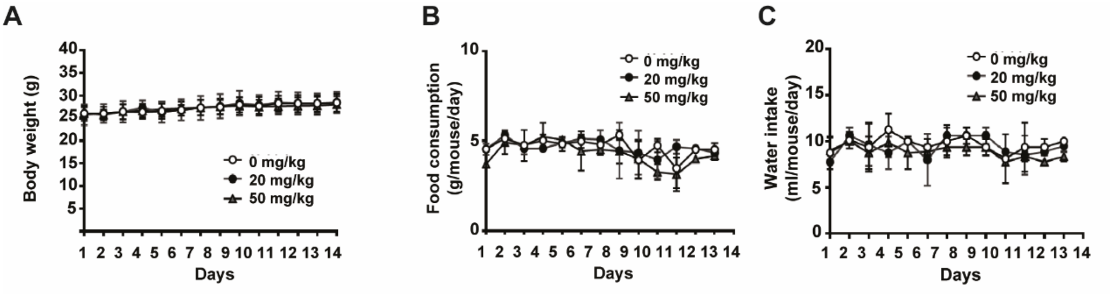

D

%	0 mg/kg	20 mg/kg	50 mg/kg	*P*-value
**Relative Lung Weight**	0.71±0.08	0.72±0.05	0.72±0.02	0.855
**Relative Liver Weight**	5.06±0.61	4.75±0.35	4.74±0.50	0.353
**Relative Spleen Weight**	0.40±0.05	0.37±0.04	0.42±0.05	0.148
**Relative Heart Weight**	0.49±0.05	0.47±0.04	0.47±0.05	0.462
**Relative Kidney Weight**	1.35±0.18	1.29±0.14	1.32±0.14	0.686
**Colon Length (cm)**	13.50±0.38	13.94±0.73	14.00±0.71	0.242

Data represent means ± SD. $*P < 0.05$, $**P < 0.01$, and $***P < 0.001$.

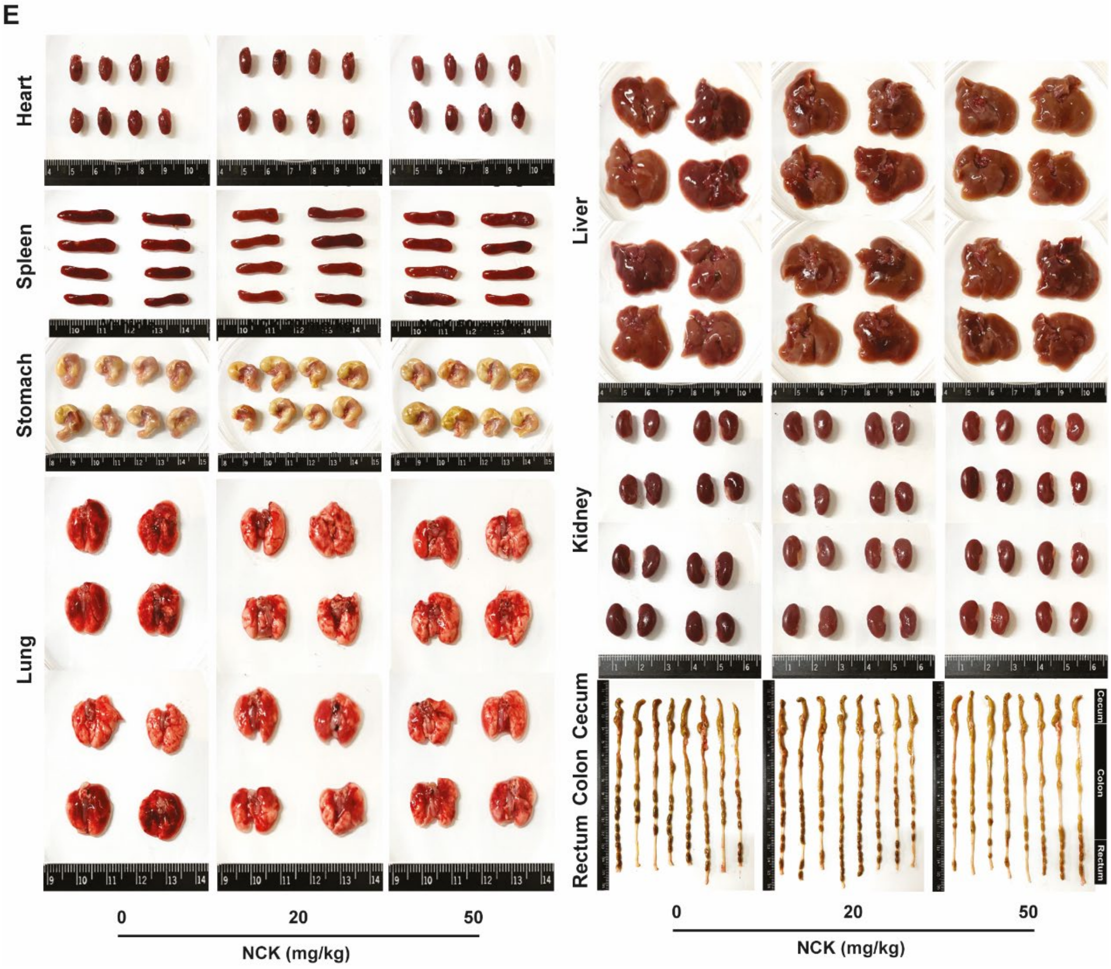

F

	0 mg/kg	20 mg/kg	50 mg/kg	*P*-value
**Hematology**				
**WBC (10^3/μL)**	4.32±1.79	3.35±1.86	2.40±0.72	0.135
**RBC (10^6/μL)**	9.88±2.14	9.72±1.24	9.69±0.65	0.963
**RDW (%)**	12.18±0.81	12.34±1.27	12.49±0.80	0.819
**HGB (g/L)**	167.88±40.67	164.00±12.98	159.50±13.16	0.811
**HCT (%)**	51.69±12.17	49.85±3.41	49.48±4.51	0.831
**PLT (10^3/μL)**	964.38±488.74	1166.50±514.61	1213.38±672.84	0.650
**PDW (%)**	17.13±0.39	17.10±0.78	16.63±0.32	0.137
**MCV (fL)**	52.14±1.85	51.36±2.39	51.16±1.93	0.616
**MCH (pg)**	16.83±0.74	16.83±0.45	16.39±0.45	0.226
**MCHC (g/L)**	323.75±6.96	325.25±4.43	322.25±7.92	0.667
**MPV (fL)**	6.90±0.63	6.83±0.68	6.59±0.42	0.548
**MON (%)**	4.60±2.44	4.86±3.14	6.91±2.64	0.209
**GRAN (%)**	26.21±10.57	26.48±17.89	34.96±6.23	0.303
**LYM (%)**	69.19±11.75	69.35±20.95	56.46±8.82	0.160
**Serum Biochemistry**				
**ALT/GOT (U/L)**	91.56±62.62	96.93±35.10	88.62±25.98	0.947
**AST/GPT (U/L)**	402.45±188.38	484.05±222.88	424.22±193.03	0.773
**CK (U/L)**	11423.01±10618.40	9143.94±6642.05	9769.45±8059.82	0.894
**CREA (μmol/L)**	24.33±6.74	24.70±11.51	23.00±7.39	0.940
**GLU (mg/dL)**	87.54±6.80	84.78±6.54	85.92±4.90	0.741
**GSP (mmol/L)**	5.28±1.02	5.51±0.56	5.37±1.04	0.911
**LDH (U/L)**	66.77±18.25	67.78±28.62	67.63±24.38	0.997
**UREA (mmol/L)**	14.49±4.53	18.80±5.09	16.98±3.42	0.265

Data represent means ± SD. $*P < 0.05$, $**P < 0.01$, and $***P < 0.001$.

Supplementary Figure 10: (A) Individual daily changes in body weight of female ICR mice administrated with NCK during the acute toxicity study. Mean body weight, food consumption, and water intake following by 14-days consecutive intraperitoneal injection at 20 and 50 mg/kg of NCK in female ICR mice were recorded (n = 8). Data represent means ± SD. (B) Individual daily changes in food consumption of female ICR mice administrated with NCK during the acute toxicity study. Mean body weight, food consumption, and water intake following by 14-days consecutive intraperitoneal injection at 20 and 50 mg/kg of NCK in female ICR mice were recorded (n = 8). Data represent means ± SD. (C) Individual daily changes in water consumption of female ICR mice administrated with NCK during the acute toxicity study. Mean body weight, food consumption, and water intake following by 14-days consecutive intraperitoneal injection at 20 and 50 mg/kg of NCK in female ICR mice were recorded (n = 8). Data represent means ± SD. (D) Changes in the organs of female ICR mice administrated

with NCK during the acute toxicity study. Comparison of relative lung, liver, spleen, heart, kidney weights (%, organ body index) and colon length at day 15. (E) Photographs of organs from the vehicle and NCK-treated mice during the MTD study. (F) Effects of NCK on serum hematological and biochemical parameters during the acute toxicity study. Abbreviations: WBC, white blood cells; RBC, red blood cell; RDW, red cell distribution width; HGB, haemoglobin; HCT, haematocrit; PLT, platelet; PDW, platelet distribution width; MCV, mean corpuscular volume; MCH, mean corpuscular haemoglobin; MCHC, mean corpuscular haemoglobin concentration; MPV, mean platelet volume; MON, monocyte; GRAN, granulocytes; LYM, lymphocyte; ALT/GOT, alanine aminotransferase/ glutamic oxaloacetic transaminase; AST/GPT, aspartate aminotransferase/ glutamic pyruvic transaminase; CK, creatine kinase; CREA, creatinine; GLU, glucose; GSP, glycated serum protein; LDH, lactate dehydrogenase; UREA, urea.

A

Code	Compound	Target	Pathway
S1002	ABT-737	Bcl-2,Autophagy	Apoptosis
S1003	Linifanib (ABT-869)	CSF-1R,PDGFR,VEGFR	Protein Tyrosine Kinase
S1004	Veliparib (ABT-888)	PARP	DNA Damage
S1005	Axitinib	c-Kit,PDGFR,VEGFR	Protein Tyrosine Kinase
S1006	Saracatinib (AZD0530)	Src	Angiogenesis
S1008	Selumetinib (AZD6244)	MEK	MAPK
S1010	Nintedanib (BIBF 1120)	FGFR,PDGFR,VEGFR	Protein Tyrosine Kinase
S1014	Bosutinib (SKI-606)	Src	Angiogenesis
S1021	Dasatinib	Bcr-Abl,c-Kit,Src	Angiogenesis
S1025	Gefitinib (ZD1839)	EGFR	Protein Tyrosine Kinase
S1029	Lenalidomide (CC-5013)	TNF-alpha	Apoptosis
S1032	Motesanib Diphosphate (AMG-706)	VEGFR,PDGFR,c-Kit	Protein Tyrosine Kinase
S1033	Nilotinib (AMN-107)	Bcr-Abl	Angiogenesis
S1036	PD0325901	MEK	MAPK
S1038	PI-103	Autophagy,DNA-PK,mTOR,PI3K	PI3K/Akt/mTOR
S1039	Rapamycin (Sirolimus)	Autophagy,mTOR	PI3K/Akt/mTOR
S1040	Sorafenib Tosylate	PDGFR,Raf,VEGFR	MAPK
S1044	Temsirolimus (CCI-779, NSC 683864)	mTOR	PI3K/Akt/mTOR
S1047	Vorinostat (SAHA, MK0683)	Autophagy,HDAC	Epigenetics
S1048	Tozasertib (VX-680, MK-0457)	Aurora Kinase	Cell Cycle
S1049	Y-27632 2HCl	Autophagy,ROCK	Cell Cycle
S1052	Elesclomol (STA-4783)	HSP (e.g. HSP90)	Cytoskeletal Signaling
S1053	Entinostat (MS-275)	HDAC	Epigenetics
S1055	Enzastaurin (LY317615)	PKC	TGF-beta/Smad
S1057	Obatoclax Mesylate (GX15-070)	Autophagy,Bcl-2	Apoptosis
S1060	Olaparib (AZD2281, Ku-0059436)	PARP	DNA Damage
S1061	Nutlin-3	E3 Ligase ,Mdm2	Apoptosis
S1065	Pictilisib (GDC-0941)	PI3K	PI3K/Akt/mTOR
S1067	SB431542	TGF-beta/Smad	TGF-beta/Smad
S1068	Crizotinib (PF-02341066)	ALK,c-Met	Protein Tyrosine Kinase
S1069	Luminespib (AUY-922, NVP-AUY922)	HSP (e.g. HSP90)	Cytoskeletal Signaling
S1070	PHA-665752	c-Met	Protein Tyrosine Kinase
S1072	ZSTK474	PI3K	PI3K/Akt/mTOR
S1075	SB216763	GSK-3	PI3K/Akt/mTOR
S1078	MK-2206 2HCl	Akt	PI3K/Akt/mTOR
S1080	SU11274	c-Met	Protein Tyrosine Kinase
S1082	Vismodegib (GDC-0449)	Hedgehog/Smoothened	Stem Cells & Wnt
S1085	Belinostat (PXD101)	HDAC	Epigenetics
S1087	Iniparib (BSI-201)	PARP	DNA Damage
S1090	Abexinostat (PCI-24781)	HDAC	Cytoskeletal Signaling
S1091	Linsitinib (OSI-906)	IGF-1R	Protein Tyrosine Kinase
S1092	KU-55933 (ATM Kinase Inhibitor)	ATM/ATR	DNA Damage
S1093	GSK1904529A	IGF-1R	Protein Tyrosine Kinase
S1094	PF-04217903	c-Met	Protein Tyrosine Kinase

S1098	Rucaparib (AG-014699,PF-01367338) phosphate	PARP	DNA Damage
S1101	Vatalanib (PTK787) 2HCl	VEGFR	Protein Tyrosine Kinase
S1104	GDC-0879	Raf	MAPK
S1105	LY294002	Autophagy,PI3K	PI3K/Akt/mTOR
S1107	Danusertib (PHA-739358)	Aurora Kinase,Bcr-Abl,c-RET,FGFR	Cell Cycle
S1109	BI 2536	PLK	Cell Cycle
S1113	GSK690693	Akt	PI3K/Akt/mTOR
S1114	JNJ-38877605	c-Met	Protein Tyrosine Kinase
S1120	Everolimus (RAD001)	mTOR	PI3K/Akt/mTOR
S1121	TW-37	Bcl-2	Apoptosis
S1122	Mocetinostat (MGCD0103)	HDAC	Epigenetics
S1129	SRT1720 HCl	Sirtuin	Epigenetics
S1130	YM155 (Sepantronium Bromide)	Survivin	Apoptosis
S1133	Alisertib (MLN8237)	Aurora Kinase	Cell Cycle
S1140	Andarine	Androgen Receptor	Endocrinology & Hormones
S1142	Alvespimycin (17-DMAG) HCl	HSP (e.g. HSP90)	Cytoskeletal Signaling
S1145	SNS-032 (BMS-387032)	CDK	Cell Cycle
S1147	Barasertib (AZD1152-HQPA)	Aurora Kinase	Cell Cycle
S1150	Paclitaxel	Autophagy,Microtubule Associated	Cytoskeletal Signaling
S1153	Roscovitine (Seliciclib,CYC202)	CDK	Cell Cycle
S1154	SNS-314	Aurora Kinase	Cell Cycle
S1156	Capecitabine	DNA/RNA Synthesis	DNA Damage
S1164	Lenvatinib (E7080)	VEGFR	Protein Tyrosine Kinase
S1165	ABT-751 (E7010)	Microtubule Associated	Cytoskeletal Signaling
S1168	Valproic acid sodium salt (Sodium valproate)	GABA Receptor,HDAC,Autophagy	Neuronal Signaling
S1171	CYC116	Aurora Kinase,VEGFR	Cell Cycle
S1172	JNJ-26854165 (Serdemetan)	E3 Ligase ,p53	Apoptosis
S1173	WZ4002	EGFR	Protein Tyrosine Kinase
S1175	BIIB021	HSP (e.g. HSP90)	Cytoskeletal Signaling
S1176	Plinabulin (NPI-2358)	VDA	Angiogenesis
S1180	XAV-939	Wnt/beta-catenin	Stem Cells & Wnt
S1181	ENMD-2076	Aurora Kinase,FLT3,VEGFR	Angiogenesis
S1186	BIBR 1532	Telomerase	DNA Damage
S1188	Anastrozole	Aromatase	Endocrinology & Hormones
S1189	Aprepitant	Substance P	Others
S1190	Bicalutamide	Androgen Receptor	Endocrinology & Hormones
S1194	CUDC-101	EGFR,HDAC,HER2	Epigenetics
S1196	Exemestane	Aromatase	Endocrinology & Hormones
S1199	Cladribine	DNA/RNA Synthesis	DNA Damage
S1200	Decitabine	DNA Methyltransferase	Epigenetics
S1207	Tivozanib (AV-951)	c-Kit,PDGFR,VEGFR	Protein Tyrosine Kinase
S1208	Doxorubicin (Adriamycin) HCl	Topoisomerase	DNA Damage
S1209	Fluorouracil (5-Fluoracil, 5-FU)	DNA/RNA Synthesis	DNA Damage
S1210	Methotrexate	DHFR	Metabolism
S1218	Clofarabine	DNA/RNA Synthesis	DNA Damage
S1219	YM201636	PI3K	PI3K/Akt/mTOR

S1220	OSI-930	c-Kit,CSF-1R,VEGFR	Protein Tyrosine Kinase
S1225	Etoposide	Topoisomerase	DNA Damage
S1226	KU-0063794	mTOR	PI3K/Akt/mTOR
S1227	Raloxifene HCl	Estrogen/progestogen Receptor	Endocrinology & Hormones
S1231	Topotecan HCl	Topoisomerase	DNA Damage
S1233	2-Methoxyestradiol (2-MeOE2)	HIF	Angiogenesis
S1235	Letrozole	Aromatase	Endocrinology & Hormones
S1237	Temozolomide	DNA/RNA Synthesis,Autophagy	DNA Damage
S1241	Vincristine sulfate	Autophagy,Microtubule Associated	Cytoskeletal Signaling
S1244	Amuvatinib (MP-470)	c-Kit,FLT3,PDGFR	Protein Tyrosine Kinase
S1249	JNJ-7706621	Aurora Kinase,CDK	Cell Cycle
S1250	Enzalutamide (MDV3100)	Androgen Receptor	Endocrinology & Hormones
S1261	Celecoxib	COX	Neuronal Signaling
S1264	PD173074	FGFR,VEGFR	Angiogenesis
S1266	WYE-354	mTOR	PI3K/Akt/mTOR
S1267	Vemurafenib (PLX4032, RG7204)	Raf	MAPK
S1274	BX-795	IκB/IKK,PDK	PI3K/Akt/mTOR
S1278	Altretamine	DNA alkylator	DNA Damage
S1304	Megestrol Acetate	Androgen Receptor, Estrogen/progestogen Receptor	Endocrinology & Hormones
S1322	Dexamethasone (DHAP)	Autophagy,IL Receptor	Others
S1369	Bafetinib (INNO-406)	Bcr-Abl	Angiogenesis
S1378	Ruxolitinib (INCB018424)	JAK	JAK/STAT
S1392	Pelitinib (EKB-569)	EGFR	Protein Tyrosine Kinase
S1443	Zileuton	Lipoxygenase	Metabolism
S1452	Ispinesib (SB-715992)	Kinesin	Cytoskeletal Signaling
S1453	Tipifarnib	Transferase	Metabolism
S1456	Zibotentan (ZD4054)	Endothelin Receptor	GPCR & G Protein
S1467	Doxercalciferol	Vitamin	Metabolism
S1476	SB525334	TGF-beta/Smad	TGF-beta/Smad
S1486	AEE788 (NVP-AEE788)	EGFR,HER2,VEGFR	Protein Tyrosine Kinase
S1487	PHA-793887	CDK	Cell Cycle
S1489	PIK-93	PI3K	PI3K/Akt/mTOR
S1490	Ponatinib (AP24534)	BcrAbl,FGFR,PDGFR, VEGFR	Angiogenesis
S1501	Mycophenolate Mofetil	Dehydrogenase	Metabolism
S1515	Pracinostat (SB939)	HDAC	Cytoskeletal Signaling
S1525	Adavosertib （MK-1775）	Wee1	Cell Cycle
S1526	Quizartinib (AC220)	FLT3	Angiogenesis
S1532	AZD7762	Chk	Cell Cycle
S1533	R406 (free base)	Syk	Angiogenesis
S1541	Selisistat (EX 527)	Sirtuin	Epigenetics
S1547	Febuxostat	ROS	Immunology & Inflammation
S1548	Dapagliflozin	SGLT	GPCR & G Protein
S1555	AZD8055	mTOR	PI3K/Akt/mTOR
S1561	BMS-777607	TAM Receptor,c-Met	Protein Tyrosine Kinase
S1570	KU-60019	ATM/ATR	DNA Damage
S1574	Doramapimod (BIRB 796)	p38 MAPK	MAPK

S1577	Tie2 kinase inhibitor	Tie-2	Protein Tyrosine Kinase
S1655	Ezetimibe	LDL	Metabolism
S1665	Estrone	Estrogen/progestogen Receptor	Endocrinology & Hormones
S1672	Aminoglutethimide	Aromatase	Endocrinology & Hormones
S1680	Disulfiram	Dehydrogenase	Metabolism
S1696	Hydrocortisone	Glucocorticoid Receptor	Endocrinology & Hormones
S1709	Estradiol	Estrogen/progestogen Receptor	Endocrinology & Hormones
S1721	Azathioprine	Rho	Cell Cycle
S1776	Toremifene Citrate	Estrogen/progestogen Receptor	Endocrinology & Hormones
S1782	Azacitidine	DNA Methyltransferase	DNA Damage
S1792	Simvastatin	HMG-CoA Reductase	Metabolism
S1840	Lomustine	DNA/RNA Synthesis	DNA Damage
S1908	Flutamide	Androgen Receptor	Endocrinology & Hormones
S1909	Fluvastatin Sodium	HMG-CoA Reductase	Metabolism
S1972	Tamoxifen Citrate	Estrogen/progestogen Receptor,Autophagy	Endocrinology & Hormones
S2003	Maraviroc	CCR	Microbiology
S2013	PF-573228	FAK	Angiogenesis
S2057	Cyclophosphamide Monohydrate	DNA alkylator	DNA Damage
S2151	Sonidegib (Erismodegib, NVP-LDE225)	Hedgehog/Smoothened	Stem Cells & Wnt
S2163	PF-4708671	S6 Kinase	PI3K/Akt/mTOR
S2178	AG-14361	PARP	DNA Damage
S2181	Ixazomib Citrate (MLN9708)	Proteasome	Proteases
S2193	GSK461364	PLK	Cell Cycle
S2198	SGI-1776 free base	Pim	JAK/STAT
S2201	BMS-794833	c-Met,VEGFR	Protein Tyrosine Kinase
S2208	Formestane	Aromatase	Endocrinology & Hormones
S2215	DAPT (GSI-IX)	Beta Amyloid,Gamma-secretase	Proteases
S2217	Irinotecan HCl Trihydrate	Topoisomerase	DNA Damage
S2219	Momelotinib (CYT387)	JAK	JAK/STAT
S2220	SB590885	Raf	MAPK
S2225	TAME	APC,E3 Ligase	Cell Cycle
S2226	Idelalisib (CAL-101, GS-1101)	PI3K	PI3K/Akt/mTOR
S2230	Galunisertib (LY2157299)	TGF-beta/Smad	TGF-beta/Smad
S2231	Telatinib	c-Kit,PDGFR,VEGFR	Protein Tyrosine Kinase
S2235	Volasertib (BI 6727)	PLK	Cell Cycle
S2243	Degrasyn (WP1130)	Bcr-Abl,DUB	Angiogenesis
S2247	Buparlisib (BKM120, NVP-BKM120)	PI3K	PI3K/Akt/mTOR
S2248	Silmitasertib (CX-4945)	Casein Kinase	Metabolism
S2250	(-)-Epigallocatechin Gallate	DNA Methyltransferase, HER2, Telomerase, EGFR,Fatty Acid Synthase	DNA Damage
S2286	Cyclosporin A	Immunology & Inflammation related	Immunology & Inflammation
S2303	gossypol-Acetic acid	Dehydrogenase	Metabolism
S2342	Phloretin	SGLT	GPCR & G Protein
S2406	Chrysophanic Acid	EGFR,mTOR	Protein Tyrosine Kinase
S2485	Mitoxantrone 2HCl	Topoisomerase	DNA Damage
S2487	Mycophenolic acid	Dehydrogenase	Metabolism
S2606	Mifepristone	Estrogen/progestogen Receptor	Endocrinology & Hormones

S2610	Lonidamine	Others	Others
S2617	TAK-733	MEK	MAPK
S2626	Rabusertib (LY2603618)	Chk	Cell Cycle
S2630	GW3965 HCl	Liver X Receptor	Others
S2634	Rebastinib (DCC-2036)	Bcr-Abl	Angiogenesis
S2658	Omipalisib (GSK2126458, GSK458)	mTOR,PI3K	PI3K/Akt/mTOR
S2660	MK-0752	Beta Amyloid,Gamma-secretase	Proteases
S2666	PF-3845	FAAH	Metabolism
S2673	Trametinib (GSK1120212)	MEK	MAPK
S2679	Flavopiridol HCl	CDK	Cell Cycle
S2680	Ibrutinib (PCI-32765)	BTK	Angiogenesis
S2686	NVP-BSK805 2HCl	JAK	JAK/STAT
S2694	Turofexorate Isopropyl (XL335)	FXR	Others
S2697	A-769662	AMPK,Fatty Acid Synthase	PI3K/Akt/mTOR
S2699	CH5132799	PI3K	PI3K/Akt/mTOR
S2700	KX2-391	Src	Angiogenesis
S2711	Dibenzazepine (YO-01027)	Gamma-secretase	Proteases
S2719	AMG-900	Aurora Kinase	Cell Cycle
S2726	PH-797804	p38 MAPK	MAPK
S2727	Dacomitinib (PF299804, PF299)	EGFR	Protein Tyrosine Kinase
S2730	Crenolanib (CP-868596)	PDGFR	Protein Tyrosine Kinase
S2731	AZ 3146	Kinesin	Cytoskeletal Signaling
S2736	Fedratinib (SAR302503, TG101348)	JAK	JAK/STAT
S2738	PAC-1	Caspase	Apoptosis
S2746	AZ 628	Raf	MAPK
S2760	Canagliflozin	SGLT	GPCR & G Protein
S2767	3-Methyladenine (3-MA)	Autophagy,PI3K	PI3K/Akt/mTOR
S2772	Dalcetrapib (JTT-705, RO4607381)	CETP	Metabolism
S2775	Nocodazole	Autophagy,Microtubule Associated	Cytoskeletal Signaling
S2782	GW4064	FXR	Others
S2789	Tofacitinib (CP-690550,Tasocitinib)	JAK	JAK/STAT
S2791	Sotrastaurin	PKC	TGF-beta/Smad
S2804	Sirtinol	Sirtuin	Epigenetics
S2806	CEP-33779	JAK	JAK/STAT
S2811	Sapanisertib (INK 128, MLN0128)	mTOR	PI3K/Akt/mTOR
S2817	Torin 2	ATM/ATR,mTOR	PI3K/Akt/mTOR
S2821	RG108	DNA Methyltransferase	Epigenetics
S2824	TPCA-1	IκB/IKK	NF-κB
S2890	PF-562271	FAK	Angiogenesis
S2902	S-Ruxolitinib (INCB018424)	JAK	JAK/STAT
S2913	BAY 11-7082	E2 conjugating,IκB/IKK	NF-κB
S3035	Daunorubicin HCl	Topoisomerase	DNA Damage
S3172	Anagrelide HCl	PDE	Metabolism
S3604	Triptolide (PG490)	NF-κB	NF-κB
S4125	Sodium Phenylbutyrate	HDAC	DNA Damage
S4902	QNZ (EVP4593)	NF-κB,TNF-alpha	NF-κB
S5002	Fingolimod (FTY720) HCl	S1P Receptor	GPCR & G Protein
S7092	SANT-1	Hedgehog/Smoothened	Stem Cells & Wnt

S7114	NU6027	CDK	Cell Cycle
S7176	SKI II	S1P Receptor	GPCR & G Protein
S7209	GSK650394	Others	Others
S7352	Bay 11-7085	IκB/IKK	NF-κB
S7435	AR-A014418	GSK-3	PI3K/Akt/mTOR
S7476	MG149	Histone Acetyltransferase	Epigenetics
S7524	FR 180204	ERK	MAPK
S7685	SecinH3	Others	Others
S7783	Combretastatin A4	Microtubule Associated	Cytoskeletal Signaling
S8005	SMI-4a	Pim	JAK/STAT
S8006	BIX 01294	Histone Methyltransferase	Epigenetics
S8014	GW9508	GPR	Endocrinology & Hormones
S8025	GSK3787	PPAR	Metabolism
S8031	NSC 23766	Rho	Cell Cycle
S8037	Necrostatin-1	TNF-alpha	Apoptosis
S8056	Lomeguatrib	Transferase	Metabolism
S1224	Oxaliplatin	DNA/RNA Synthesis	DNA Damage

B

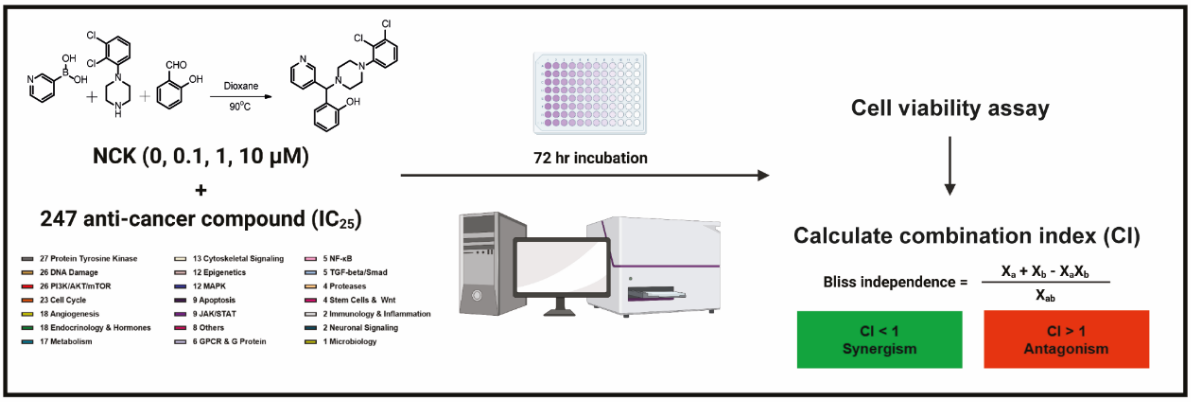

Supplementary Figure 11: (A) Target and pathway information of 247 anti-cancer compounds of Cambridge anti-cancer compound library. (B) Schematic of anti-cancer compound library screen. 247 anti-cancer agents (compound X) were combined with NCK to treat MDA-MB-231. Cell viability was determined 72-hour post-treatment with AlamarBlue viability assay.

A

TKIs	Main targets (IC_{50}, nM)	Other targets (IC_{50}, nM)	Clinical Phase
**OSI-930**	VEGFR2 (9), CSF-1R (15), c-Kit (80)	Flt1 (8), LCK (22), c-Raf (41)	I
**Tie2 kinase inhibitor**	Tie-2 (0.25)	-	Pre-clinical
**Crizotinib**	c-Met (11), ALK (24)	ROS1 (Ki<0.025)	FDA approved
**Pelitinib**	EGFR (38.5)	Src (282), MEK/ERK (800), ErbB2 (1255)	II
**Sorafenib**	Raf1 (6), Braf (22), VEGFR3 (20), PDGFR-β (57), VEGFR2 (90)	Flt-3 (59), c-KIT (68)	FDA approved
**Ponatinib**	Bcr-Abl (0.37), PDGFRα (1.1), VEGFR2 (1.5), FGFR1 (2.2)	Src (5.4)	FDA approved
**BMS-794833**	c-Met (1.7), VEGFR2 (15)	Ron (<3), Axl (<3), Flt3 (<3)	I

B

Compound	Target	Dosage （FDA/Clinical Trial）	Side Effects/Toxicity
**OSI-930**	c-Kit<br>VEGFR2<br>FLT-1<br>CSF-1R<br>c-RAF<br>LCK<br>PDGFR	Clinical Trial<br>NCT00513851 (Phase I):<br>MTD- 1600mg QD/ 500mg BID<br>NCT00603356 (Phase I):<br>MTD- 200 mg OSI-930 BID+150 mg erlotinib QD	Fatigue<br>Anorexia<br>Nausea<br>Diarrhoea<br>Rash<br>Lethargy<br>Hypertension<br>Skin Toxicity
**Crizotinib**	c-Met<br>ALK<br>ROS1	FDA<br>250mg BID	Hepatotoxicity<br>Interstitial Lung Disease (Pneumonitis)<br>QT Interval Prolongation<br>Bradycardia<br>Severe Visual Loss<br>Gastrointestinal Toxicity<br>Embryo-Fetal Toxicity<br>Renal Impairment<br>Tiredness<br>Arms/Legs numbness or tingling

Supplementary Figure 12: (A) Targets of the synergistic TKIs, IC_{50} of the inhibitors on corresponding targets and their clinical status (SelleckChem). (B) Information of OSI-930 and Crizotinib, and associated side effects and toxicities in FDA/clinical trials.

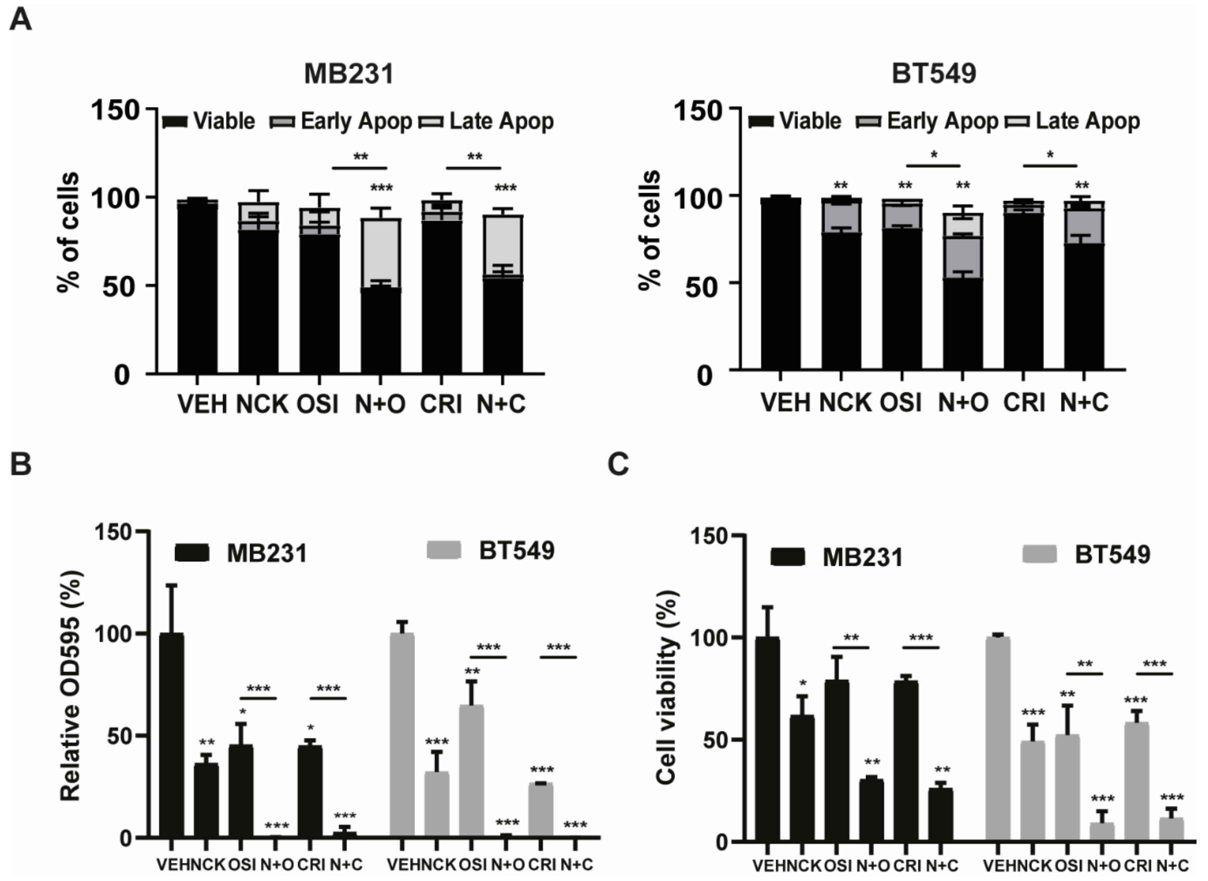

Supplementary Figure 13: (A) Quantification of annexin-V and propidium iodide (PI) stained apoptotic cell death of MDA-MB-231 cells and BT549 cells measured after treatment with 5 μM NCK, 5 μM OSI-930 (OSI) and 5 μM Crizotinib (CRI) or combinations using flow cytometry analysis. Data represent means ± SD (n=3). *$P < 0.05$, **$P < 0.01$, and ***$P < 0.001$. (B) Crystal violet staining of foci in colonies generated by MDA-MB-231 cells after exposure to 5 μM NCK, 5 μM OSI-930 (OSI) and 5 μM Crizotinib (CRI) or combinations. Cell viability was measured by eluting the crystal violet with methanol and detect absorbance at 595 nm using microplate reader (Tecan Spark®, Switzerland). Data represent means ± SD (n=3). *$P <$ 0.05, **$P < 0.01$, and ***$P < 0.001$. (C) Cell viability of colonies generated by MDA-MB-231 cells and BT549 cells in 3D Matrigel after exposure to 5 μM NCK, 5 μM OSI-930 (OSI) and 5 μM Crizotinib (CRI) or combinations were determined using the AlamarBlue viability assay. Data represent means ± SD (n=3). *$P < 0.05$, **$P < 0.01$, and ***$P < 0.001$.

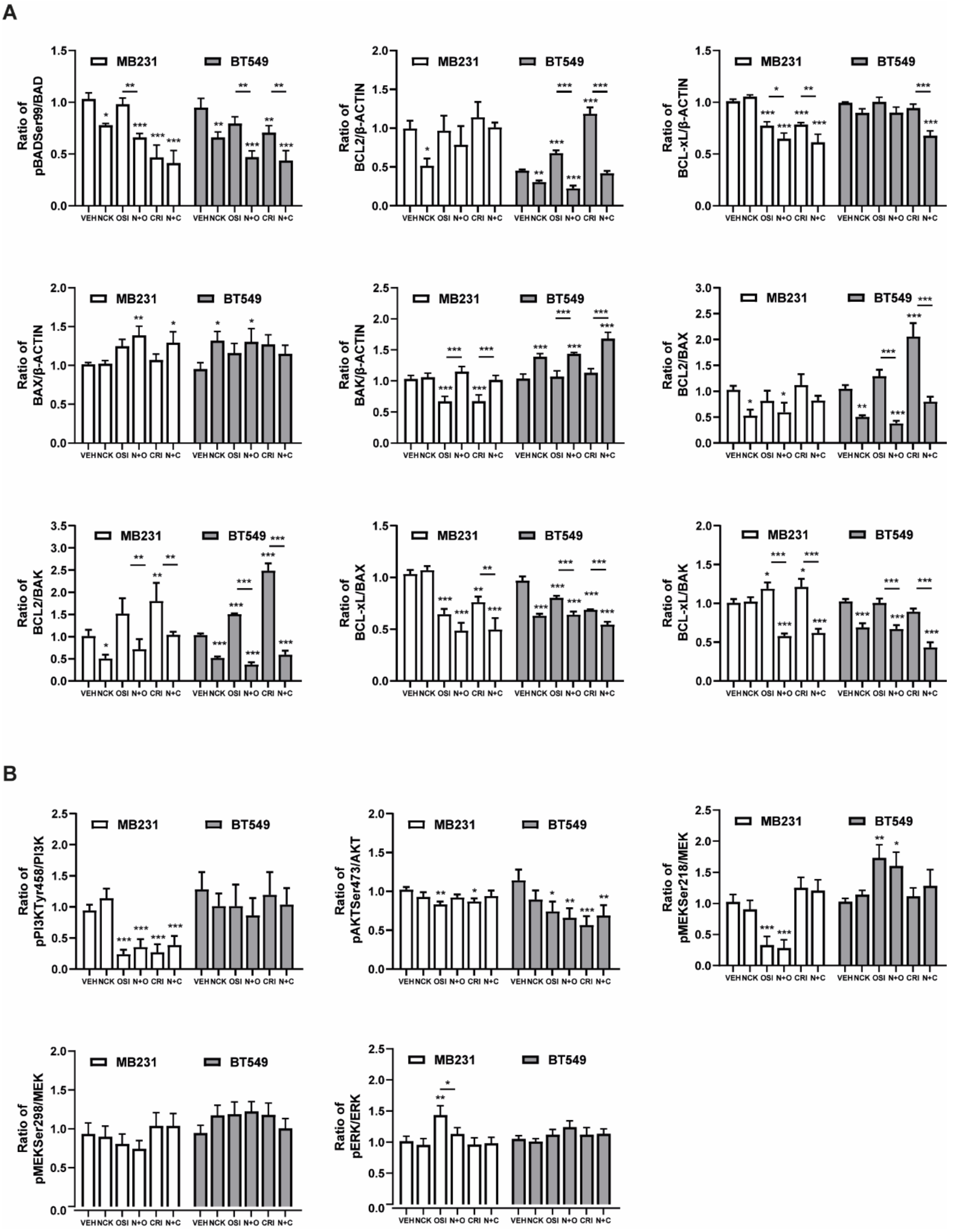

Supplementary Figure 14: (A) Densitometric analysis of western blots in Figure 7E. MDA-MB-231 and BT549 cells were treated with 1 μM NCK, 1 μM OSI-930 (OSI) and 1 μM Crizotinib (CRI) or combinations (n=3). Densitometric analysis of protein blots was determined using ImageJ software (https://imagej.nih.gov/ij/). Statistical changes were assessed by using ANOVA. *$P < 0.05$, **$P < 0.01$, and ***$P < 0.001$. (B) Densitometric analysis of western blots in Figure 7F. MDA-MB-231 and BT549 cells were treated with 1 μM

NCK, 1 μM OSI-930 (OSI) and 1 μM Crizotinib (CRI) or combinations (n=3). Densitometric analysis of protein blots was determined using ImageJ software (https://imagej.nih.gov/ij/). Statistical changes were assessed by using ANOVA. *$P < 0.05$, **$P < 0.01$, and ***$P < 0.001$.

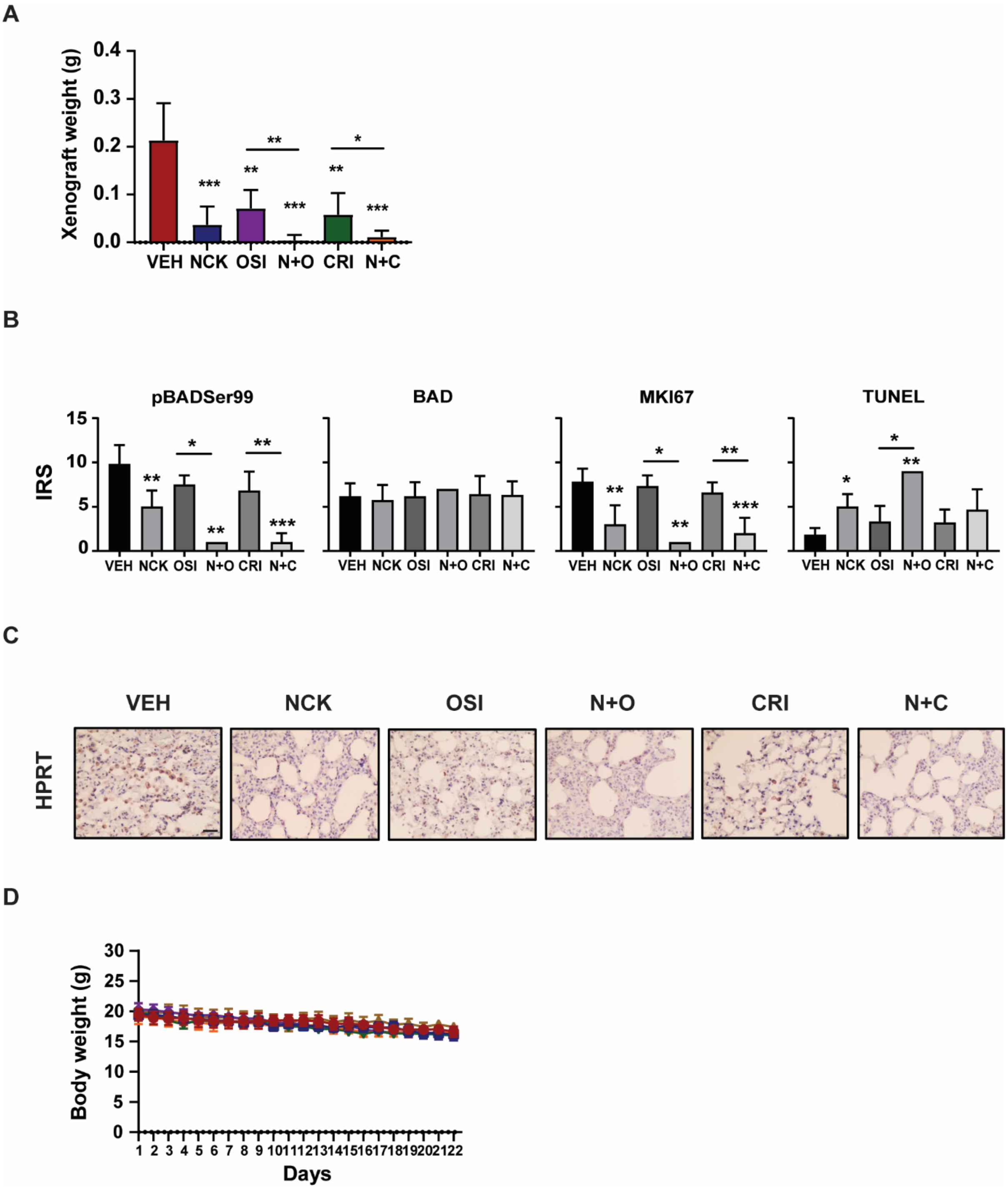

E

	Vehicle	NCK (20 mg/kg)	OSI (50 mg/kg)	NCK+OSI	CRI (50 mg/kg)	NCK+CRI	*P*-value
**ALT/GOT (U/L)**	75.68±19.88	75.66±23.55	81.99±27.99	76.27±37.43	74.65±13.57	88.40±21.76	0.913
**AST/GPT (U/L)**	295.82±95.02	296.04±102.48	298.22±75.62	273.64±54.25	303.05±84.19	337.71±95.45	0.946
**CREA (μmol/L)**	23.62±1.37	23.49±3.02	24.89±9.0.7	23.84±7.43	29.47±6.16	26.06±2.52	0.548
**UREA (mmol/L)**	11.76±2.42	14.01±3.33	12.36±6.01	13.14±6.70	17.47±4.52	16.74±3.79	0.318
**ALB (g/L)**	29.08±3.78	28.48±3.45	30.61±0.84	30.74±4.28	30.53±0.88	28.76±2.33	0.681

F

	Vehicle	NCK (20 mg/kg)	OSI (50 mg/kg)	NCK+OSI	CRI (50 mg/kg)	NCK+CRI	*P*-value
**Relative Lung Weight (%)**	2.80±0.60	2.39±0.57	2.28±0.51	1.98±0.22	2.16±0.19	2.41±0.51	0.090
**Relative Liver Weight (%)**	5.72±0.18	5.70±0.38	5.53±0.81	4.84±0.30	5.49±0.70	5.00±0.38	<0.05
**Relative Spleen Weight (%)**	1.27±0.33	0.77±0.14**	0.75±0.27*	0.56±0.07***	0.74±0.24*	0.55±0.12***	<0.001
**Relative Heart Weight (%)**	0.69±0.07	0.74±0.16	0.68±0.13	0.64±0.03	0.78±0.09	0.76±0.16	0.308
**Relative Kidney Weight (%)**	1.67±0.10	1.60±0.14	1.63±0.16	1.55±0.11	1.70±0.11	1.63±0.11	0.380
**Colon Length (cm)**	8.88±0.25	9.55±0.84	9.34±0.67	8.98±0.18	8.45±0.39	8.53±0.29	<0.005

Data represent means ± SD. **P* < 0.05, ***P* < 0.01, and ****P* < 0.001.

Supplementary Figure 15: (A) Mean MDA-MB-231 xenograft weight of each treatment group after sacrifice at the end of 21st day. Data represent means ± SD (n=6). *P < 0.05, **P < 0.01, and ***P < 0.001. (B) IRS scoring of pBAD at Ser99, BAD, MKI67 and TUNEL staining in MDA-MB-231 xenografts. IRS scoring method is described in materials & methods. (C) IHC images of TUNEL staining in xenografts. Scale bar, 20 µm. (D) Animal weight of each treatment group. (E) Effects of NCK, OSI-930 (OSI) and Crizotinib (CRI) or combinations on serum biochemical parameters. Abbreviations: ALT/GOT, alanine aminotransferase/ glutamic oxaloacetic transaminase; AST/GPT, aspartate aminotransferase/ glutamic pyruvic transaminase; CREA, creatinine; UREA, urea; and ALB, albumin. Data represent means ± SD. (F) Changes in relative lung, liver, spleen, heart, kidney weights (%, organ body index) and colon length of Balb/c-nude mice administrated with NCK, OSI-930 (OSI) and Crizotinib (CRI) or combinations during the combination study at day 22. Data represent means ± SD. *P < 0.05, **P < 0.01, and ***P < 0.001.

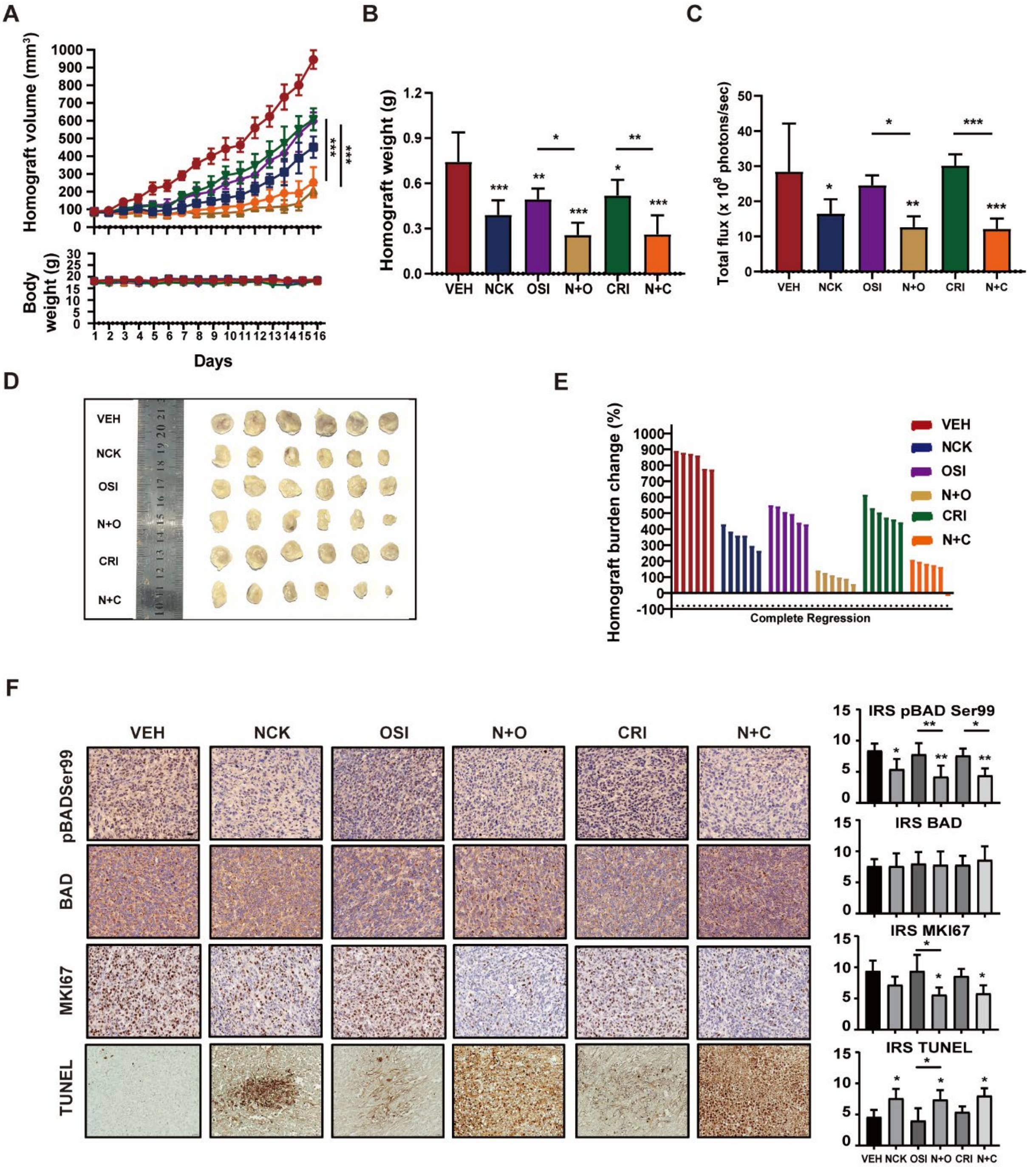

G

	Vehicle	NCK (20 mg/kg)	OSI (50 mg/kg)	NCK+OSI	CRI (50 mg/kg)	NCK+CRI	*P*-value
**Relative Lung Weight (%)**	1.06±0.15	0.87±0.11	0.89±0.19	0.83±0.23	0.96±0.18	0.87±0.27	0.397
**Relative Liver Weight (%)**	5.40±0.30	4.98±0.21	5.32±0.32	5.10±0.40	5.54±0.23	5.38±0.22	<0.05
**Relative Spleen Weight (%)**	3.42±0.60	2.24±0.40**	2.75±0.55	1.33±0.32*** (***compared to OSI)	2.75±0.26	1.65±0.54*** (**compared to CRI)	<0.001
**Relative Heart Weight (%)**	0.61±0.09	0.61±0.11	0.49±0.05	0.61±0.12	0.66±0.20	0.65±0.16	0.329
**Relative Kidney Weight (%)**	1.48±0.04	1.40±0.16	1.34±0.02	1.32±0.21	1.41±0.07	1.48±0.18	0.203
**Colon Length (cm)**	10.20±1.84	9.02±1.54	9.33±1.26	8.92±0.75	9.50±1.97	10.15±0.58	0.496

Data represent means ± SD. *$P < 0.05$, **$P < 0.01$, and ***$P < 0.001$.

Supplementary Figure 16: (A) Homograft volume (mm^3) of a 4T1 orthotopic syngeneic model was measured every day and calculated by using the formula: $0.52 \times \text{length} \times [\text{width}]^2$. The animal weight of each treatment group is indicated. (B) Mean homograft weight of each treatment group after sacrifice on the 15th day of treatment. (C) Bioluminescence of homografts from mice orthotopically implanted with 4T1-luciferase cells. (D) Resected homografts from each treatment group. (E) Homograft burden change of each treatment group. (F) Histological analyses and IRS scoring of pBAD at Ser99, BAD, MKI67, and TUNEL staining in homografts. Scale bar, 20 μm. (G) Changes in relative lung, liver, spleen, heart, kidney weights (%, organ body index) and colon length of BALB/c mice administrated with NCK, OSI-930 (OSI) and Crizotinib (CRI) or combinations during the study at day 16. Data represent means ± SD (n=6). $^*P < 0.05$, $^{**}P < 0.01$, and $^{***}P < 0.001$.

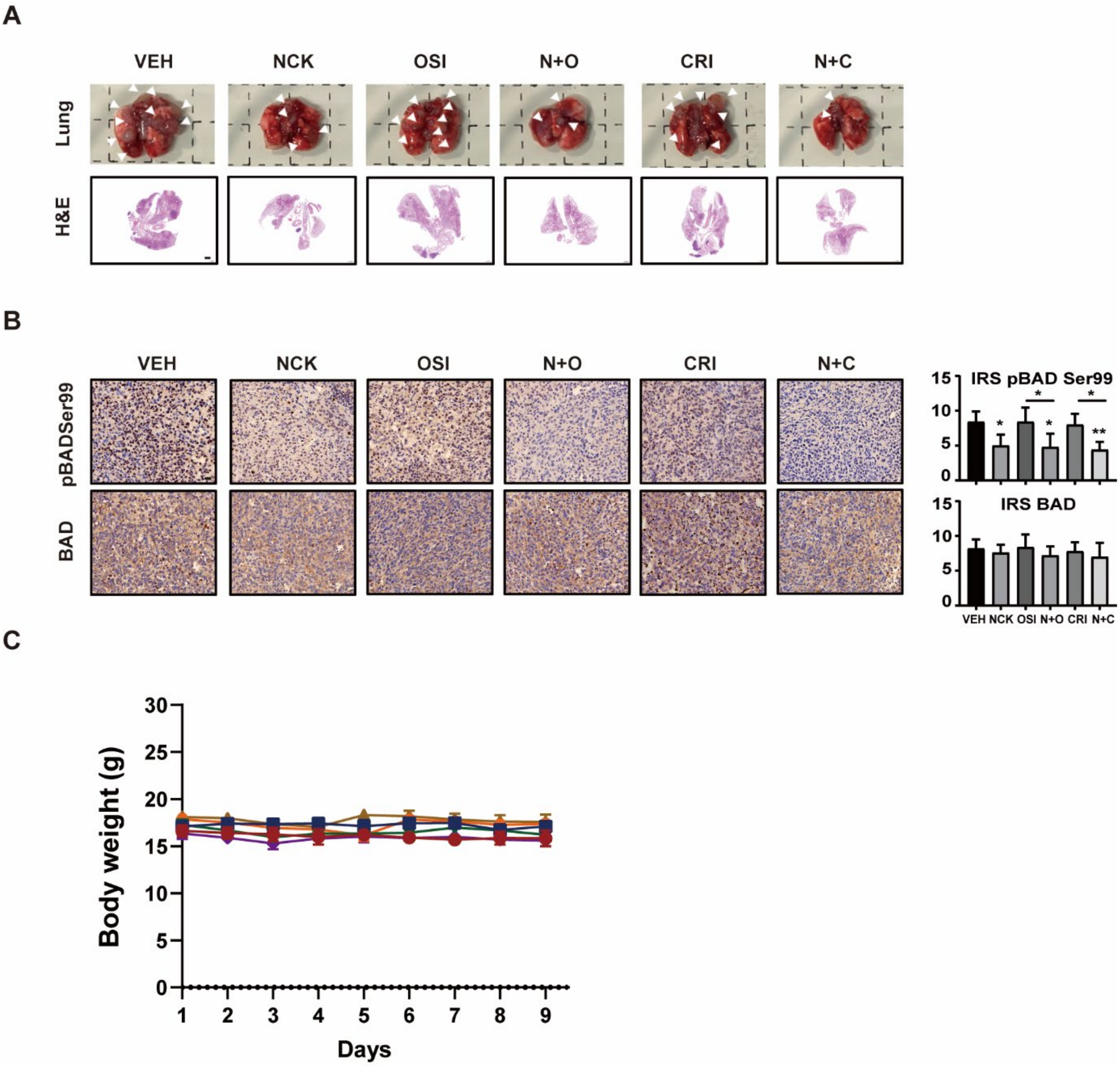

D

	Vehicle	NCK (20 mg/kg)	OSI (50 mg/kg)	NCK+OSI	CRI (50 mg/kg)	NCK+CRI	*P*-value
**Relative Lung Weight (%)**	3.27±0.74	1.70±0.49*	2.97±1.26	1.37±0.63** (* compared to OSI)	2.09±0.86	1.31±0.50**	< 0.001
**Relative Liver Weight (%)**	4.07±0.56	4.55±0.21	4.38±0.36	4.12±0.29	4.64±0.37	4.58±0.20	<0.05
**Relative Spleen Weight (%)**	1.86±0.41	1.54±0.54	2.12±0.67	1.35±0.50	1.48±0.66	1.16±0.43	0.060
**Relative Heart Weight (%)**	0.55±0.11	0.51±0.07	0.61±0.11	0.51±0.03	0.58±0.11	0.55±0.06	0.372
**Relative Kidney Weight (%)**	1.53±0.14	1.40±0.07	1.44±0.10	1.49±0.25	1.59±0.07	1.49±0.15	0.285

Data represent means ± SD. *$P < 0.05$, **$P < 0.01$, and ***$P < 0.001$.

Supplementary Figure 17: (A) Representative images and H&E staining images of the lungs of 4T1-luciferase cell generated metastasis model. Scale bar, 1000 µm. (B) Histological analysis and IRS scoring of pBAD at Ser99 and BAD staining in lung sections of 4T1-

luciferase cell generated metastasis model. Scale bar, 20 μm. (C) Animal weight of each treatment group was indicated. (D) Changes in relative lung, liver, spleen, heart and kidney weights (%, organ body index) of BALB/c mice treated with NCK, OSI-930 (OSI), Crizotinib (CRI) or combinations during the study at day 9. Data represent means ± SD (n=6). *$P < 0.05$, **$P < 0.01$, and ***$P < 0.001$.

A

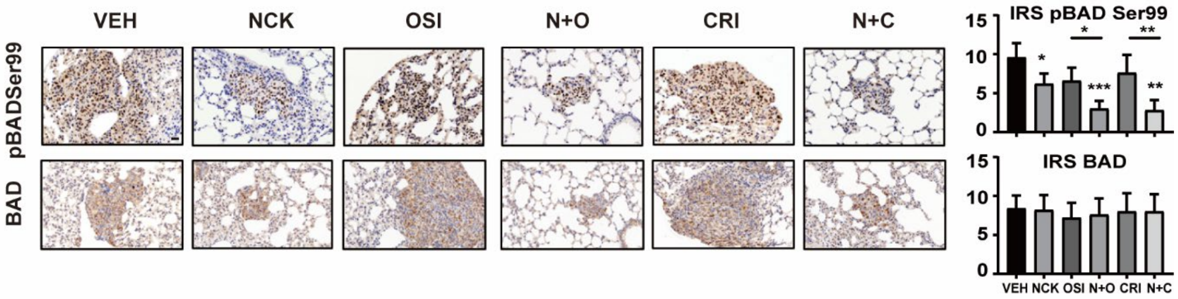

B

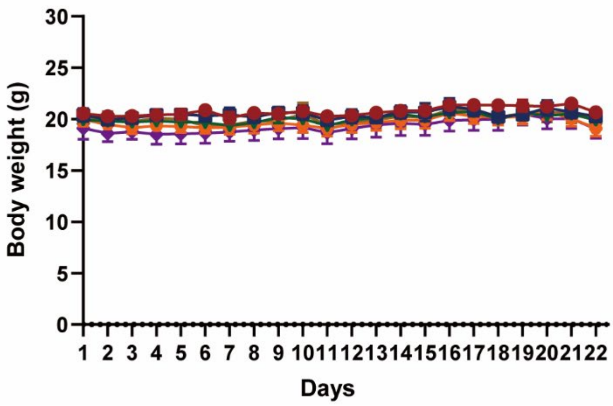

C

	Vehicle	NCK (20 mg/kg)	OSI (50 mg/kg)	NCK+OSI	CRI (50 mg/kg)	NCK+CRI	*P*-value
**Relative Lung Weight (%)**	0.95±0.11	0.82±0.05*	0.85±0.06	0.70±0.03*** (** compared to OSI)	0.86±0.05	0.78±0.08**	<0.001
**Relative Liver Weight (%)**	7.10±0.32	6.57±0.93	6.34±0.60	6.24±0.47	6.16±0.46	6.31±0.56	0.095
**Relative Spleen Weight (%)**	0.74±0.20	0.75±0.13	0.73±0.14	0.81±0.14	0.59±0.14	0.69±0.15	0.247
**Relative Heart Weight (%)**	0.68±0.05	0.66±0.08	0.66±0.07	0.64±0.09	0.62±0.05	0.69±0.07	0.559
**Relative Kidney Weight (%)**	1.76±0.09	1.72±0.18	1.73±0.15	1.78±0.09	1.78±0.05	1.71±0.15	0.851

Data represent means ± SD. $*P < 0.05$, $**P < 0.01$, and $***P < 0.001$.

Supplementary Figure 18: (A) Histological analyses and IRS scoring of pBAD at Ser99 and BAD staining in lung sections of MDA-MB-231 cell generated metastasis model. Scale bar, 20 µm. (B) Animal weight of BALB/c-nude mice intravenously injected in tail vein with MDA-MB-231 cells. The mice were treated with NCK, OSI-930 (OSI) and Crizotinib (CRI) or combinations. (C) Changes in relative lung, liver, spleen, heart and kidney weights (%, organ body index) of BALB/c-nude mice administrated with NCK, OSI-930 (OSI) and Crizotinib (CRI) or combinations during the study at day 22. Data represent means ± SD (n=6). $*P < 0.05$, $**P < 0.01$, and $***P < 0.001$.

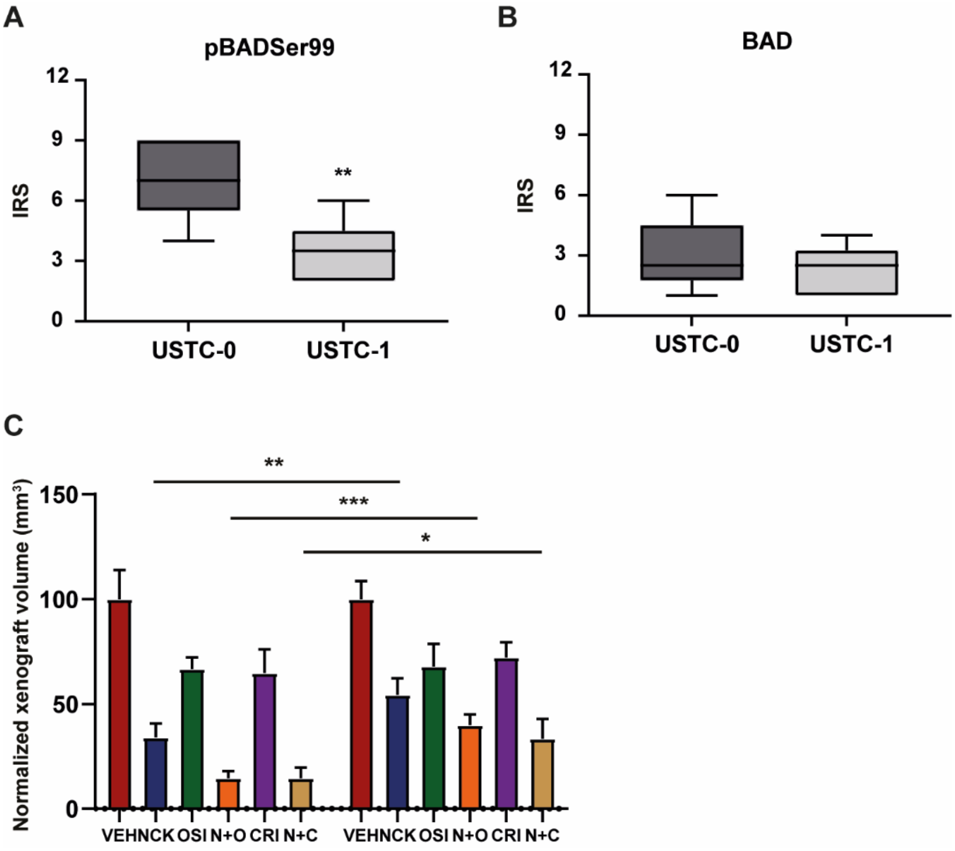

Supplementary Figure 19: (A) pBADSer99 was detected using immunohistochemistry (IHC) in USTC-0 and USTC-1. Analysis of pBADSer99 expression in USTC-0 and USTC-1 by immunoreactive score (IRS) were performed. Data represent means ± SD. **P* < 0.05, ***P* < 0.01, and ****P* < 0.001. (B) BAD expression was detected using immunohistochemistry (IHC) in USTC-0 and USTC-1. Analysis of BAD expression in USTC-0 and USTC-1 by immunoreactive score (IRS) were performed. Data represent means ± SD. **P* < 0.05, ***P* < 0.01, and ****P* < 0.001. (C) Normalized USTC-0 and USTC-1 xenograft volume of each treatment group at the end of 21st day. Data represent means ± SD (n=6). **P* < 0.05, ***P* < 0.01, and ****P* < 0.001.

Name	Sequence (5’ →3’)
**hBAD si-1 sense**	GCUCACUACCAAAUGUUAATT
**hBAD si-1 antisense**	UUAACAUUUGGUAGUGAGCAC
**hBAD si-2 sense**	GGAGGAUGAGUGACGAGUUTT
**hBAD si-2 antisense**	AACUCGUCACUCAUCCUCCGG

Supplementary Figure 20: Sequence of siRNA-BAD.

A

Antibody	Assays	Company	Catalog no.	Dilution
pBAD (Ser136)	WB	Cell Signaling Technology	4366	1:1000
pBAD (Ser136)	IHC	GeneTex	GTX50136	1:50
pBAD (Ser112)	WB	Cell Signaling Technology	9291	1:1000
pBAD (Ser155)	WB	Cell Signaling Technology	9297	1:1000
BAD	WB	Cell Signaling Technology	9268	1:1000
BAD	IHC	Abcam	ab32445	1:500
Ki67	IHC	Cell Signaling Technology	9449	1:200
Ki67	IHC	Servicebio	GB111141	1:500
HPRT	IHC	Invitrogen	PA582244	1:500
p-PI3K (Tyr458)	WB	Cell Signaling Technology	17366	1:1000
PI3K P85α	WB	Cell Signaling Technology	13666	1:1000
PI3K P110α	WB	Cell Signaling Technology	4249	1:1000
pAKT (Ser473)	WB	Cell Signaling Technology	4060	1:1000
AKT	WB	Cell Signaling Technology	4685	1:1000
pERK1/2 (Thr202/Tyr204)	WB	Cell Signaling Technology	4370	1:1000
ERK1/2	WB	Cell Signaling Technology	4695	1:1000
p-MEK1/2 (Ser218/222)	WB	Abcam	194754	1:1000
pMEK1 (Ser298)	WB	Abcam	96379	1:1000
MEK1/2	WB	Cell Signaling Technology	4694	1:1000
BCL2	WB	Cell Signaling Technology	15071	1:1000
BCLxL	WB	Cell Signaling Technology	2764	1:1000
BAX	WB	Cell Signaling Technology	5023	1:1000
BAK	WB	Cell Signaling Technology	12105	1:1000
β-ACTIN	WB	Santa Cruz	SC-47778	1:2000

Abbreviations: WB-Western blot; IHC-Immunohistochemistry

B

Gene		Forward primer	Reverse primer
**Metastasis related genes**			
**1**	*hHPRT*	5′-TTCCTTGGTCAGGCAGTATAATCC-3′	5′-AGTCTGGCTTATATCCAACACTTCG-3′
**2**	*mgapdh*	5′-CTCACTCAAGATTGTCAGCAATG-3′	5′-CACATTGGGGGTAGGAACAC-3′

Supplementary Figure 21: (A) Antibodies used for western blot (WB) and immunohistochemistry (IHC). (B) Oligonucleotide primers used for qPCR

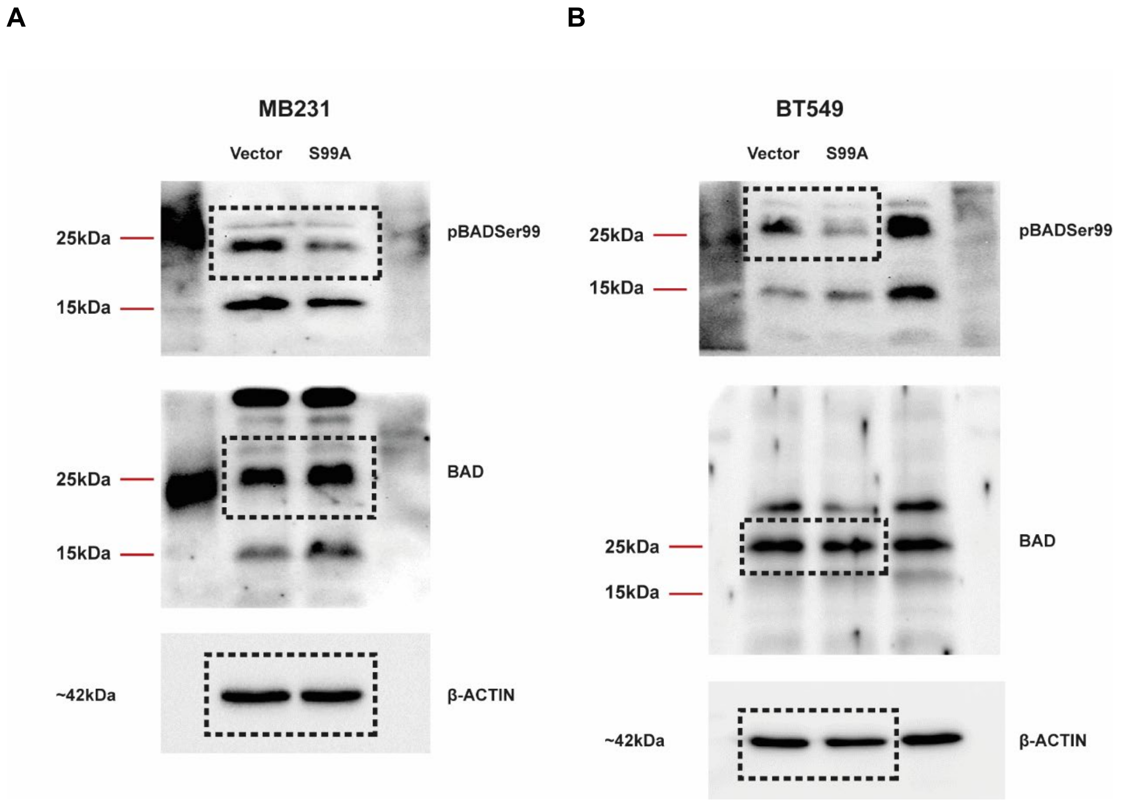
A
MB231
Vector
S99A
25kDa
15kDa
pBADSer99
25kDa
15kDa
BAD
~42kDa
β-ACTIN
B
BT549
Vector
S99A
25kDa
15kDa
pBADSer99
25kDa
15kDa
BAD
~42kDa
β-ACTIN

C

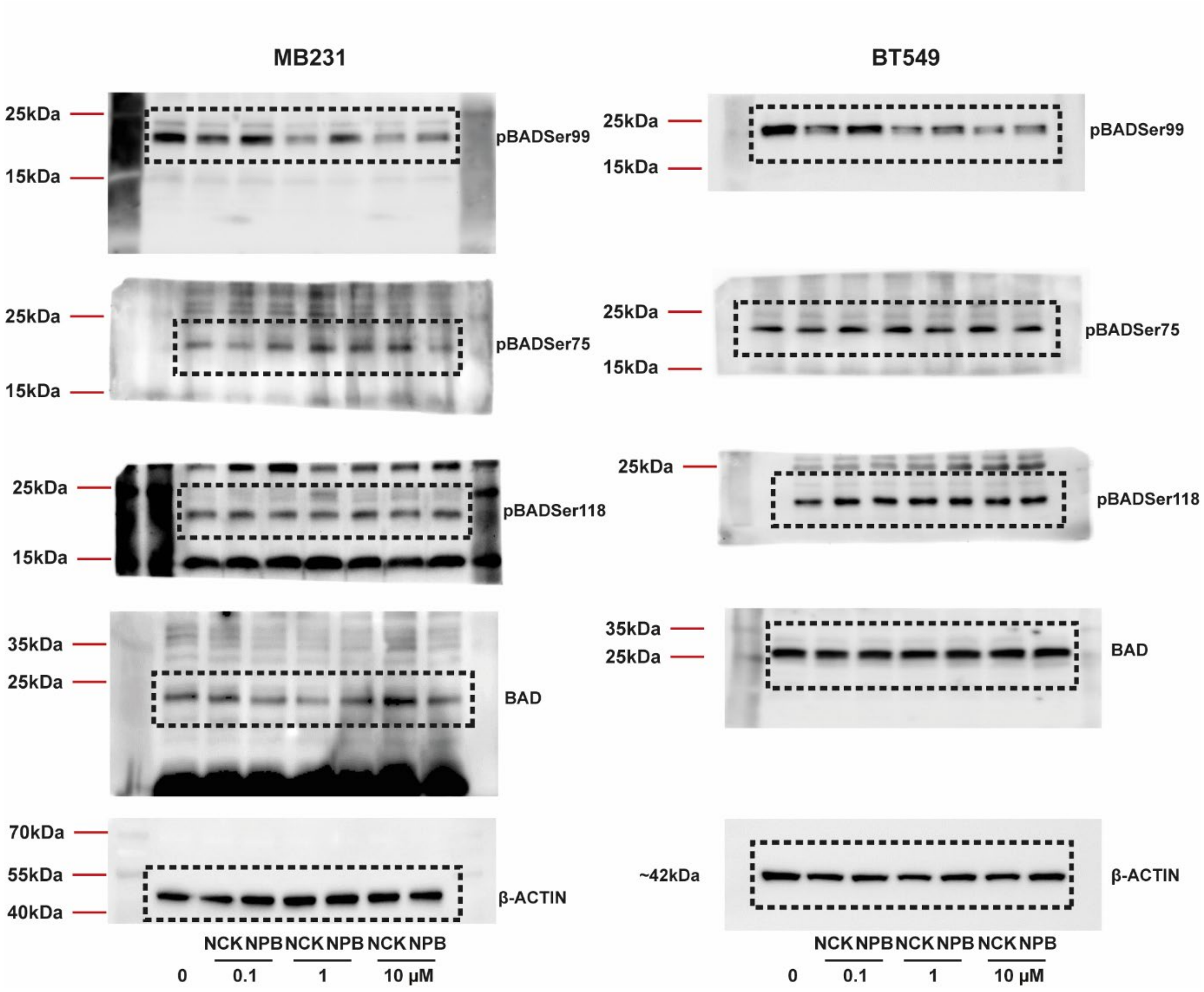
MB231
BT549
25kDa
15kDa
pBADSer99
pBADSer75
pBADSer118
35kDa
BAD
70kDa
55kDa
40kDa
~42kDa
β-ACTIN
NCK NPB NCK NPB NCK NPB
0
0.1
1
10 μM

D

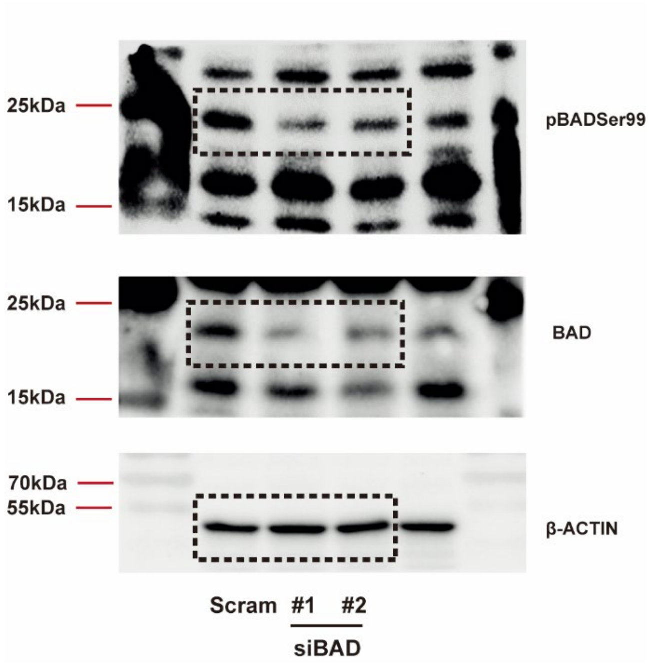
25kDa
15kDa
pBADSer99
BAD
70kDa
55kDa
β-ACTIN
Scram
#1
#2
siBAD

E

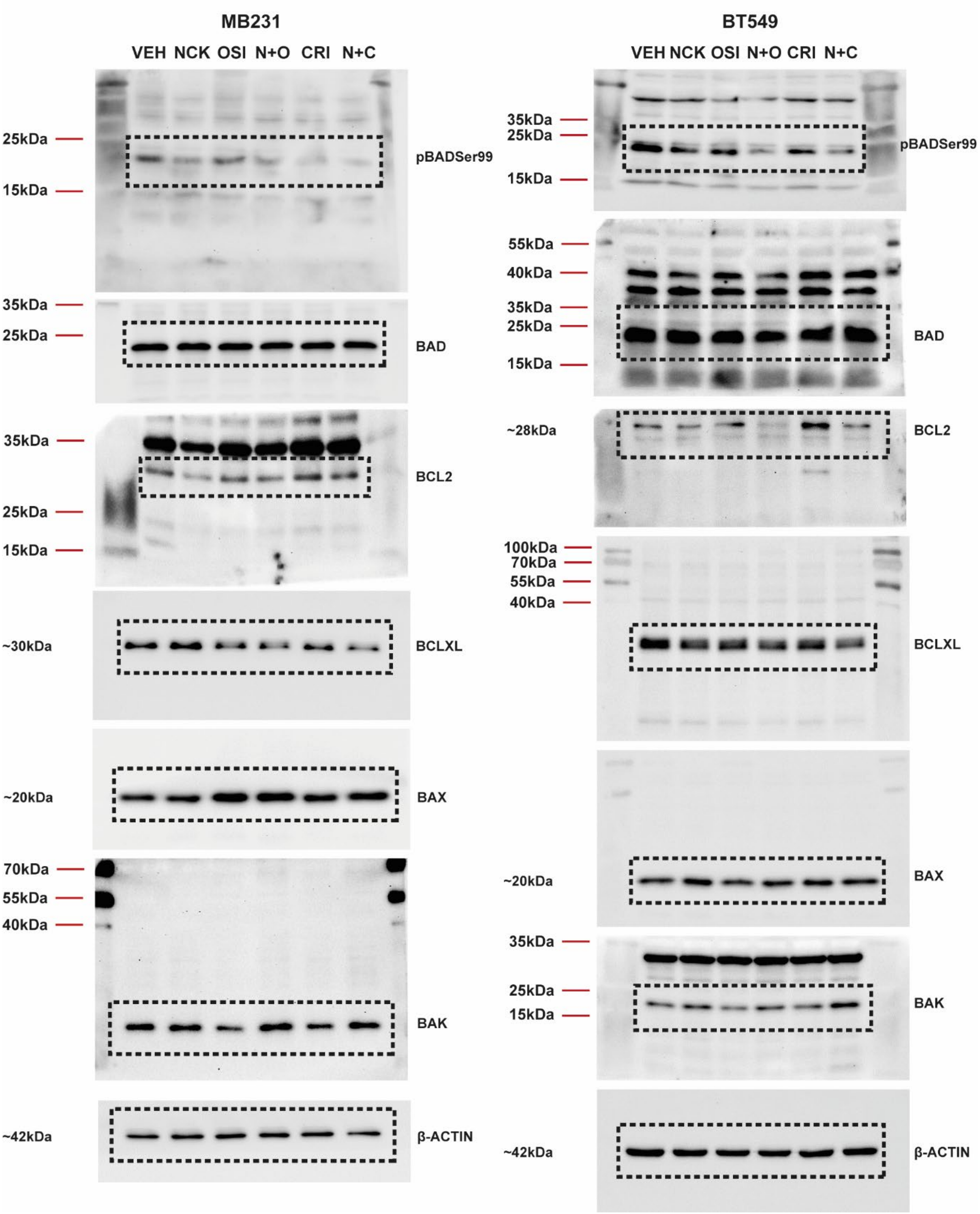
MB231
VEH NCK OSI N+O CRI N+C
25kDa
15kDa
pBADSer99
35kDa
25kDa
BAD
35kDa
BCL2
25kDa
15kDa
~30kDa
BCLXL
~20kDa
BAX
70kDa
55kDa
40kDa
BAK
~42kDa
β-ACTIN
BT549
VEH NCK OSI N+O CRI N+C
35kDa
25kDa
15kDa
pBADSer99
55kDa
40kDa
35kDa
25kDa
15kDa
BAD
~28kDa
BCL2
100kDa
70kDa
55kDa
40kDa
BCLXL
~20kDa
BAX
35kDa
25kDa
15kDa
BAK
~42kDa
β-ACTIN

F

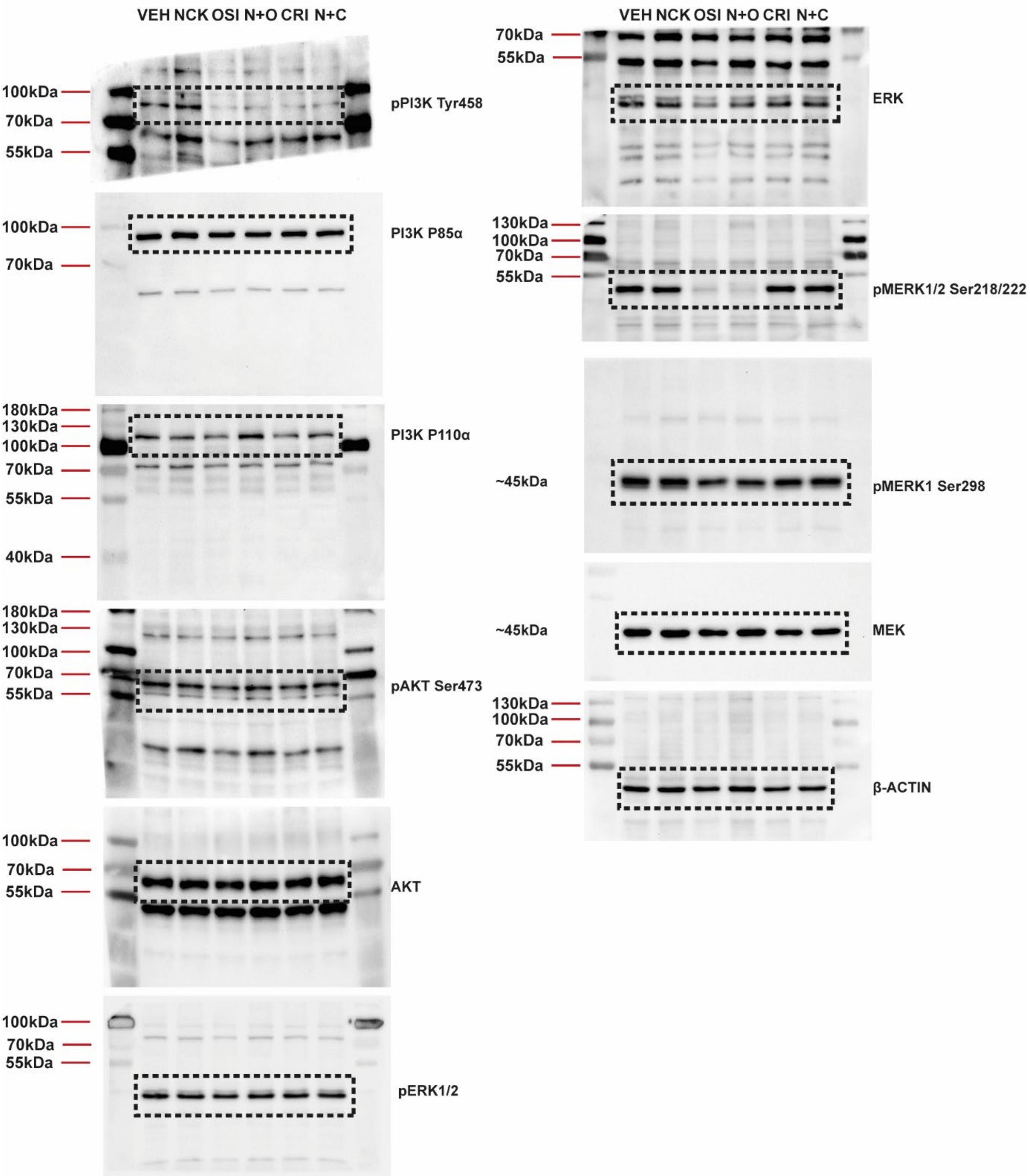

VEH NCK OSI N+O CRI N+C
100kDa
70kDa
55kDa
pPI3K Tyr458
100kDa
70kDa
PI3K P85α
180kDa
130kDa
100kDa
70kDa
55kDa
40kDa
PI3K P110α
180kDa
130kDa
100kDa
70kDa
55kDa
pAKT Ser473
100kDa
70kDa
55kDa
AKT
100kDa
70kDa
55kDa
pERK1/2
VEH NCK OSI N+O CRI N+C
70kDa
55kDa
ERK
130kDa
100kDa
70kDa
55kDa
pMERK1/2 Ser218/222
~45kDa
pMERK1 Ser298
~45kDa
MEK
130kDa
100kDa
70kDa
55kDa
β-ACTIN

G

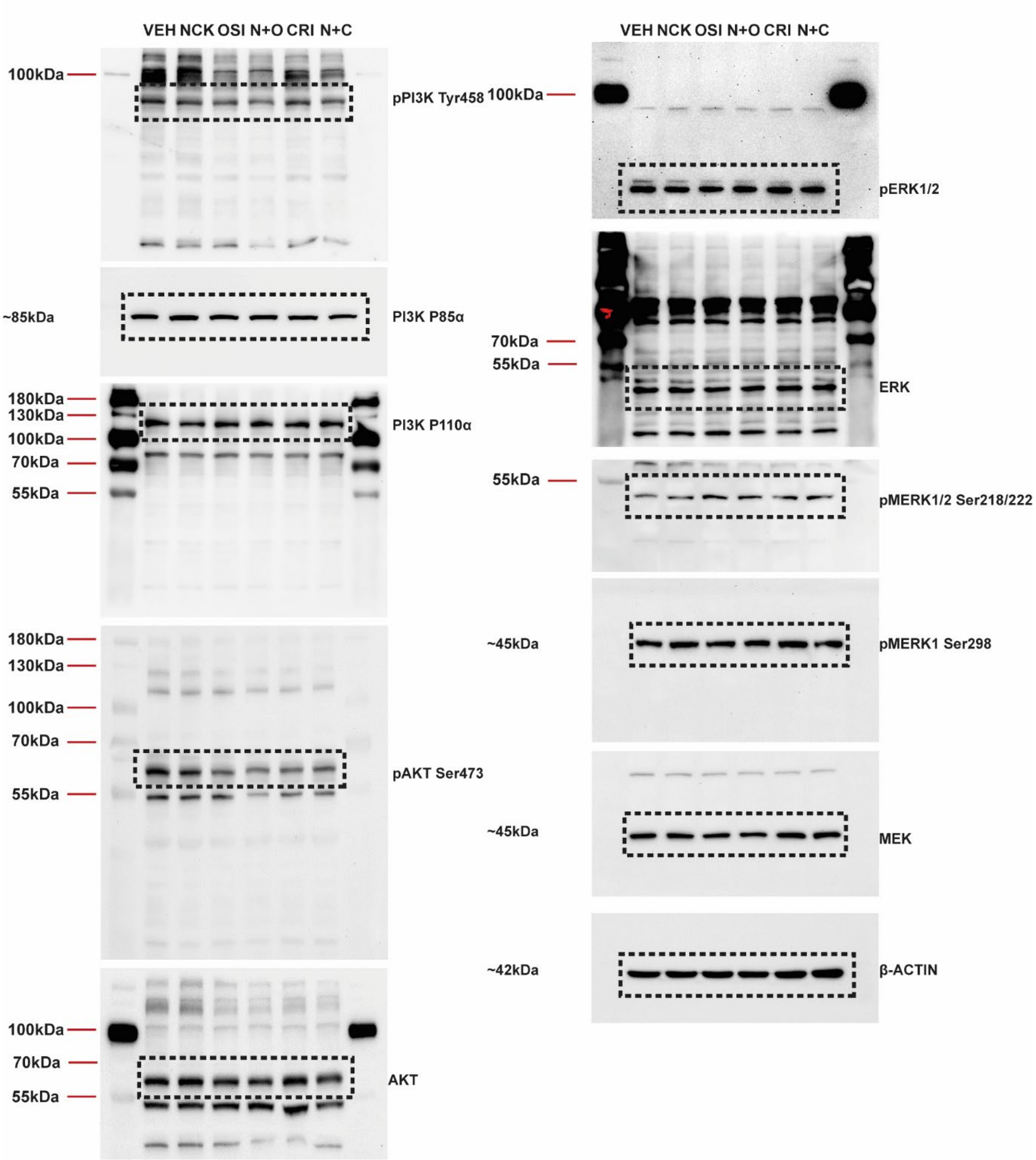

VEH NCK OSI N+O CRI N+C
100kDa
pPI3K Tyr458
~85kDa
PI3K P85α
180kDa
130kDa
100kDa
70kDa
55kDa
PI3K P110α
180kDa
130kDa
100kDa
70kDa
pAKT Ser473
55kDa
100kDa
70kDa
AKT
55kDa
VEH NCK OSI N+O CRI N+C
100kDa
pERK1/2
70kDa
55kDa
ERK
55kDa
pMERK1/2 Ser218/222
~45kDa
pMERK1 Ser298
~45kDa
MEK
~42kDa
β-ACTIN

H

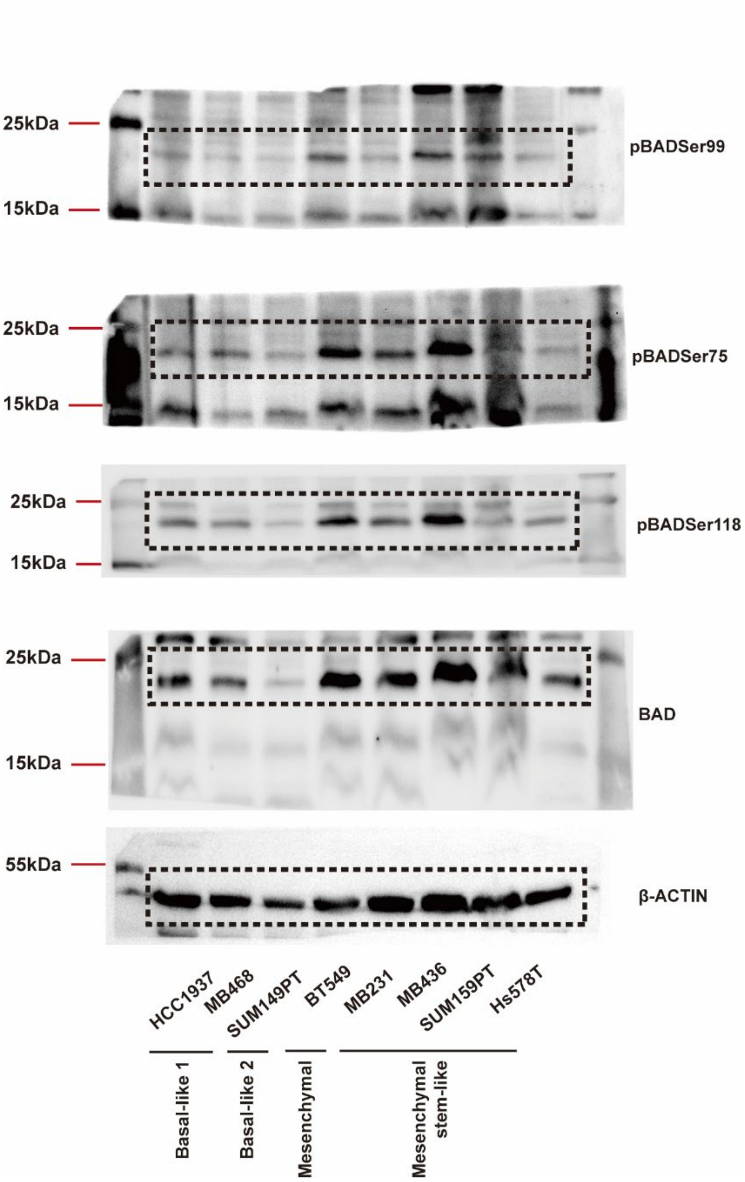

Supplementary Figure 22: (A) Original western blot images for Figure 1B. Corresponding input controls have been provided. (B) Original western blot images for Figure 1C. Corresponding input controls have been provided. (C) Original western blot images for Figure 2E. Corresponding input controls have been provided. (D) Original western blot images for Figure 2G. Corresponding input controls have been provided. (E) Original western blot images for Figure 7E. Corresponding input controls have been provided. (F) Original western blot images for Figure 7F (MB231). Corresponding input controls have been provided. (G) Original western blot images for Figure 7F (BT549). Corresponding input controls have been provided. (H) Original western blot images for Supplementary Figure 6C. Corresponding input controls have been provided.

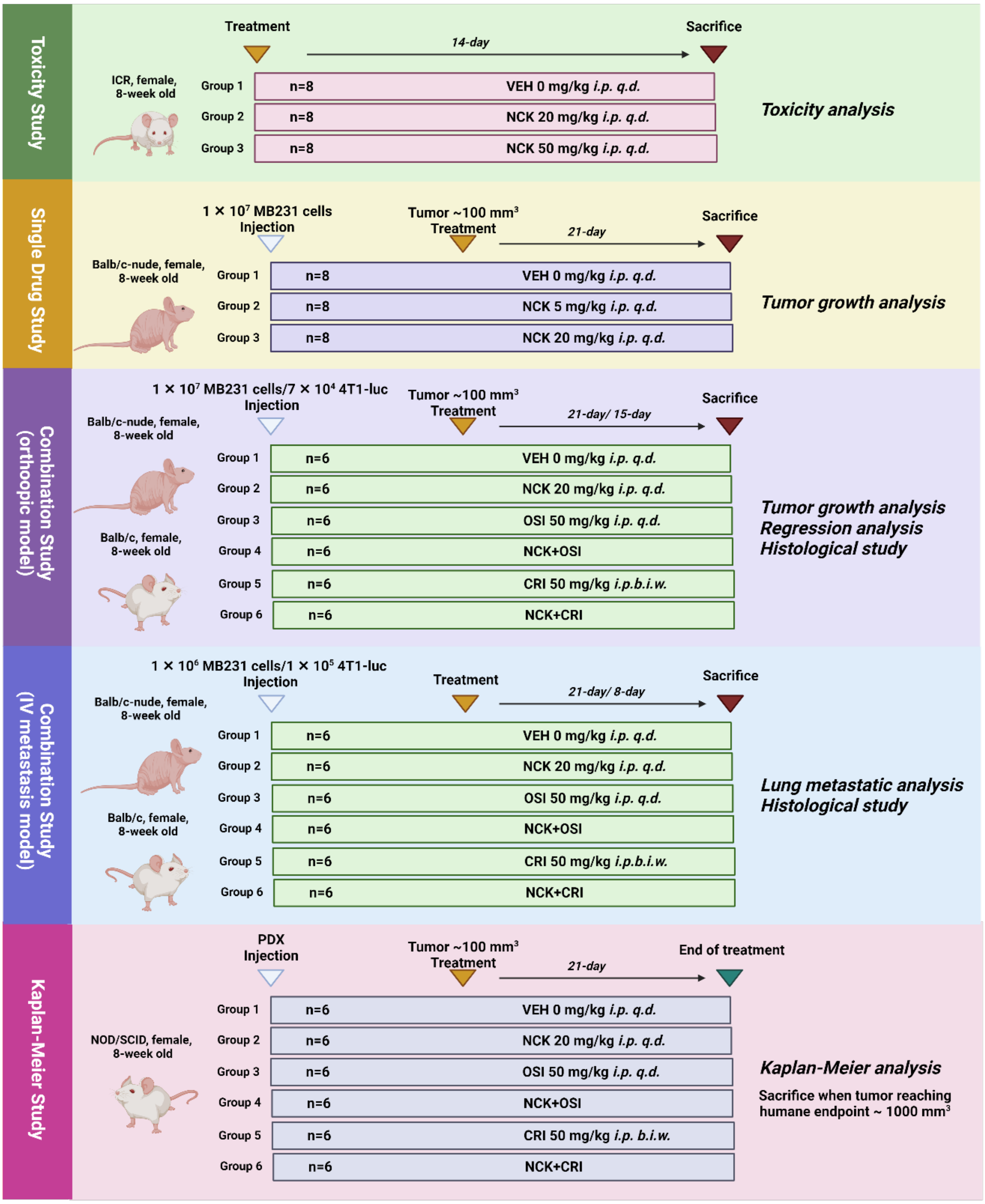

Supplementary Figure 23: Schematic representation of the toxicity study, single drug study, combination study (orthotopic and intravenous metastasis) and Kaplan-Meier analysis. Created with BioRender.com.
